# Statistical optimization of guest uptake in crystalline sponges: grading structural outcomes

**DOI:** 10.1107/S2052252524004871

**Published:** 2024-06-12

**Authors:** Robert C. Carroll, Simon J. Coles

**Affiliations:** a School of Chemistry University of Southampton University Road Southampton HampshireSO17 1BJ United Kingdom; Formby, Liverpool, United Kingdom

**Keywords:** crystalline sponges, statistical design of experiments, metal–organic frameworks, single-crystal X-ray diffraction, SCXRD

## Abstract

Investigation of the analyte soaking conditions on the crystalline sponge {[(ZnI_2_)_3_(tpt)_2_·*x*(solvent)]_*n*_} method using a statistical design of experiments model has provided fundamental insights into the influence of experimental variables. This approach focuses on a single analyte tested via 60 experiments (20 unique conditions) to identify the main effects for success and overall guest structure quality. This is employed as a basis for the development of a novel molecular structure grading system that enables the quantification of guest exchange quality.

## Abstract 2

A statistical design of experiments approach applied to crystalline sponge analysis enables greater understanding of experimental influences and the development of a novel molecular structure quality grading system.

## 1. Introduction

Since its initial development and publication in 2013, the crystalline sponge (CS) method has enabled analysis of non-crystalline compounds using single-crystal X-ray diffraction (SCXRD) (Inokuma *et al.*, 2013 ▸; Hoshino *et al.*, 2016 ▸). The technique employs crystalline, porous, metal–organic frameworks (MOFs) as a host into which small molecules can diffuse. Guest molecules adopt regular positions within the 3D framework, thereby providing the long-range ordering necessary for characterization via SCXRD, which provides atomic resolution molecular structure results. The key benefit of the method is compatibility with SCXRD analysis without the need for sample crystallization, which can take months for some molecules and may be impossible for others.

The first MOFs identified for use as CS hosts were the {[(Zn*X*_2_)_3_(tpt)_2_·*x*(solvent)]_*n*_} series, where *X* = Cl, Br and I, and tpt = 2,4,6-tris­(pyridin-4-yl)-1,3,5-triazine. The significant interest generated by this MOF has led to rapid expansion in the number of host systems identified as suitable for CS analysis. This has included covalent organic frameworks (COFs) and hydrogen-bonded frameworks with systems increasingly being developed for use with specific classes of compounds (Inokuma *et al.*, 2016 ▸; Ning *et al.*, 2016 ▸; Li *et al.*, 2019 ▸; Lunn *et al.*, 2020 ▸; Chen *et al.*, 2022 ▸; Wada *et al.*, 2024 ▸).

To date, the relatively generic applicability of the Zn*X*_2_-tpt hosts has great versatility with a wide range of chemistries and means they are the most widely used for the CS technique. [Evidenced by work within our group, which has generated a CSD subset for CS structures, akin to that of the MOF subset available from CCDC (Moghadam *et al.*, 2017 ▸).] The compatibility of the host with a wide range of guests can be rationalized by its intermolecular interactions: host–guest contacts can occur with pyridine and triazine rings, as well as Zn*X*_2_ units. The variety of potential interactions enables guests to adopt regular positions within the pore without the requirement for specific chemical functionality. Some of the most notable applications have included the combination of CS with high-performance liquid chromatography for analysis of human metabolites, or the study of natural products that requires only nanograms of sample for molecular structure elucidation (Rosenberger *et al.*, 2020 ▸; Inokuma *et al.*, 2013 ▸). Two extensive recent reviews further discuss the underlying principles and applications of CS analysis (Zigon *et al.*, 2021 ▸; Habib *et al.*, 2022 ▸).

Although the CS method has added another tool to the arsenal of advanced crystallization methods, uptake has been limited to a handful of laboratories globally (Metherall *et al.*, 2023 ▸). Significant proportions of work undertaken on CS analysis have been devoted to method development, including more efficient synthesis of host crystals, optimization of hardware and improvements to guest exchange strategies for reactive guests (Waldhart *et al.*, 2016 ▸; Meurer *et al.*, 2022 ▸; Sakurai *et al.*, 2017 ▸). This branch of research has also examined the reliability of host–guest interactions to improve our understanding of analyte and host behaviours (Hayes *et al.*, 2016 ▸; Carroll *et al.*, 2023 ▸).

While these developments have enabled more effective use of the CS method for experienced crystallographers, there are still several issues which prevent its wider adoption. Two examples of these difficulties are optimization of guest-exchange conditions and quantifying the quality of the results.

**Figure 1:**
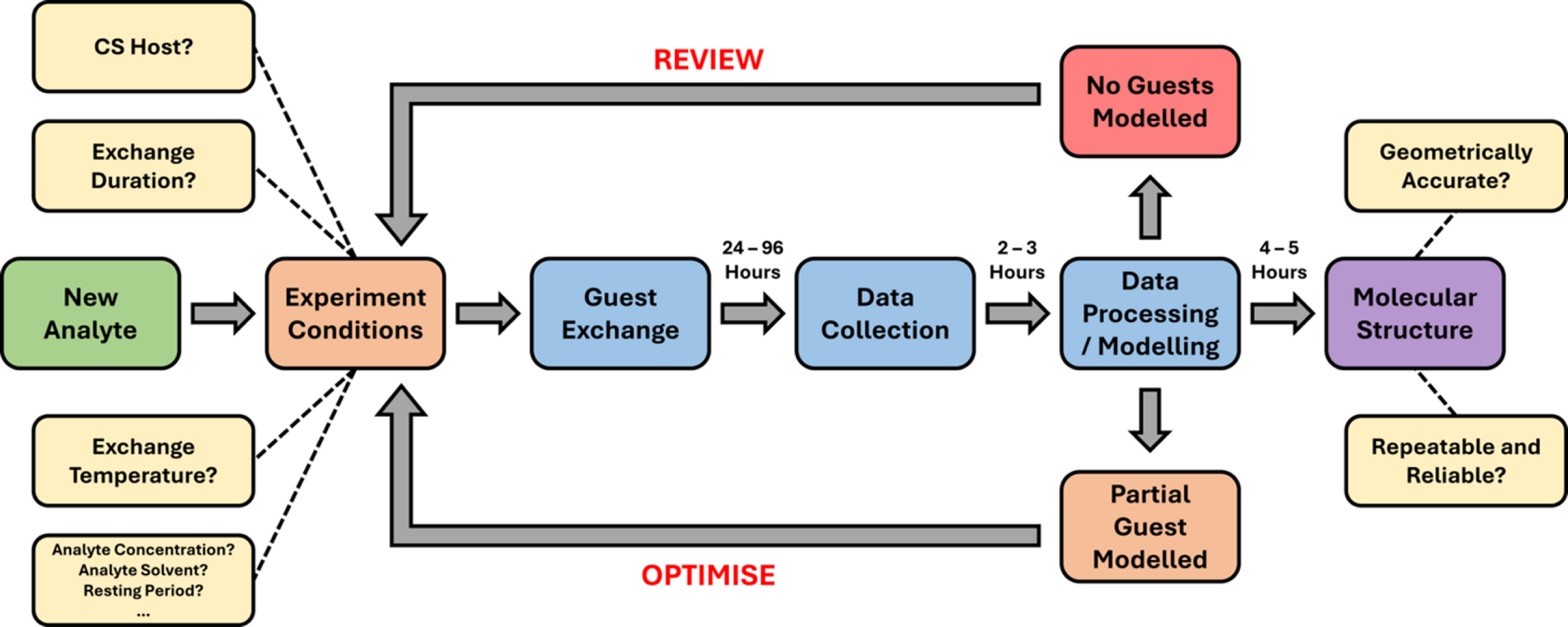
Workflow of the iterative experimental approach for the CS method.

The challenge of optimizing guest-exchange conditions for CS is best understood in the context of the iterative experimental approach which analytes are subjected to, illustrated in Fig. 1 ▸.

Firstly, a CS host material is selected. This could be any of the range of discovered hosts, but for simplicity in this study is restricted to the ZnI_2_-tpt framework. Choices must then be made regarding host solvent, analyte solvent, analyte concentration, exchange duration and exchange temperature, all of which, from our experience, have an influence on successful exchange and the quality of the result achieved.

Each soaking experiment takes a minimum of 24 h, after which data must be collected, processed and modelled before it can be determined if the outcome was successful or improved upon the previous result. This time-consuming process must then be repeated continuously until the guest compound has been successfully characterized. Often it is possible to at least partially model guests within a few iterations if the analyte is compatible with the CS method, but how does one know if the best possible result has been achieved at any particular stage? This is difficult to assess due to the significant variation of guest-exchange quality between different molecules, often resulting in reliance on intuition rather than quantitative indicators. Assessment is also complicated by failures, which may be due to host crystal defects rather than the selected experimental conditions, though this may be combatted by running experiments in triplicate to minimize the influence of ‘random’ failures.

Several publications have addressed the issue that no single optimum experimental condition for guest exchange exists for all analytes (Sakurai *et al.*, 2017 ▸; Wada *et al.*, 2021 ▸). Their conclusions are that this is due primarily to variation in chemical reactivities, as well as molecular shape and size. However, is it possible to identify wider trends that can inform iterative experiments to achieve the best exchange in the fewest number of attempts?

To explore this idea, a statistical design of experiments (DoE) study was undertaken to investigate the exchange of a single analyte with 20 unique experimental conditions run in triplicate. DoE methodologies are commonly used in industrial applications, where a high number of variables need to be probed under time and budget constraints (Fisher, 1935 ▸; Yuangyai & Nembhard, 2010 ▸). From a practical perspective, DoE studies plan an array of experiments which vary multiple parameters simultaneously for efficient exploration of the experimental ‘landscape’. This contrasts to traditional iterative experiments which vary one factor at a time (OFAAT) and can produce collections of data that are not well suited for assessing wider trends and correlated effects.

For CS research, this represents the largest statistical study published to date and the extensive collection of data provides a basis for analysis of the three main experimental influences of CS experiments. These factors are exchange temperature, analyte concentration and exchange duration, and have become apparent based on extensive prior experience. The influence of these factors on successful guest exchange has enabled analysis of additive effects, assessing whether the overall impact of the two factors can be described by summation. This was followed by analysis of the crystallographic space group and unit-cell variation as an indicator for guest-exchange quality. Finally, a novel analyte structure grading system was developed to quantitatively assess outcomes with a view to defining when the best possible result has been obtained.

The molecular grading system is integrated into the crystallographic refinement procedure and relates guest geometry to equivalent geometry in structures of ‘pure’ analytes determined by conventional SCXRD. It is designed to be used in combination with accepted crystallographic model validation and quality assessment tools and provides an informative metric that can be used to assess improvements in iterative experimental attempts. The system represents an approach to grading CS structures derived from our standardized method and will require development to extend applicability to more diverse crystallographic structures. The grading system offers non-CS specialists a numerical value as an indicator of the quality, and hence reliability, of the derived molecular structure of the analyte.

## 2. Methodology

**Figure 2:**
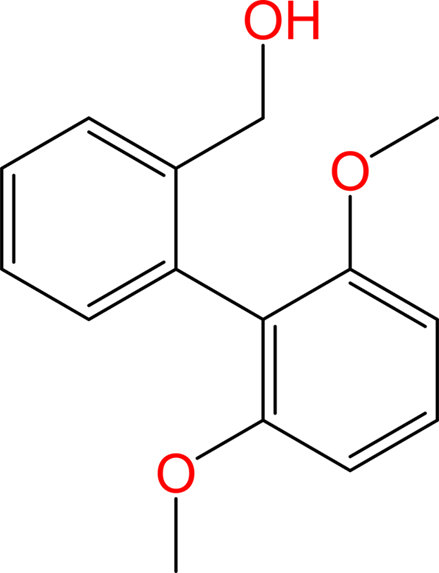
Molecular structure of 2,6-di­meth­oxy-2′-hy­droxy­methyl­biphenyl (BBA-8,12-OMe).

The analyte material selected for this study was 2,6-di­meth­oxy-2′-hy­droxy­methyl­biphenyl and is shown in Fig. 2 ▸. The molecular structure of this compound, as determined by the CS method, was first reported in a previous systematic study and is referred to in both studies as benzyl bi­aryl alcohol – 8,12 – meth­oxy (BBA-8,12-OMe) (Carroll *et al.*, 2023 ▸).

This analyte was chosen because of its efficient uptake into framework pores. The encapsulation process results in a change in crystallographic space group from that of the as-prepared host and enables multiple guest molecules to be modelled inside the framework. Additionally, the SCXRD characterization of a ‘pure’ BBA-8,12-OMe crystal structure supports this study by providing reliable bond distances and angles which act as a foundation for the development of the geometric structure grading system. Future iterations will not rely on conventional SCXRD structures and are addressed in Section 4.

To trial the suitability of BBA-8,12-OMe for wider statistical analysis, a selection of scoping experiments were conducted to identify suitable design variables for the three main experimental factors: exchange temperature, analyte concentration and exchange duration. The factor levels chosen (shown in Table 1 ▸) are based on a mixture of scoping experiments and published conditions.

Crucially, the selected factor levels exhibit the necessary amount of variation for guest-exchange efficiency and molecular geometric accuracy of BBA-8,12-OMe, such that results are distinguishable. The influence of these factors on both the success and the quality of guest exchange has been highlighted in previous publications (Sakurai *et al.*, 2017 ▸). Note that host crystal size is believed to influence the observed results; this factor has not been accounted for in this work but is being investigated in detail in a separate study (see Section 4). The response variables or measured outcomes explored in this study were: the ability to resolve a guest molecule, the crystallographic space group/unit cell adopted and a numerical grade related to the quality of the guest-molecule model.

There can be multiple interpretations of what constitutes an ‘optimal’ condition depending on the researcher’s aims. For this study, we have chosen to target conditions that produce consistent successful results across all replicates and enable modelling of multiple guests with the best average grade.

The combination of three levels for each experimental factor would result in 27 unique experimental conditions and 81 total experiments when repeated in triplicate. This multilevel factorial study was reduced in size using the *Minitab* software and the generation of a factorial design selected according to D-optimality (Minitab LLC, 2021). Three optimal designs were generated for comparison and the corresponding statistical values are tabulated in Section S1 of the supporting information. It was determined that a study with 20 unique experiments run in triplicate (60 total experiments) represented the best compromise between statistical insight for two-factor influences and feasibility with time/resources. Trials for each replicate were randomized to reduce the confounding of uncontrolled and controlled factors.

The full list of experimental conditions is tabulated in Section S1 and further discussion of experimental procedure can be found in Sections S2 and S3. Crystallographic tables for all structures of BBA-8,12-OMe are shown in Section S5.

Assignment of structural grades for guests relied on geometric accuracy compared with conventional SCXRD data. It was possible to obtain suitable crystals of BBA-8,12-OMe via the encapsulated nanodroplet crystallisation (ENaCt) method, an advanced crystallization technique for small organic molecules (Tyler *et al.*, 2020 ▸).

**Figure 3:**
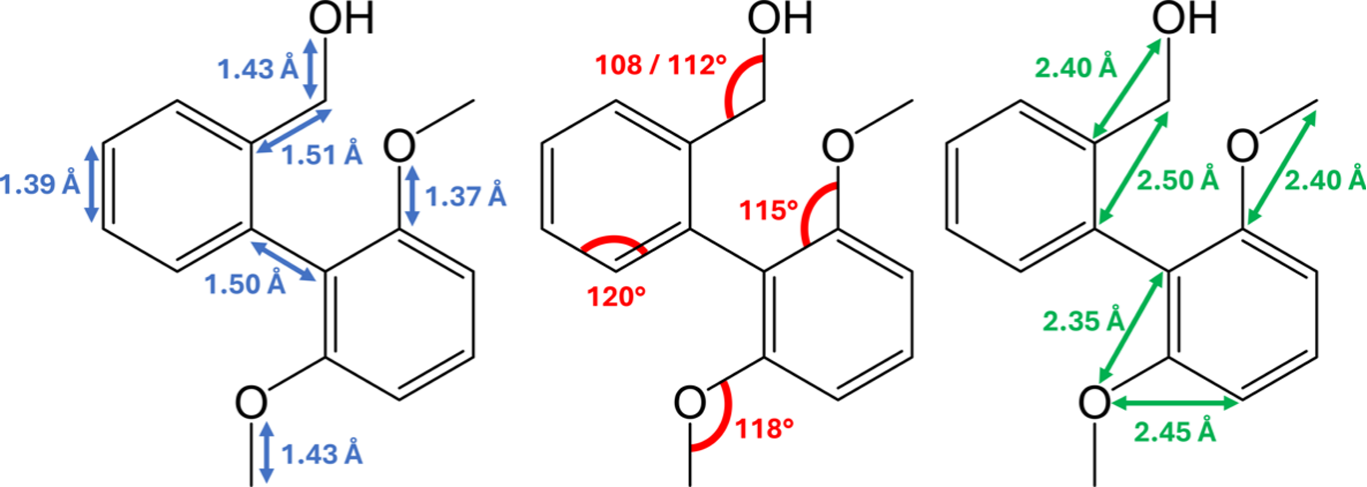
Geometric values for BBA-8,12-OMe determined by conventional SCXRD (CCDC No. 2341226).

Guest molecules modelled within the CS framework often possess distortions of molecular geometries and significantly larger thermal ellipsoids than conventional SCXRD structures. To ensure adherence to the more reliably determined geometries, crystallographic restraints and constraints were applied to CS guest molecules. Restraints and constraints were applied consistently and in a progressive manner to all structures. The relevant target values for bond distances and angles are shown in Fig. 3 ▸.

The stepwise application of restraints and constraints was essential for application to a grading system. The full details of the grading system, including a breakdown of the values assigned to restraints/constraints and an illustrative example, can be found in Section S4. In summary, individual guest-exchange sites are scored from 0.00 to 16.00, with lower scores representing higher quality data and a closer resemblance to conventional SCXRD data. Scores are additive, summing values attributed to bond geometries, crystallographic restraints/constraints and thermal parameters. The structural grading system has been successfully implemented for similar CS bi­aryl structures and other small molecules, highlighting its scope for application to all CS results, and potentially beyond.

## 3. Results and discussion

### 3.1. Guest-exchange rates and reliability

**Figure 4:**
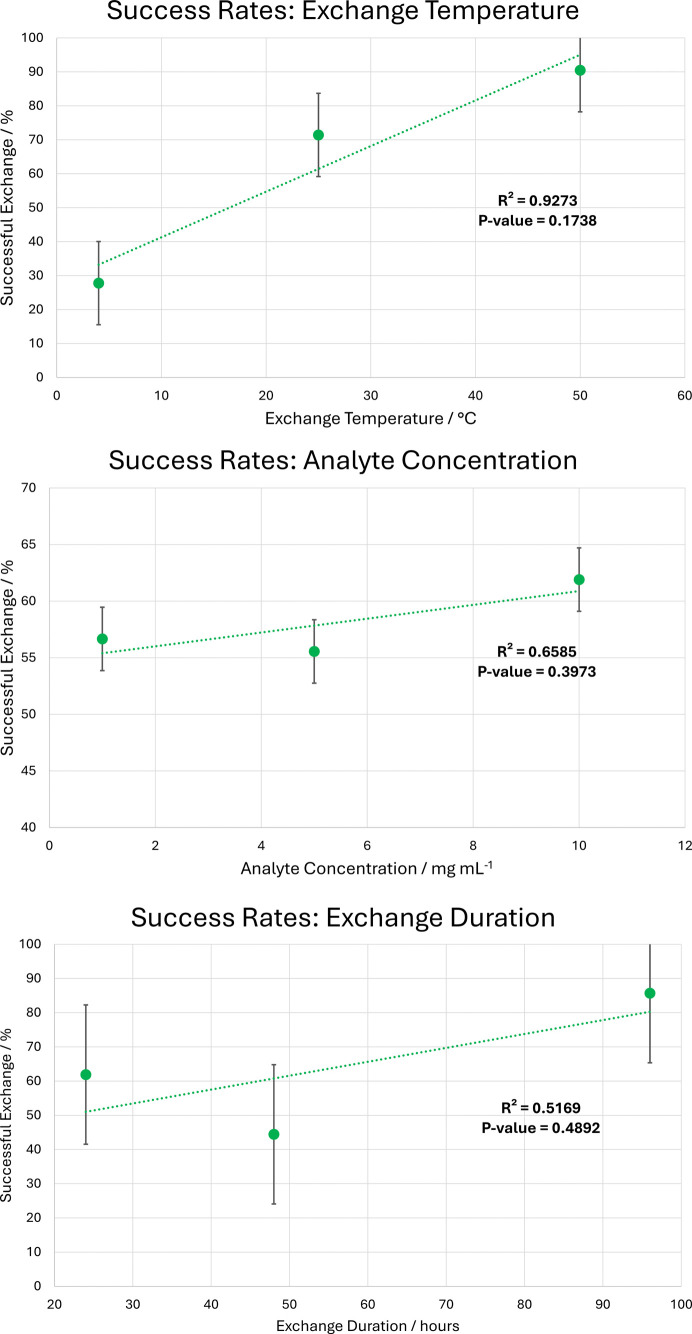
Single factor success rates for BBA-8,12-OMe guest exchange.

With the results of all 60 experiments, the ability to model BBA-8,12-OMe guests within the framework could be assessed. As the first response variable explored in the study, this metric offers an assessment of reliability for CS experiments. The success rate of experiments with respect to individual factors are illustrated in Fig. 4 ▸.

The first point of note is the relatively high number of failed guest exchanges: more than one third (35%) of experimental conditions resulted in data where no molecules of BBA-8,12-OMe could be resolved. Most of these failed experiments were associated with low exchange temperature (4°C) or middle exchange duration (48 h). It is notable that even for BBA-8,12-OMe, an analyte which has been identified as well suited to systematic study, there is still considerable inconsistency or variability in exchange into the CS host.

Investigation of trends for the three experimental factors highlights that exchange temperature appears to be the main effect, exhibiting a positive correlation between an increase in temperature and the ability to resolve guest molecules within the pore. This could be attributed to an increase in kinetic energy which drives more complete diffusion of BBA-8,12-OMe into the host framework. However, the relatively high *P*-value (0.1738) indicates that this might not be statistically significant, and it is therefore not possible to reject that this trend is a product of random error.

In comparison, analyte concentration and exchange duration exhibit a greater deviation away from linear positive correlations and have significantly higher accompanying *P*-values. Consequently, no statistically meaningful trends can be extrapolated from the influence of these single factors. To improve the insight into individual factors, more factor levels would be required.

The interplay of the three factors on successful exchange can be examined further with consideration of two factor influences, illustrated in Section S6. Most of these plots possess no clear correlation between successful exchange and relevant factors, this is further shown with regression statistics that cannot statistically validate apparent trends. These plots highlight the difficulty in assessing wider trends for CS data; experiments conducted under the same conditions can often have unreliable results and the resulting statistical metrics, such as *P*-values, are sensitive to this variability.

However, some insights are available for specific combinations of two-factor influences. For example, there is a strong positive correlation of temperature with low (1 mg ml^−1^) analyte concentration, this suggests again that increased temperature and kinetic energy can be beneficial for more complete diffusion of analyte material. Similarly, longer exchange durations displayed a positive correlation at a 25°C exchange temperature. This may suggest that systems with lower kinetic energy may rely more heavily on longer exchange durations for successful diffusion and settling within the pores. Both trends are supported by *P*-values < 0.05, which indicate that it is unlikely these are observed because of random error and therefore their corresponding null hypotheses may be rejected.

Analysis of two-factor influences also enables some specific identification of favourable and unfavourable conditions for exchange (see Table 2 ▸).

### 3.2. Crystallographic space groups

Having understood the influence of experimental factors on successful guest exchange, the second response variable – categorization of crystallographic space group and unit cells – is introduced. There are three classes of result: *C*2/*c* ≃ 16000 Å^3^, *P*2/*n* ≃ 16000 Å^3^ and *C*2/*c* ≃ 48000 Å^3^. The unique forms can be analysed in combination with the design variables discussed in Section 3.1 and, furthermore, can be related to the efficiency of guest uptake or overall quality of guest exchange. To understand each of these crystallographic results, structural differences must be considered alongside their relationship to experimental variables.

**Figure 5:**
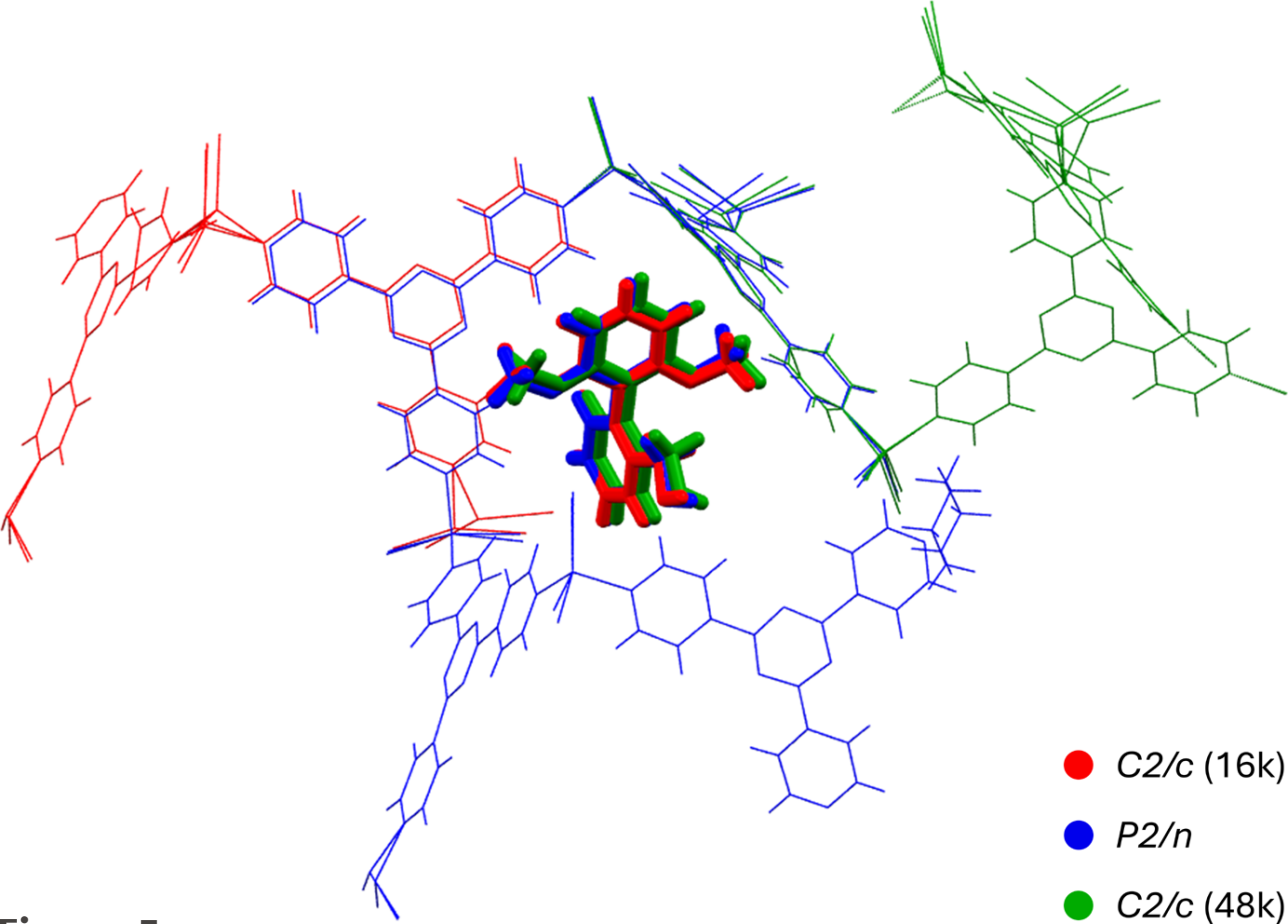
Representative overlay of primary guest sites and host frameworks for the three crystallographic forms. *C*2/*c* (16k): green; *P*2/*n*: blue; *C*2/*c* (48k): red.

Overlay of representative structures from each unique form indicates that relatively consistent locations and geometries were adopted by guests in the primary exchange site of all crystallographic forms. This was further corroborated by analysis of intermolecular interactions between the analyte molecule and the host framework, with full interaction tabulation presented in Section S9. An overlay of representative structures from each category is shown in Fig. 5 ▸.

Variation between some of the crystallographic forms is more apparent through consideration of the number of guests resolved within the pore and the relative occupancy of these exchange sites, as summarized in Table 3 ▸.

*C*2/*c* (16k) possesses the greatest similarity to an unexchanged and solvent-filled ZnI_2_-tpt host. There is some expansion of the unit-cell volume (∼600 Å^3^), enabled by the breathable and interpenetrated nature of the host framework, but only one low-occupancy guest site is present.

In contrast, the *P*2/*n* and *C*2/*c* (48k) forms showcase more complete exchange through greater occupancy of the primary site and the potential to resolve a weaker secondary site. For *C*2/*c* (48k) there is also the potential for a unique tertiary site, closely related to the secondary site, but this is only observed once throughout the entire study.

To rationalize the differences between the *P*2/*n* and *C*2/*c* (48k) forms, a more detailed consideration of their respective secondary site locations and interactions is required. The full crystallographic analysis for these sites is presented in Section S7. In summary, the *P*2/*n* form possesses a secondary guest site which is located over a twofold symmetry axis and prioritizes interaction with the host framework. Conversely, the *C*2/*c* (48k) secondary site adopts a slightly different orientation which maximizes interaction with neighbouring guests and leads to adoption of the supercell form. Importantly, the location of the *P*2/*n* secondary guest necessitates additional crystallographic restraints and constraints, the consequences of which are explored further in Section 3.3.

**Figure 6:**
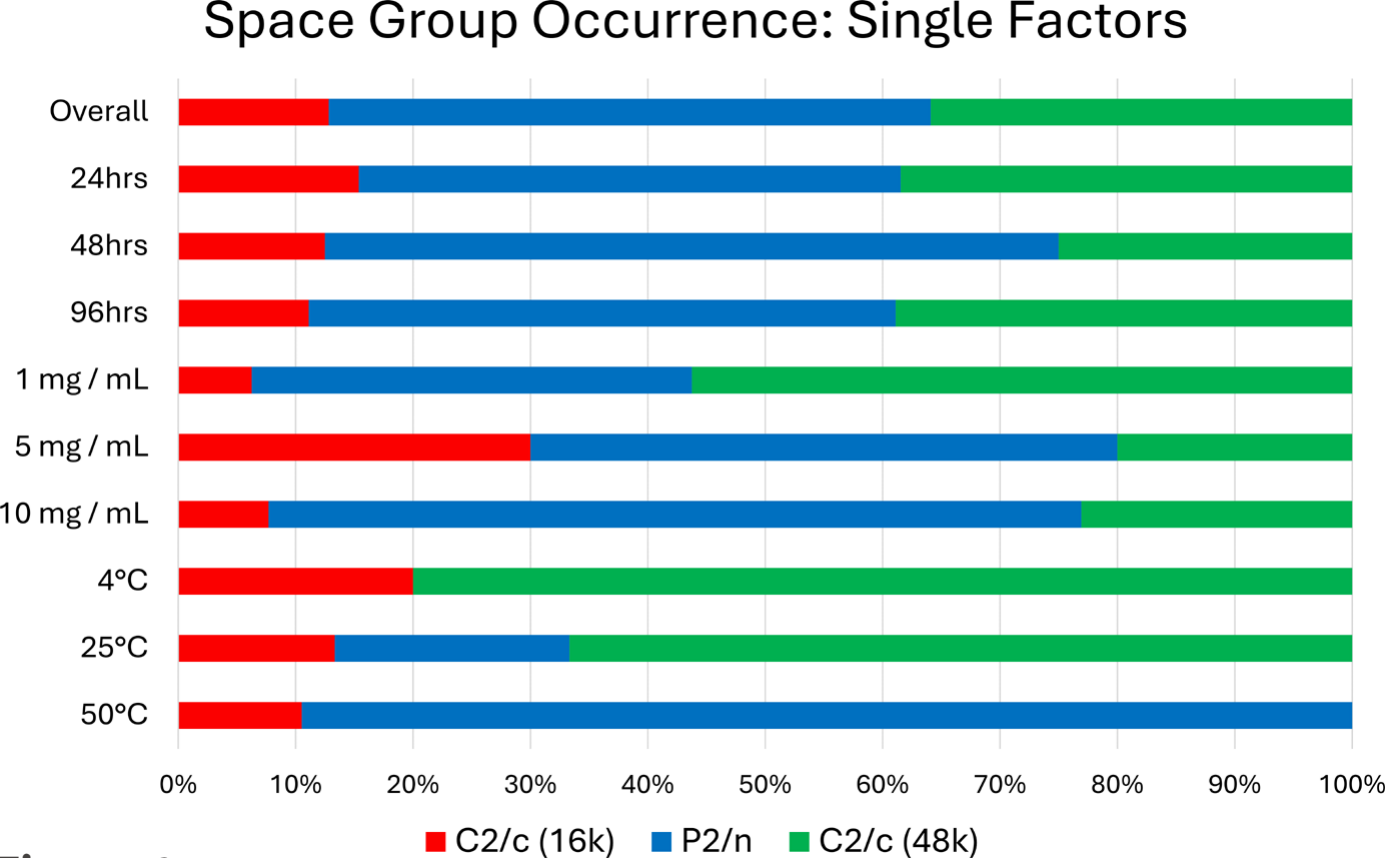
Overall and single factor influences for crystallographic form adoption.

Once structural differences between the three crystallographic forms have been established, their relationships to experimental factors can be explored, as illustrated in Fig. 6 ▸.

Overall, *P*2/*n* is the most common result with over half (51.3%) of the successful BBA-8,12-OMe exchanges adopting this form. This is followed by *C*2/*c* (48k) with 35.9% and finally *C*2/*c* (16k) with 12.8%. Similar to the trend for single factors (Fig. 4 ▸), the exchange temperature exhibits the most interesting influence. At higher temperature (50°C), *P*2/*n* is the overwhelmingly dominant form with *C*2/*c* (16k) only adopted 10.5% of the time and *C*2/*c* (48k) not present at all. There is then a major decrease in the *P*2/*n* form with the step to 25°C, this is primarily replaced with *C*2/*c* (48k) and this trend continues to 4°C where *P*2/*n* is no longer observed.

As previously discussed, *P*2/*n* and *C*2/*c* (48k) have comparable guest-exchange occupancies but differ in structural features of the secondary exchange sites. The correlation with temperature therefore suggests there is a kinetic barrier to adoption of the secondary site which interacts more reliably with the CS host and results in the *P*2/*n* form.

Although not the main effect for occurrence of crystallographic space groups, analyte concentration does have a direct relation to observation of the *P*2/*n* form. This follows the same trend as exchange temperature, with higher analyte concentration producing more *P*2/*n* structures. It may be inferred that a greater concentration gradient of the analyte surrounding the host crystal can also influence the diffusion mechanism of guest molecules into the CS host.

The classification of guest exchange into three unique forms presents another opportunity to assess the reliability of CS experiments: of the 20 unique experimental conditions, how many produced the same crystallographic result with the same number of guest molecules resolved?

Only two experiments achieved the same result for all three of the triplicate runs, these were 50°C|10 mg ml^−1^|24 h and 50°C|10 mg ml^−1^|96 h. An additional three experiments possessed the same crystallographic form but with variation in the number of guests resolved and these were 50°C|1 mg ml^−1^|96 h, 50°C|5 mg ml^−1^|96 h and 25°C|1 mg ml^−1^|96 h. This again highlights that higher temperature and longer duration exchanges have greater reliability for success and consistent results. On the other hand, it demonstrates the very varied behaviour of CS experiments when conducted on a larger scale.

### 3.3. Quantitative structural grading system

Although crystallographic space group classification enables identification of differences, it does not convey the variation in quality between results. A quantitative structural grading system was therefore devised to distinguish between CS structure quality. The primary aim of this method is to present a simple and comparable metric, such that quality of data can be understood without having to factor in the guest exchange, data collection and model refinement stages.

Though a full description of the structural grading system can be found in Section S4, comparison of grades for *P*2/*n* and *C*2/*c* (48k) secondary sites highlights a useful application of the system. As shown previously in Table 3 ▸, these two crystallographic forms appear remarkably similar when considering efficiency of guest loading. However, the secondary exchange site for the *P*2/*n* form is located over a twofold symmetry axis. To model this site, considerably more restraints and constraints are required in comparison with *C*2/*c* (48k) and the exchange location also often results in the expansion or flattening of atomic displacement parameters.

**Figure 7:**
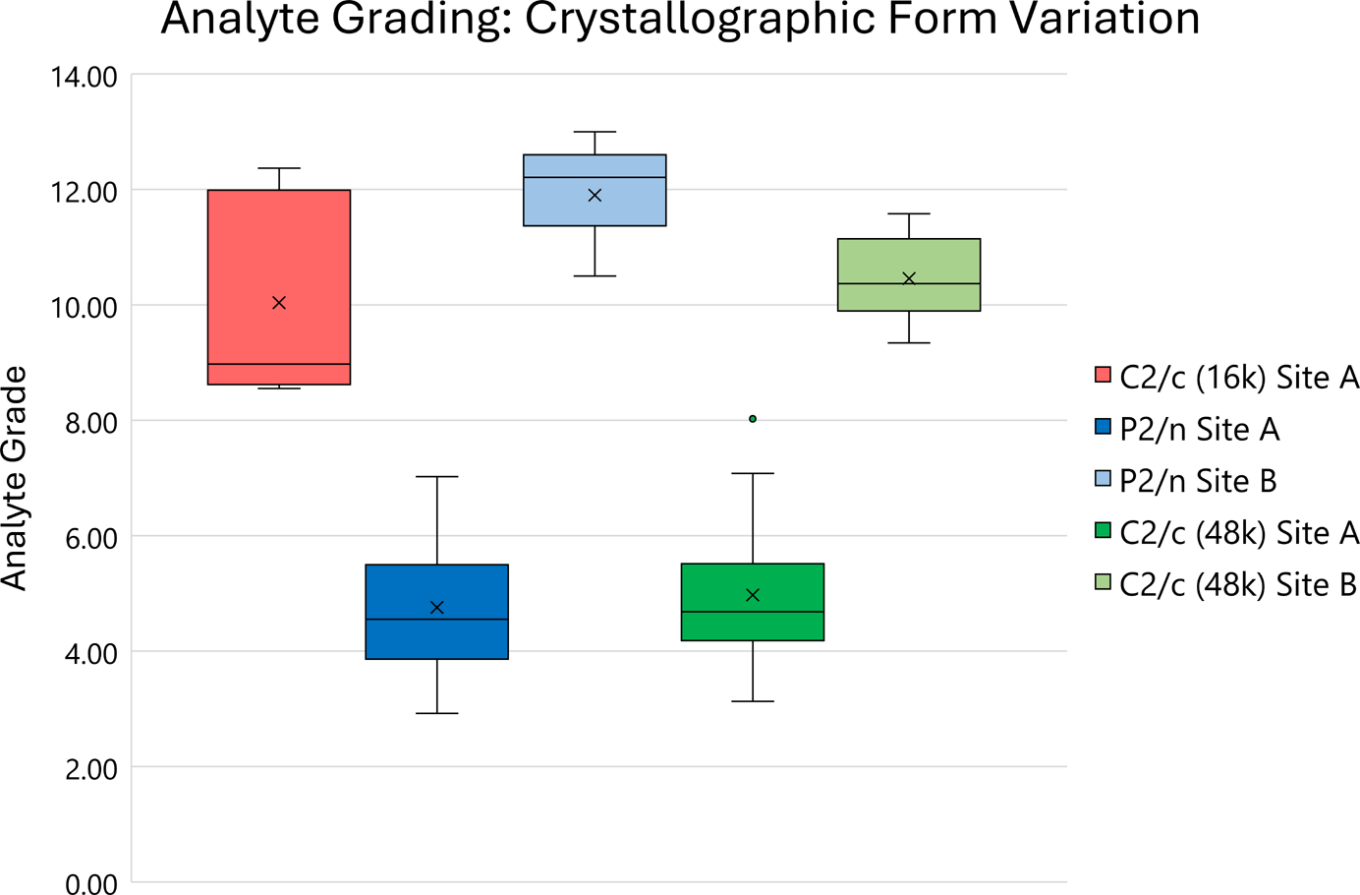
Box and whisker plot of analyte grades for the three crystallographic forms and their unique exchange sites.

These additional restraints and constraints are represented graphically in the supporting information, but it is difficult to quantify the difference in structure quality between sites in this form. In contrast, through application of the structural grading system, the quality of the relevant secondary site molecular structures (and in fact all guest exchanges) can be differentiated relatively easily as illustrated in Fig. 7 ▸. Full tabulation of analyte grades is shown in Section S10.

This clearly showcases that *C*2/*c* (48k) secondary site structures are better defined and have a closer resemblance to conventional SCXRD structures. The extent to which these grades reflect the physical nature of the exchange sites can also be considered. The intervention with restraints and constraints is required for a standardized and extremely useful asymmetric unit representation. However, the frequency at which *P*2/*n* and *C*2/*c* secondary sites occur suggests that *P*2/*n* exhibits the more reliable exchange.

For *P*2/*n*, secondary site structures are observed 80% of the time, in comparison with only 40% for *C*2/*c* (48k) structures. The high occurrence of *P*2/*n* secondary site structures and their more reliable contact with the host framework (discussed in Section S7) suggests this site is better positionally stabilized. To investigate this further, analysis of interaction energies with the relevant host/guest molecules would be required and this is the subject of ongoing studies.

The grades for *P*2/*n* and *C*2/*c* (48k) primary exchange site structures are considerably better than those in *C*2/*c* (16k), which has a closer similarity to their respective secondary exchange site structures. This reflects the guest-exchange occupancy previously shown in Table 3 ▸ and, while this is influenced by the inclusion of occupancy as a quality indicator, it also highlights that sites with more complete exchange tend to have more accurate geometric features.

**Figure 8:**
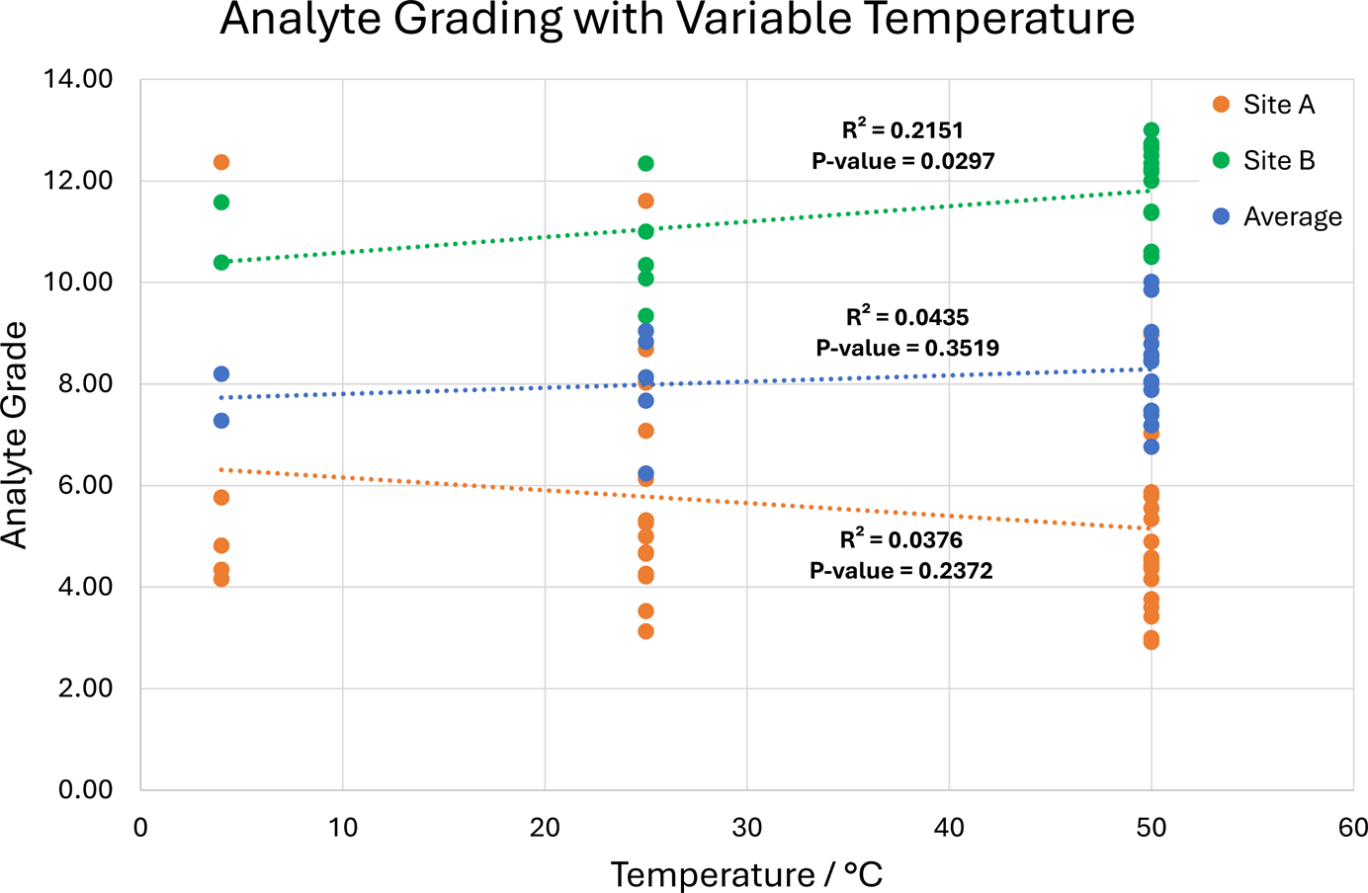
Scatterplot of analyte-grade relationship to variable exchange temperature.

To further explore the viability of the structural grading system, evaluation with respect to single experimental factors was undertaken. This results in good agreement with conclusions drawn from both success rate and crystallographic form plots as highlighted in Figs. 8 ▸ and 9 ▸.

These show that an increase in temperature results in worse secondary exchange site structure quality. This is primarily driven by the dominance of the *P*2/*n* form at higher temperature and the subsequent practical difficulty of modelling the guest over a twofold symmetry axis.

Further investigation of analyte grades with single and two-factor experimental influences highlights the mixed relationship between consistency of guest-exchange and guest-model quality. Although 50°C experiments have reliable guest exchange and exhibit the highest quality primary sites, 48 h experiments, which are rarely successful, achieve the best primary site grades of all three exchange durations explored in this study (Section S11).

The significant variation in quality of guest exchange for similar conditions and the challenge of reliability with the CS method is further emphasized by both the absence of trends from plots of two-factor influences with analyte grades and their lack of statistical significance (Section S12). These trends are severely influenced by the lack of guest exchange for 4°C and 48 h experiments (Table 2 ▸), because they provide no comparable data on guest quality. In relation to temperature, this can be explained by the lack of kinetic energy for diffusion of guest molecules, whereas the relationship with exchange duration appears to be more complex and requires further investigation.

**Figure 10:**
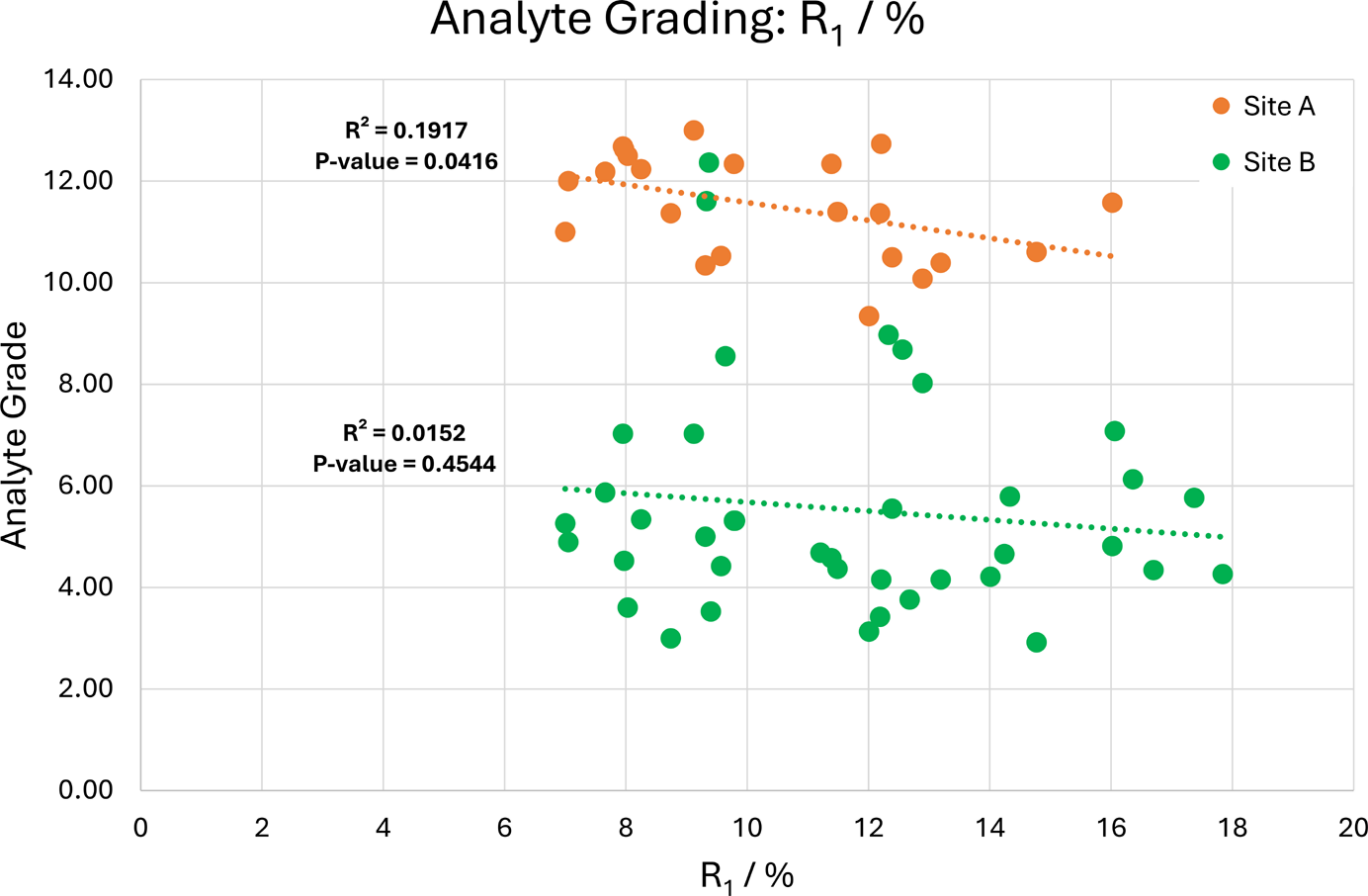
Scatterplot of analyte-grade relationship to *R*_1_ (%).

Analyte grades for BBA-8,12-OMe guests were also compared with traditional crystallographic metrics, including *R*_1_, *w*R_2_, *I*/σ *etc*. The aim of this investigation was to identify a statistical metric which may act as an indicator for higher quality guest exchange. The comparative plot for *R*_1_ is shown in Fig. 10 ▸ and corresponding graphs can be found in Section S13.

The scatterplot features a weak negative correlation between *R*_1_ and the quality of the secondary exchange site. This is further corroborated by a similar trend for *R*_int_ (Section S13) and highlights the importance of adopting this style of grading system that can empower researchers to report geometrically accurate structures which possess higher *R* values. This analysis complements the work of Spek (2018 ▸), who first explored the relationship between structural validity and traditional crystallographic metrics for CS data. In agreement with Spek’s analysis, many crystallographic metrics do not possess statistically significant trends with respect to analyte grades. This is primarily caused by the minor contribution of the guest molecule to the diffraction pattern, and the subsequent influence they have on refinement statistics can be negligible compared with the dominant host framework which contains heavier elements.

#### 3.3.1. Application of optimal conditions

Although only limited insights could be made into guest quality trends, it was still possible to identify the optimal conditions for exchange of BBA-8,12-OMe into the CS host. This was 50°C|10 mg ml^−1^|96 h because of its consistent success across all replicates and the high-quality of multiple guest models. Contrastingly, one of the worst combinations of conditions for guest exchange was found to be 4°C|10 mg ml^−1^|96 h which had inconsistent success but did enable characterization of low-quality guest sites. This once again reiterates the influential role temperature has on this specific system.

**Figure 11:**
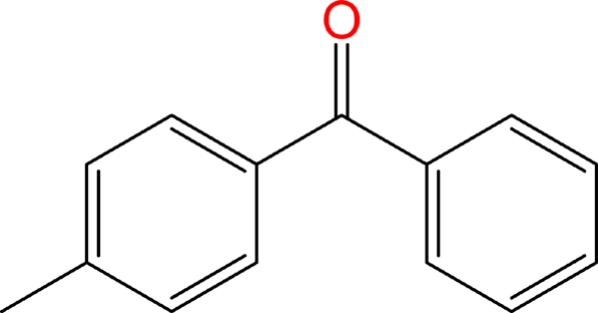
Molecular structure of 4-methyl­benzo­phenone.

Although previous research indicates that optimal exchange conditions can be highly dependent on the chosen guest molecule, as a validation of these findings, the ‘best’ and ‘worst’ conditions were applied to a structurally similar, small organic analyte. This enables the application of the quantitative structural grading system to assess for similar experimental trends in other guests, even if they are not fully optimized. Previous experiments conducted in our laboratory indicated variable soaking behaviour for 4-methyl­benzo­phenone (Fig. 11 ▸) and it was therefore chosen for the application of this approach.

Full crystallographic details of 4-methyl­benzo­phenone structures, including application of crystallographic restraints/constraints and structural grading scores, can be found in Section S14.

4-methyl­benzo­phenone displays similar experimental trends to BBA-8,12-OMe: higher temperature soaks exhibited more consistency for guest exchange while lower temperature experiments were unable to resolve any guests. Interestingly, the experiments conducted at 4°C did exhibit some clear signs of guest uptake even though no guests could be modelled, this is discussed in Section S15.

As observed with BBA-8,12-OMe, efficient uptake of 4-methyl­benzo­phenone into the host framework resulted in a reduction of crystallographic symmetry (*C*2/*c* to *P*1). This feature has been identified for a number of highly compatible analytes, such as the anthelminthic drug α-santonin (Hoshino *et al.*, 2016 ▸). These changes, in comparison with traditional crystallographic metrics, offer some of the most reliable indications that guest exchange has occurred with high efficiency and, consequently, that guests can be modelled with geometric accuracy and fewer restraints/constraints.

In the case of 4-methyl­benzo­phenone, two unit cells are observed for successful exchange: *P*1 ≃ 8000 Å^3^ and *P*1 ≃ 16000 Å^3^. The *P*1 (8k) form possesses two unique exchange sites with structures exhibiting an average grading score of 4.59, which represents a higher quality exchange than most experiments conducted for BBA-8,12-OMe. The *P*1 (16k) form possesses ten unique exchange sites with an average score of 4.71. This structure displayed greater variation of guest exchange quality, with the best-defined sites achieving scores of 1.50 and 1.69, while the weakest sites scored 10.00 and 10.13. Comparison of scores for future studies will depend largely on the aims of a study; however, it is expected that achieving the single highest-quality site may be most desirable. Further analysis of the crystallographic results can be found in Section S15.

Although these experimental conditions may well not be the ‘optimal’ exchange parameters for 4-methyl­benzo­phenone, they do highlight that experimental trends for structurally similar molecules can provide a useful starting point for investigation. These findings will therefore speed up the iterative experimental procedure and will be particularly useful for studies examining multiple types of CS host simultaneously.

## 4. Conclusions and future work

Application of statistical DoE principles to CS experiments has explored three responses, each highlighting the significant variability in the CS method and the importance of optimizing experimental conditions to achieve high-quality molecular structure characterization.

We have shown for BBA-8,12-OMe that exchange temperature is the main effect for successful exchange and ordering of guests within the CS host. These exchanges are observed in three different crystallographic forms which enabled preliminary categorization of exchange efficiency and geometric accuracy. Here it was shown that, while exchange temperature had the most evident influence, analyte concentration also impacted the crystallographic form adopted.

To further analyse these results, a novel grading system integrated into the crystallographic refinement procedure has been developed. This system provides a simple metric for representation of data quality to both specialist and non-specialist researchers who may utilize the CS method in their work. This represents a significant improvement from previous CS practices, where guest occupancy was used as an isolated, and often ineffective, quality indicator.

Complementary analysis was obtained for a structurally similar, small organic molecule and demonstrated that optimal conditions can be analyte specific, but still have useful applications for minimizing iterative experimentation on previously unexplored materials.

Further statistical studies which implement DoE principles are currently being undertaken to investigate the influence of host crystal size. This work has notable possible applications to three-dimensional electron diffraction, where nanocrystals have the potential to revolutionize the approach to CS experiments.

Future developments of the structural grading system will introduce scores for more restraints/constraints commonly available to researchers and apply more structural descriptors, such as analysis of local electron density maps for guest-exchange sites. Development of an automated script for calculating molecule grades will be undertaken following community feedback on relative scores and other features. Alongside this, future iterations will also implement database derived geometric libraries, such as the CCDC *Mogul* software, which will eliminate the use of conventional SCXRD data as reference material (Bruno *et al.*, 2004 ▸; Cottrell *et al.*, 2012 ▸). Wider adoption of the system and accumulation of data into a comprehensive library may allow the quantitative grading system to be applied more broadly when informing researchers, *i.e.* whether their data is suitable for accurate, detailed conformational analysis or provides reliable molecular connectivity only.

## 5. Related literature

The following references are cited in the supporting information: Dolomanov *et al.* (2009 ▸); Groom *et al.* (2016 ▸); Kutzke *et al.* (1996 ▸); Sheldrick (2015 ▸); Spek (2003 ▸).

## Supplementary Material

Crystal structure: contains datablock(s) global, BBA_8_12_OMe, 4_1_24a, 4_1_24b, 4_1_24c, 4_1_48a, 4_1_48b, 4_1_48c, 4_1_96a, 4_1_96b, 4_1_96c, 4_5_48a, 4_5_48b, 4_5_48c, 4_10_24a, 4_10_24b, 4_10_24c, 4_10_96a, 4_10_96b, 4_10_96c, 25_1_24a, 25_1_24b, 25_1_24c, 25_1_48a, 25_1_48b, 25_1_48c, 25_1_96a, 25_1_96b, 25_1_96c, 25_5_24a, 25_5_24b, 25_5_24c, 25_5_96a, 25_5_96b, 25_5_96c, 25_10_24a, 25_10_24b, 25_10_24c, 25_10_48a, 25_10_48b, 25_10_48c, 50_1_24a, 50_1_24b, 50_1_24c, 50_1_96a, 50_1_96b, 50_1_96c, 50_5_48a, 50_5_48b, 50_5_48c, 50_5_96a, 50_5_96b, 50_5_96c, 50_10_24a, 50_10_24b, 50_10_24c, 50_10_48a, 50_10_48b, 50_10_48c, 50_10_96a, 50_10_96b, 50_10_96c, 4_mbp_4_10_96a, 4_mbp_4_10_96b, 4_mbp_4_10_96c, 4_mbp_50_10_96a, 4_mbp_50_10_96b, 4_mbp_50_10_96c. DOI: 10.1107/S2052252524004871/lt5068sup1.cif

Supporting information file. DOI: 10.1107/S2052252524004871/lt5068_4_mbp_50_10_96b_sup2.cml

Supporting information file. DOI: 10.1107/S2052252524004871/lt5068_BBA_8_12_OMe_from_opsin_sup3.cml

Supporting information file. DOI: 10.1107/S2052252524004871/lt5068_BBA_8_12_OMe_sup4.cml

Supporting figures and tables. DOI: 10.1107/S2052252524004871/lt5068sup5.pdf

CCDC references: 2341226, 2342842, 2342843, 2342844, 2342845, 2342846, 2342847, 2342848, 2342849, 2342850, 2342851, 2342852, 2342853, 2342854, 2342855, 2342856, 2342857, 2342858, 2342859, 2342860, 2342861, 2342862, 2342863, 2342864, 2342865, 2342866, 2342867, 2342868, 2342869, 2342870, 2342871, 2342872, 2342873, 2342874, 2342875, 2342876, 2342877, 2342878, 2342879, 2342880, 2342881, 2342882, 2342883, 2342884, 2342885, 2342886, 2342887, 2342888, 2342889, 2342890, 2342891, 2342892, 2342893, 2342894, 2342895, 2342896, 2342897, 2342898, 2342899, 2342900, 2342901, 2343727, 2343728, 2343729, 2343730, 2343731, 2343732,

## Figures and Tables

**Figure 9:**
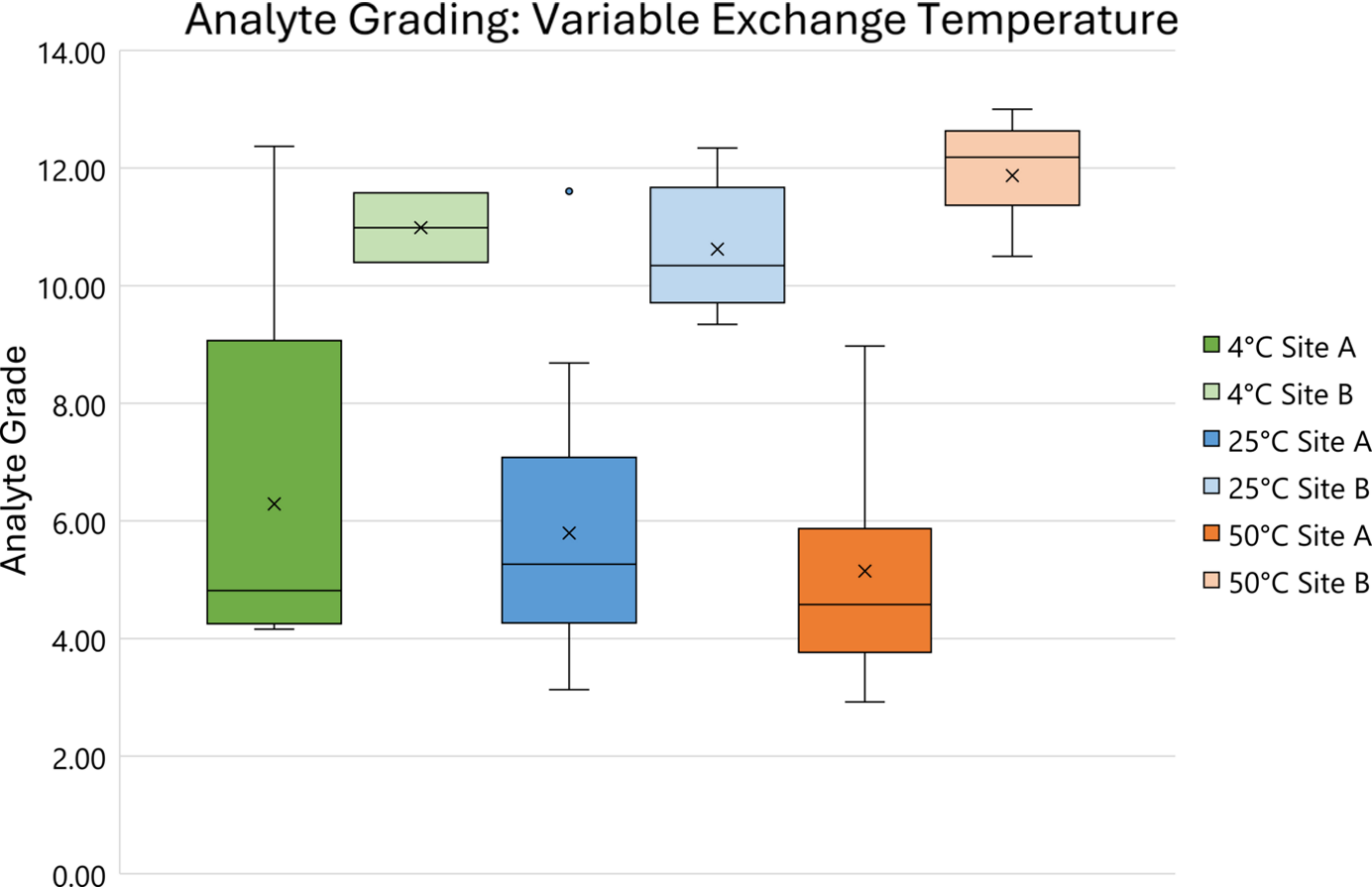
Box and whisker plot of analyte-grade relationship to variable exchange temperature with components separated into crystallographic space groups.

**Table 1:** Factors and factor levels selected for defining DoE runs

Exchange temperature (°C)	Analyte concentration (mg ml^−1^)	Exchange duration (h)
4	1	24
25	5	48
50	10	96

**Table 2:** Most (top) and least (bottom) consistent exchange of BBA-8,12-OMe with respect to two factor influences

Factor A	Factor B	Success rate (%)
5 mg ml^−1^	96 h	100.00
25°C	96 h	100.00
50°C	24 h	100.00
50°C	96 h	100.00
50°C	1 mg ml^−1^	100.00

4°C	48 h	0.00
4°C	5 mg ml^−1^	0.00
4°C	10 mg ml^−1^	33.33
1 mg ml^−1^	48 h	33.33
5 mg ml^−1^	48 h	33.33

**Table 3:** Guest-exchange rates and exchange site occupancies for the three crystallographic forms

	*C*2/*c* (16k)	*P*2/*n*	*C*2/*c* (48k)
No. of guest sites	1	1–2	1–3
Ordered solvent	No	Yes	Yes
Site A occupancy (%)	18.6–29.5	45.6–84.2	45.5–85.8
Site B occupancy (%)	–	15.5–30.0	11.3–38.7
Site C occupancy (%)	–	–	21.9

## References

[bb1] Bruno, I. J., Cole, J. C., Kessler, M., Luo, J., Motherwell, W. D. S., Purkis, L. H., Smith, B. R., Taylor, R., Cooper, R. I., Harris, S. E. & Orpen, A. G. (2004). *J. Chem. Inf. Comput. Sci.* **44**, 2133–2144. 10.1021/ci049780b 15554684

[bb2] Carroll, R. C., Harrowven, D. C., Pearce, J. E. & Coles, S. J. (2023). *IUCrJ*, **10**, 497–508. 10.1107/S2052252523005146 PMC10324488 37409807

[bb3] Chen, H., Jin, C., Chen, X., Yu, J., Yan, D., Cheng, P., Zhang, Z., Zhao, L. & Chen, Y. (2022). *Chem. Eng. J.* **444**, 136624.

[bb4] Cottrell, S. J., Olsson, T. S. G., Taylor, R., Cole, J. C. & Liebeschuetz, J. W. (2012). *J. Chem. Inf. Model.* **52**, 956–962. 10.1021/ci200439d 22372622

[bb900] Dolomanov, O. V., Bourhis, L. J., Gildea, R. J., Howard, J. A. K. & Puschmann, H. (2009). *J. Appl. Cryst.* **42**, 339–341.

[bb5] Fisher, R. A. (1935). *The Design of Experiments*. Edinburgh: Oliver and Boyd.

[bb901] Groom, C. R., Bruno, I. J., Lightfoot, M. P. & Ward, S. C. (2016). *Acta Cryst.* B**72**, 171–179. 10.1107/S2052520616003954 PMC4822653 27048719

[bb6] Habib, F., Tocher, D. A. & Carmalt, C. J. (2022). *Mater. Today Proc.* **56**, 3766–3773.

[bb7] Hayes, L. M., Knapp, C. E., Nathoo, K. Y., Press, N. J., Tocher, D. A. & Carmalt, C. J. (2016). *Cryst. Growth Des.* **16**, 3465–3472.

[bb8] Hoshino, M., Khutia, A., Xing, H., Inokuma, Y. & Fujita, M. (2016). *IUCrJ*, **3**, 139–151. 10.1107/S2052252515024379 PMC4775162 27006777

[bb9] Inokuma, Y., Ukegawa, T., Hoshino, M. & Fujita, M. (2016). *Chem. Sci.* **7**, 3910–3913. 10.1039/c6sc00594b PMC6013795 30155035

[bb10] Inokuma, Y., Yoshioka, S., Ariyoshi, J., Arai, T., Hitora, Y., Takada, K., Matsunaga, S., Rissanen, K. & Fujita, M. (2013). *Nature*, **495**, 461–466. 10.1038/nature11990 23538828

[bb903] Kutzke, H., Al-Mansour, M. & Klapper, H. (1996). *J. Mol. Struct.* **374**, 129–135.

[bb11] Li, Y., Tang, S., Yusov, A., Rose, J., Borrfors, A. N., Hu, C. T. & Ward, M. D. (2019). *Nat. Commun.* **10**, 4477. 10.1038/s41467-019-12453-6 PMC6775153 31578331

[bb12] Lunn, R. D. J., Tocher, D. A., Sidebottom, P. J., Montgomery, M. G., Keates, A. C. & Carmalt, C. J. (2020). *Cryst. Growth Des.* **20**, 7238–7245. 10.1021/acs.cgd.1c00196 PMC8154245 34054355

[bb13] Metherall, J. P., Carroll, R. C., Coles, S. J., Hall, M. J. & Probert, M. R. (2023). *Chem. Soc. Rev.* **52**, 1995–2010. 10.1039/d2cs00697a 36857636

[bb14] Meurer, F., von Essen, C., Kühn, C., Puschmann, H. & Bodensteiner, M. (2022). *IUCrJ*, **9**, 349–354. 10.1107/S2052252522002147 PMC9067116 35546798

[bb100] Moghadam, P. Z., Li, A., Wiggin, S. B., Tao, A., Maloney, A. G. P., Wood, P. A., Ward, S. C. & Fairen-Jimenez, D. (2017). *Chem. Mater.* **29**, 2618–2625.

[bb16] Ning, G. H., Matsumura, K., Inokuma, Y. & Fujita, M. (2016). *Chem. Commun.* **52**, 7013–7015. 10.1039/c6cc03026b 27157794

[bb17] Rosenberger, L., von Essen, C., Khutia, A., Kühn, C., Urbahns, K., Georgi, K., Hartmann, R. W. & Badolo, L. (2020). *Drug Metab. Dispos.* **48**, 587–593. 10.1124/dmd.120.091140 32434832

[bb18] Sakurai, F., Khutia, A., Kikuchi, T. & Fujita, M. (2017). *Chem. A Eur. J.* **23**, 15035–15040. 10.1002/chem.201704176 28885761

[bb904] Sheldrick, G. M. (2015). *Acta Cryst.* C**71**, 3–8.

[bb906] Spek, A. L. (2003). *J. Appl. Cryst.* **36**, 7–13.

[bb19] Spek, A. L. (2018). *Inorg. Chim. Acta*, **470**, 232–237.

[bb20] Tyler, A. R., Ragbirsingh, R., McMonagle, C. J., Waddell, P. G., Heaps, S. E., Steed, J. W., Thaw, P., Hall, M. J. & Probert, M. R. (2020). *Chem*, **6**, 1755–1765. 10.1016/j.chempr.2020.04.009 PMC7357602 32685768

[bb21] Wada, N., Kageyama, K., Jung, Y., Mitsuhashi, T. & Fujita, M. (2021). *Org. Lett.* **23**, 9288–9291. 10.1021/acs.orglett.1c03660 34806896

[bb22] Wada, Y., Usov, P. M., Chan, B., Mukaida, M., Ohmori, K., Ando, Y., Fuwa, H., Ohtsu, H. & Kawano, M. (2024). *Nat. Commun.* **15**, 81. 10.1038/s41467-023-44401-w PMC10762011 38167264

[bb23] Waldhart, G. W., Mankad, N. P. & Santarsiero, B. D. (2016). *Org. Lett.* **18**, 6112–6115. 10.1021/acs.orglett.6b03119 PMC5502779 27934356

[bb24] Yuangyai, C. & Nembhard, H. B. (2010). *Emerging Nanotechnologies for Manufacturing*, edited by W. Ahmed, M. J. Jackson, pp. 207–234. William Andrew Publishing.

[bb25] Zigon, N., Duplan, V., Wada, N. & Fujita, M. (2021). *Angew. Chem. Int. Ed.* **60**, 25204–25222. 10.1002/anie.202106265 34109717

