# Supplementary material for: Statistical optimization of guest uptake in crystalline sponges: grading structural outcomes
Source: IUCrJ. 2024 Jun 12;11(Pt 4):578–86. doi: 10.1107/S2052252524004871 (PMC11220878; doi:10.1107/S2052252524004871)
Supplement: Supplementary file 5 [file m-11-00578-sup5.pdf]

IUCrJ

Volume 11 (2024)

Supporting information for article:

Statistical optimization of guest uptake in crystalline sponges: grading structural outcomes

Robert C. Carroll and Simon J. Coles

Supporting information

Electronic Supplementary Information for "**Statistical optimisation of guest uptake in crystalline sponges: grading structural outcomes**".

Robert C. Carroll*, Simon J. Coles*

School of Chemistry, University of Southampton, University Road, Southampton, Hampshire, SO17 1BJ, United Kingdom

Correspondence emails: R.C.Carroll@soton.ac.uk , S.J.Coles@soton.ac.uk

IMPORTANT: this document contains embedded data - to preserve data integrity, please ensure where possible that the IUCr Word tools (available from http://journals.iucr.org/services/docxtemplate/) are installed when editing this document.

Table of Contents

S1. Statistical Design of Experiments Generation…………………………………………………...3

S2. Guest Exchange Procedure……………………………………………………………………5

S3. Data Collection and Refinement Strategy……………………………………………………….6

S4. Analyte Structural Grading System…………………………………………………………...8

S5. BBA-8,12-OMe Crystallographic Tables, Diagrams, and Notes…………………………………12

S6. Guest Exchange Rates Full Tabulation…………………………………………………………74

S7. Crystallographic Analysis of P2/n and C2/c [48k] Forms………………………………………...79

S8. Crystallographic Form Occurrence……………………………………………………………..81

S9. Crystallographic Form Interaction Analysis……………………………………………………83

S10. BBA-8,12-OMe Analyte Grades Summary……………………………………………...……86

S11. Analyte Grading: Single Factor Plots…………………………………………………………88

S12. Analyte Grading: Two Factor Influences……………………………………………….......90

S13. Analyte Grading: Traditional Crystallographic Metrics………………………………………..103

S14. 4-Methylbenzophenone Crystallographic Tables, Diagrams, and Notes…………………...….110

S15.

4-Methylbenzophenone Analyte Grades and Discussion………………………………………117

S16. References……………………………………………………………………………………118

S1. Statistical Design of Experiments Generation

A multilevel factorial design was generated for consideration of the three main experimental factors (exchange temperature, analyte concentration, and exchange duration) at three values. This resulted in 27 unique experiments and a total of 81 total experiments with the inclusion of replicates. Following this, a factorial design selected according to D-optimality was generated with design points varying from 19 – 21. These designs explored the model terms A, B, C, AB, AC, BC (where A = exchange temperature, B = analyte concentration, and C = exchange duration). The statistical optimality metrics are shown in Table S1.

Table S1 Statistical optimality metrics determined for three potential experimental designs.

	Optimal Design (19)	Optimal Design (20)	Optimal Design (21)
**D-Optimality**	8.24×10^{14}	6.59×10^{15}	3.27×10^{16}
**A-Optimality**	7.07	5.97	4.85
**G-Optimality**	1.00	0.98	0.98
**V-Optimality**	1.00	0.95	0.90
**Maximum Leverage**	1.00	0.97	0.92

It was determined that an optimal design with 20 unique experiments (60 total experiments) represented the best compromise between statistical information and time constraints of the work. The 20 unique experiments are shown in Table S2.

Table S2 Unique experimental conditions explored in the statistical study.

Exchange Temperature / °C	Analyte Concentration / mg mL^{-1}	Exchange Duration / hrs
50	1	24
50	10	24
50	5	48
50	10	48
50	1	96
50	5	96
50	10	96
25	1	24
25	5	24
25	10	24
25	1	48
25	10	48
25	1	96
25	5	96
4	1	24
4	10	24
4	1	48
4	5	48
4	1	96
4	10	96

S2. Guest Exchange Procedure

Crystalline sponge host crystals ([{$(ZnI_2)_3(tpt)_2 \cdot x(C_6H_{12})$}$_n$]) were prepared following the reported procedures and supplied by Merck Crystal-Do. (Hoshino *et al.*, 2016) Guest exchanges were left to evaporate to dryness and SCXRD data was collected on the same day as evaporation finished. Summarised details of exchange conditions are shown below in Table S3.

Table S3 Practical guidelines for evaporation with varied exchange temperatures and exchange durations.

Exchange Temperature / °C	Exchange Time / hrs	Host Solvent Volume / µL	Needle Diameter / mm
4	24	8	0.8
4	48	12	0.8
4	96	10	0.4
25	24	18	0.8
25	48	12	0.5
25	96	20	0.4
50	24	50	0.8
50	48	50	0.5
50	96	50	0.4

To account for day-to-day environmental or experimental variables, replicates for unique conditions were conducted on different days. For CS guest exchange, all analyte solutions were added as 1µL in dichloromethane (DCM), regardless of the concentration, to minimise different influences on the host crystal or 'leaching' of guests from host cavities.

S3. Data Collection and Refinement Strategy

Single crystal X-ray diffraction experiments were carried out on Rigaku 007HF diffractometer using Cu-K_{α} radiation (λ = 1.54184 Å). Equipped with Varimax confocal mirrors, UG2 goniometer, and HyPix 6000HE / HyPix Arc-100 detectors. Data was collected to 0.83Å resolution for consistency across all samples. Strategies were calculated based on 15 I/σ for the weakest reflections, however this was not always possible to achieve due to limitations in diffraction, and in these cases data collection was limited to a maximum of 3 hours.

CrysAlis^{Pro} software was used for calculation of experiment strategy and data reduction (data integration, empirical and numerical absorption corrections, and scaling). Structures possessing significant twinning (>20% component) were recollected to enable closer comparison of crystallographic metrics.

All structures were modelled with Olex2 v1.5, solved with SHELXT v2014/5, and refined with SHELXL v2016/6. (Dolomanov *et al.*, 2009; Sheldrick, 2015*b*,*a*) Attempts were made to model all non-hydrogen atoms anisotropically. However, low occupancy guests / solvents and overlapping sites prevented stable refinement for some. In these cases, rather than applying severe thermal restraints, the molecule was modelled isotropically with restrained thermal parameters (U_{iso} = 0.08). Hydrogen atoms were fixed with a riding model.

For determination of guest molecule occupancies, non-hydrogen atoms were refined as isotropic with a molecule free-variable (FVAR) and restrained thermal parameters (U_{iso} = 0.08). The guest occupancies were then fixed before anisotropic refinement to prevent expansion of thermal ellipsoids and erroneous assignment of residual electron density within the porous framework. Restraints / constraints were required for most guest molecules and are detailed for each dataset. Solvent molecules were found in the difference map and refined as restrained bodies (DFIX, DANG, SIMU, RIGU, and SADI). The considerable thermal motion of solvent within the pore results in some of the averaged structures possessing geometrically unfavourable distortions.

During finalisation of crystallographic structure refinements, some high-intensity reflections were omitted when they possessed an [Error/esd] > 10. The framework numbering scheme illustrated below in Figure S1 was applied to each structure to assist rapid identification of host-guest interactions within the structures.

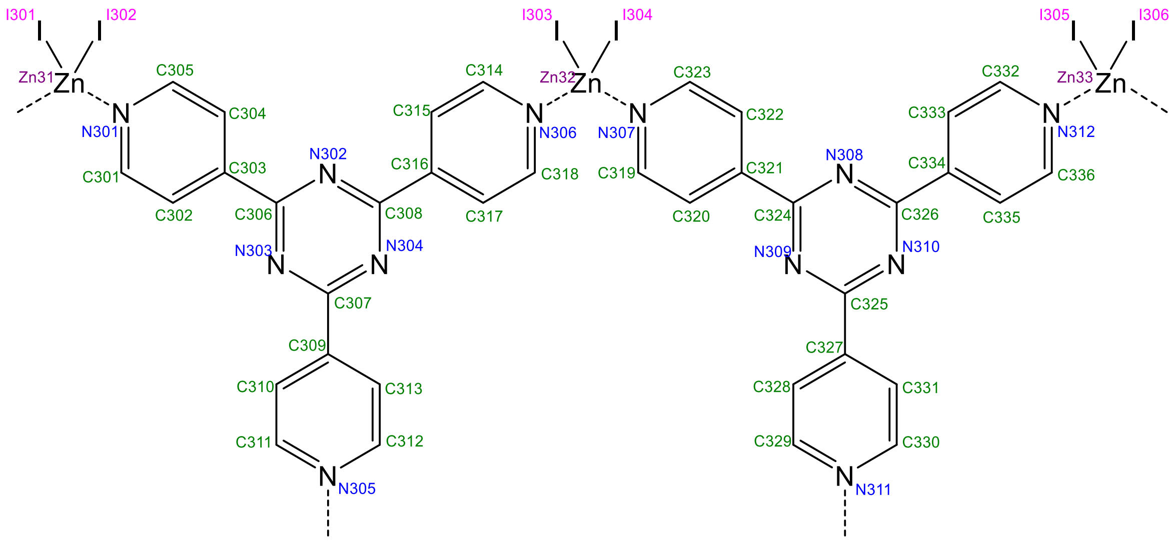

Figure S1 Crystalline sponge host framework numbering scheme.

S4. Analyte Structural Grading System

To determine analyte grades, the following restraints, constraints, guest occupancies, and thermal parameters in Tables S4, S5, and S6 were assigned scores. The scoring system is relative, not absolute, with scores for individual restraints / constraints adjusted in relation to a collection of internal CS datasets until there was satisfactory distinction. This was undertaken with input from experienced crystallographers but will require community input to further refine scoring schemes. These values were then used in Equation S1.

Table S4 Progressive scores for geometric restraints and constraints.

Restraint / Constraint	ESD	Points
SADI (1,2)	0.02	1.0
SADI (1,3)	0.04	1.0
DFIX	0.02	2.0
DANG	0.04	2.0
SADI (1,4)	0.04	2.0
FLAT (Ring)	0.1	3.0
DFIX	0.01	3.0
DANG	0.02	3.0
FLAT (Arm)	0.1	3.0
DANG	0.01	4.0
AFIX 66	-	9.0

Table S5 Progressive scores for guest exchange site occupancy.

Guest Occupancy / %	Points
90+	0.0
80 – 89	0.5
70 – 79	1.0
60 – 69	1.5
50 – 59	2.0
40 – 49	2.5
30 – 39	3.0
20 – 29	3.5
10 – 19	4.0
0 – 9	4.5

Guests modelled on crystallographic symmetry sites which intrinsically limit occupancy are normalised to 100%, i.e., a guest modelled with 20% occupancy on a 2-fold symmetry axis is given the equivalent points for 40% occupancy.

Table S6 Progressive scoring system for guest thermal parameters and restraints.

U_{eq} / Thermal Restraint	Points
0.070 – 0.089	0.0
0.090 – 0.119	0.5
0.120+	1.0
Isotropic	2.0
SIMU	0.0
RIGU	0.5

Equation S1 Analyte grading system equation

$$Grade = \left(\frac{Res + Con}{N_B}\right) + \left(\frac{Th_R \times N_A}{N_A}\right) + Th_P + Oc$$

Where: *Res* = points for restraints, *Con* = points for constraints, N_B = number of non-hydrogen bonds, and Th_R = points for thermal restraints, N_A = number of non-hydrogen atoms Th_R has been applied to, and *Oc* = points for occupancy. To determine when a restraint or constraint had adequately modelled the relevant guest geometry, the following tolerances from Table S7 were used.

Table S7 Geometric tolerances used for determination of suitable bond distances / angles.

Geometric Feature	Tolerance
1,2 – Distance	±0.02 Å
1,3 – Distance	±0.02 Å
Bond Angle	±2.00 °
Mean Plane (MPLN) Flatness	±0.02 Å

To showcase the integrated nature of the crystallographic restraints and analyte grades, a fully worked example is presented from 50_1_24b. The numbered structure of BBA-8,12-OMe is illustrated alongside the restraint and constraint representations in Figure S2.

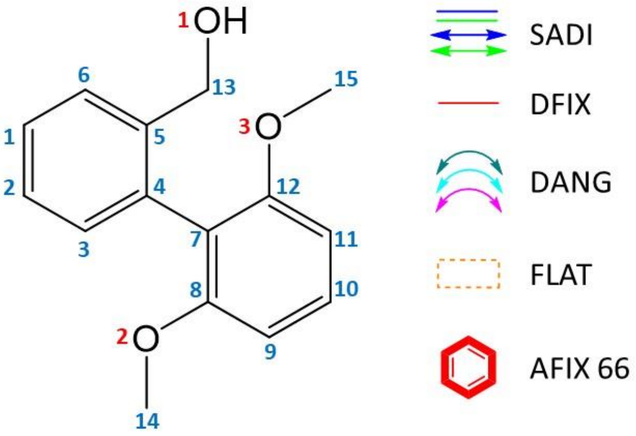

Figure S2 BBA-8,12-OMe structure and crystallographic restraint representations

From a practical perspective, any bond shown in black has had no geometric restraints or constraints applied, while any colouration or emboldening indicates application of crystallographic restraints or constraints. The finalised diagram for 50_1_25b site A is illustrated with a text-based version of the restraints in Figure S3.

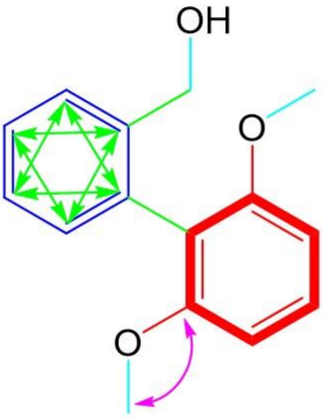


```
SADI C1A C2A C2A C3A C3A C4A C4A C5A C5A C6A C6A C1A
SADI C1A C3A C2A C4A C3A C5A C4A C6A C5A C1A C6A C2A
AFIX 66 C7A C8A C9A C10A C11A C12A
SADI C4A C7A C5A C13A
SADI O1A C13A O2A C14A O3A C15A
DFIX 1.37 O2A C8A O3A C12A
DANG 2.35 C8A C14A
SIMU C1A C2A C3A C4A C5A C6A C7A C8A C9A C10A C11A C12A =
 C13A C14A C15A O1A O2A O3A
RIGU C1A C2A C3A C4A C5A C6A C7A C8A C9A C10A C11A C12A =
 C13A C14A C15A O1A O2A O3A
```

Figure S3 50_1_25b site A restraints diagram and text-based equivalent

The following scores can be assigned to each molecule component:

C1A – C6A: $(6 \times SADI\ 1{,}2) + (6 \times SADI\ 1{,}3) = (6 \times 1.0) + (6 \times 1.0) = 12$

C7A – C12A: $(6 \times AFIX\ 66) = (6 \times 9.0) = 54$

C4A – C7A: $(1 \times SADI\ 1{,}2) = (1 \times 1.0) = 1$

C5A – C13A – O1A: $(2 \times SADI\ 1{,}2) = (2 \times 1.0) = 2$

C8A – O2A – C14A: $(1 \times DFIX) + (1 \times SADI\ 1{,}2) + (1 \times DANG) = (1 \times 2.0) + (1 \times 1.0) + (1 \times 2.0) = 5$

C12A – O3A – C15A: $(1 \times DFIX) + (1 \times SADI\ 1{,}2) = (1 \times 2.0) + (1 \times 1.0) = 3$

Thermal Restraints: $SIMU + RIGU = \frac{(0.0+0.5)\times 18}{18} = 0.5$

Average U$_{eq}$: $0.095 = 0.5$

Guest Occupancy: $27.1\% = 3.5$

These values can then be put into Equation S1 to provide the following:

$$Grade = \left(\frac{(12 + 1 + 2 + 5 + 3) + 54}{19}\right) + \left(\frac{(0.0 + 0.5) \times 18}{18}\right) + 0.5 + 3.5 = 8.55$$

S5. BBA-8,12-OMe Crystallographic Tables, Diagrams, and Notes

All thermal ellipsoid plots for CS structures are shown with residual electron density maps at 0.7 electrons per cubic Angstrom (e/Å^3).

Table S8 BBA-8,12-OMe traditional SCXRD crystallographic table

CCDC Deposition Number	2341226
Empirical formula	$C_{15}H_{16}O_3$
Formula weight	244.28
Temperature/K	100.00(10)
Crystal system	Monoclinic
Space group	I2/a
a/Å	23.913(2)
b/Å	7.6350(7)
c/Å	27.048(2)
α = γ /°	90
β/°	93.244(7)
Volume/Å^3	4930.4(8)
Z	16
ρ_{calc}g/cm^3	1.316
μ/mm^{-1}	0.737
F(000)	2080.0
Crystal size/mm^3	0.13 × 0.02 × 0.02
Radiation	Cu Kα (λ = 1.54184)
2Θ range for data collection/°	6.456 to 139.378
Index ranges	-28 ≤ h ≤ 28, -9 ≤ k ≤ 7, -32 ≤ l ≤ 32
Reflections collected	7946
Independent reflections	7946 [R_{int} = 0.2224, R_{sigma} = 0.0774]
Data/restraints/parameters	7946/0/454
Goodness-of-fit on F^2	1.070
Final R indexes [I>=2σ (I)]	R_1 = 0.0795, wR_2 = 0.2124

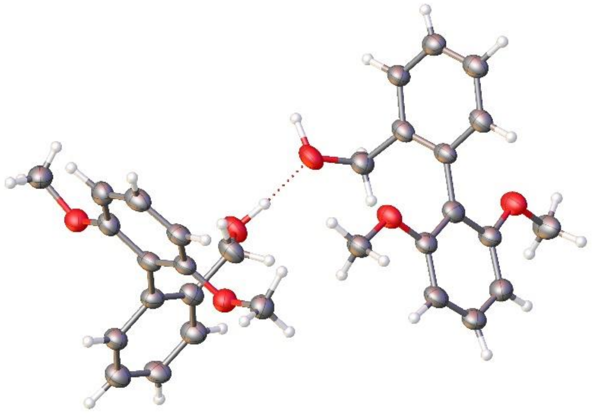

Figure S4 Asymmetric unit of BBA-8,12-OMe pure crystal with thermal ellipsoids displayed at 50% probability.

Table S9 BBA-8,12-OMe 4_1_24a crystallographic table

CCDC Deposition Number	2342863
Empirical formula	$C_{115.57}H_{79.8}I_{18}N_{36}O_{1.51}Zn_9$
Formula weight	4869.59
Temperature/K	100(2)
Crystal system	monoclinic
Space group	C2/c
a/Å	78.549(10)
b/Å	14.9682(8)
c/Å	41.688(3)
α = γ /°	90
β/°	90
Volume/Å^3	48312(8)
Z	8
ρ_{calc}g/cm^3	1.339
μ/mm^{-1}	19.313
F(000)	18091
Crystal size/mm^3	0.26 × 0.23 × 0.11
Radiation	Cu Kα (λ = 1.54184)
2Θ range for data collection/°	4.566 to 136.502
Index ranges	-93 ≤ h ≤ 94, -17 ≤ k ≤ 14, -50 ≤ l ≤ 41
Reflections collected	146635
Independent reflections	43775 [R_{int} = 0.0928, R_{sigma} = 0.0478]
Data/restraints/parameters	43775/958/1877
Goodness-of-fit on F^2	1.035
Final R indexes [I>=2σ (I)]	R_1 = 0.1738, wR_2 = 0.4738
Largest diff. peak/hole / e Å^{-3}	1.57/-1.11

SQUEEZE analysis indicated two voids with volumes of 684 and 2011Å^3 and electron counts of 156 and 382 respectively. This is equivalent to 3.25 and 7.96 cyclohexane molecules, or 1.20 and 2.94 BBA-8,12-OMe molecules.

Manual weighting scheme applied.

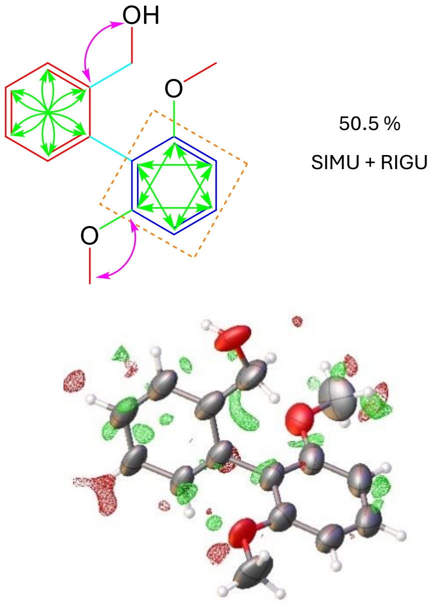

Figure S5 Geometric restraints and thermal ellipsoid (50% probability) for 4_1_24a Site A

Table S10 BBA-8,12-OMe 4_1_24b crystallographic table

CCDC Deposition Number	2342864
Empirical formula	$C_{46.36}H_{44.73}I_6N_{12}Zn_3$
Formula weight	1727.54
Temperature/K	100(2)
Crystal system	monoclinic
Space group	C2/c
a/Å	34.3474(9)
b/Å	15.1342(3)
c/Å	29.5974(9)
α = γ /°	90
β/°	90
Volume/Å^3	15131.6(7)
Z	8
ρ_{calc}g/cm^3	1.517
μ/mm^{-1}	20.584
F(000)	6519
Crystal size/mm^3	0.22 × 0.09 × 0.05
Radiation	Cu Kα (λ = 1.54184)
2Θ range for data collection/°	5.232 to 136.482
Index ranges	-41 ≤ h ≤ 38, -18 ≤ k ≤ 9, -35 ≤ l ≤ 34
Reflections collected	48974
Independent reflections	13719 [R_{int} = 0.0359, R_{sigma} = 0.0360]
Data/restraints/parameters	13719/586/910
Goodness-of-fit on F^2	1.061
Final R indexes [I>=2σ (I)]	R_1 = 0.0641, wR_2 = 0.1991
Largest diff. peak/hole / e Å^{-3}	1.38/-0.49
SQUEEZE analysis indicated one void with a volume of 73Å^3 and electron count of 15. This is equivalent to 0.31 cyclohexane molecules or 0.12 BBA-8,12-OMe molecules.	

Table S11 BBA-8,12-OMe 4_1_24c crystallographic table

CCDC Deposition Number	2342865
Empirical formula	$C_{127.68}H_{101.08}I_{18}N_{36}O_{2.2}Zn_9$
Formula weight	5047.47
Temperature/K	100.00(10)
Crystal system	monoclinic
Space group	C2/c
a/Å	78.299(4)
b/Å	14.9565(3)
c/Å	41.6827(16)
α = γ /°	90
β/°	90
Volume/Å^3	48083(3)
Z	8
ρ_{calc}g/cm^3	1.395
μ/mm^{-1}	19.427
F(000)	18886
Crystal size/mm^3	0.16 × 0.14 × 0.09
Radiation	Cu Kα (λ = 1.54184)
2Θ range for data collection/°	4.304 to 136.502
Index ranges	-94 ≤ h ≤ 93, -11 ≤ k ≤ 18, -50 ≤ l ≤ 49
Reflections collected	151389
Independent reflections	43690 [R_{int} = 0.0784, R_{sigma} = 0.0576]
Data/restraints/parameters	43690/1144/2145
Goodness-of-fit on F^2	1.042
Final R indexes [I>=2σ (I)]	R_1 = 0.1602, wR_2 = 0.4182
Largest diff. peak/hole / e Å^{-3}	1.72/-1.86
SQUEEZE analysis indicated three voids with volumes of 227, 84, and 1344Å^3 and electron counts of 54, 22, and 304 respectively. This is equivalent to 1.13, 0.46, and 6.33 cyclohexane molecules, or 0.18, 0.17, and 2.34 BBA-8,12-OMe molecules	

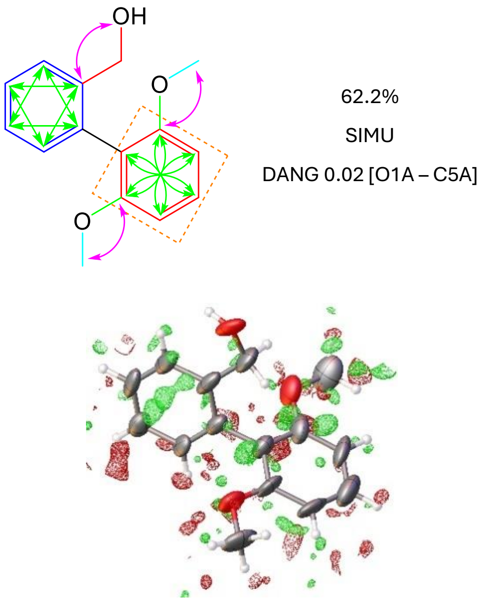

Figure S6 Geometric restraints and thermal ellipsoid (50% probability) for 4_1_24c Site A

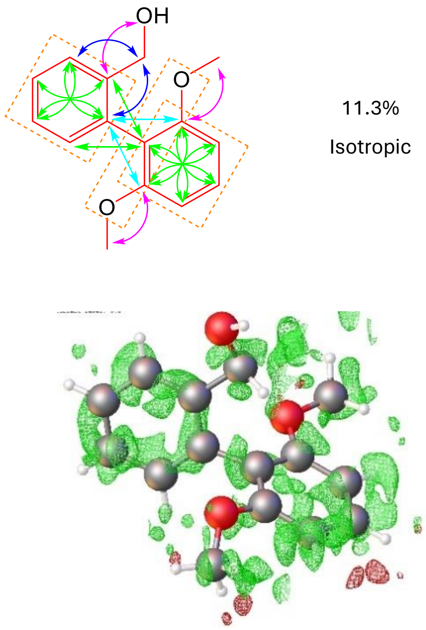

Figure S7 Geometric restraints and thermal ellipsoid (50% probability) for 4_1_24c Site B

Table S12 BBA-8,12-OMe 4_1_48a crystallographic table

CCDC Deposition Number	2342866
Empirical formula	$C_{36}H_{24}I_6N_{12}Zn_3$
Formula weight	1582.18
Temperature/K	100(2)
Crystal system	monoclinic
Space group	C2/c
a/Å	34.4506(11)
b/Å	15.0594(4)
c/Å	30.1018(14)
α = γ /°	90
β/°	90
Volume/Å^3	15338.6(10)
Z	8
ρ_{calc}g/cm^3	1.37
μ/mm^{-1}	20.257
F(000)	5856
Crystal size/mm^3	0.28 × 0.14 × 0.06
Radiation	Cu Kα (λ = 1.54184)
2Θ range for data collection/°	5.224 to 136.496
Index ranges	-41 ≤ h ≤ 41, -18 ≤ k ≤ 11, -36 ≤ l ≤ 32
Reflections collected	40957
Independent reflections	13694 [R_{int} = 0.0374, R_{sigma} = 0.0299]
Data/restraints/parameters	13694/21/622
Goodness-of-fit on F^2	1.065
Final R indexes [I>=2σ (I)]	R_1 = 0.0588, wR_2 = 0.1837
Largest diff. peak/hole / e Å^{-3}	0.69/-0.82
No guests or solvent were identified from the electron density map, SQUEEZE analysis indicated one void with a volume of 883Å^3 and electron count of 232. This is equivalent to 4.83 cyclohexane molecules or 1.78 BBA-8,12-OMe molecules.	

Table S13 BBA-8,12-OMe 4_1_48b crystallographic table

CCDC Deposition Number	2342867
Empirical formula	$C_{36}H_{24}I_6N_{12}Zn_3$
Formula weight	1582.18
Temperature/K	100(2)
Crystal system	monoclinic
Space group	C2/c
a/Å	34.1537(11)
b/Å	15.1026(3)
c/Å	29.0899(12)
α = γ /°	90
β/°	90
Volume/Å^3	14797.2(8)
Z	8
ρ_{calc}g/cm^3	1.42
μ/mm^{-1}	20.998
F(000)	5856
Crystal size/mm^3	0.13 × 0.08 × 0.03
Radiation	Cu Kα (λ = 1.54184)
2Θ range for data collection/°	5.248 to 136.502
Index ranges	-41 ≤ h ≤ 36, -18 ≤ k ≤ 9, -31 ≤ l ≤ 35
Reflections collected	47702
Independent reflections	13452 [R_{int} = 0.0504, R_{sigma} = 0.0490]
Data/restraints/parameters	13452/22/606
Goodness-of-fit on F^2	0.986
Final R indexes [I>=2σ (I)]	R_1 = 0.0567, wR_2 = 0.1604
Largest diff. peak/hole / e Å^{-3}	0.87/-0.45
No guests or solvent were identified from the electron density map, SQUEEZE analysis indicated one void with a volume of 820Å^3 and electron count of 180. This is equivalent to 3.75 cyclohexane molecules or 1.38 BBA-8,12-OMe molecules.	

Table S14 BBA-8,12-OMe 4_1_48c crystallographic table

CCDC Deposition Number	2342868
Empirical formula	$C_{36}H_{24}I_6N_{12}Zn_3$
Formula weight	1582.18
Temperature/K	100(2)
Crystal system	monoclinic
Space group	C2/c
a/Å	34.4353(8)
b/Å	15.0604(3)
c/Å	30.1558(14)
α = γ /°	90
β/°	90
Volume/Å^3	15361.6(9)
Z	8
ρ_{calc}g/cm^3	1.368
μ/mm^{-1}	20.227
F(000)	5856
Crystal size/mm^3	0.15 × 0.13 × 0.07
Radiation	Cu Kα (λ = 1.54184)
2Θ range for data collection/°	5.226 to 136.478
Index ranges	-36 ≤ h ≤ 41, -12 ≤ k ≤ 18, -36 ≤ l ≤ 36
Reflections collected	54183
Independent reflections	13987 [R_{int} = 0.0358, R_{sigma} = 0.0294]
Data/restraints/parameters	13987/22/616
Goodness-of-fit on F^2	1.071
Final R indexes [I>=2σ (I)]	R_1 = 0.0502, wR_2 = 0.1526
Largest diff. peak/hole / e Å^{-3}	1.05/-0.75
No guests or solvent were identified from the electron density map, SQUEEZE analysis indicated one void with a volume of 896Å^3 and electron count of 227. This is equivalent to 4.73 cyclohexane molecules or 1.75 BBA-8,12-OMe molecules.	

Table S15 BBA-8,12-OMe 4_1_96a crystallographic table

CCDC Deposition Number	2342869
Empirical formula	$C_{117.5}H_{82.13}I_{18}N_{36}O_{1.9}Zn_9$
Formula weight	4901.17
Temperature/K	100(2)
Crystal system	monoclinic
Space group	C2/c
a/Å	78.3675(18)
b/Å	14.9505(2)
c/Å	41.6842(7)
α = γ /°	90
β/°	90
Volume/Å^3	48118.2(15)
Z	8
ρ_{calc}g/cm^3	1.353
μ/mm^{-1}	19.396
F(000)	18226
Crystal size/mm^3	0.17 × 0.12 × 0.06
Radiation	Cu Kα (λ = 1.54184)
2Θ range for data collection/°	4.304 to 136.5
Index ranges	-94 ≤ h ≤ 94, -18 ≤ k ≤ 17, -49 ≤ l ≤ 50
Reflections collected	148464
Independent reflections	43548 [R_{int} = 0.0952, R_{sigma} = 0.0724]
Data/restraints/parameters	43548/1000/1906
Goodness-of-fit on F^2	1.049
Final R indexes [I>=2σ (I)]	R_1 = 0.1670, wR_2 = 0.4217
Largest diff. peak/hole / e Å^{-3}	1.64/-1.75

SQUEEZE analysis indicated two voids with volumes of 676 and 1988Å^3 and electron counts of 207 and 516 respectively. This is equivalent to 4.31 and 10.75 cyclohexane molecules, or 1.59 and 3.97 BBA-8,12-OMe molecules.

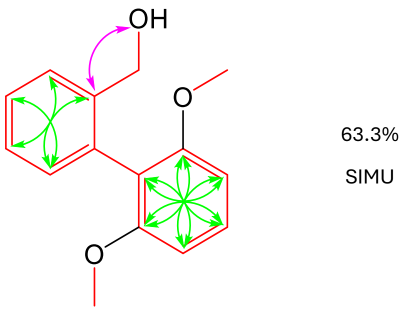

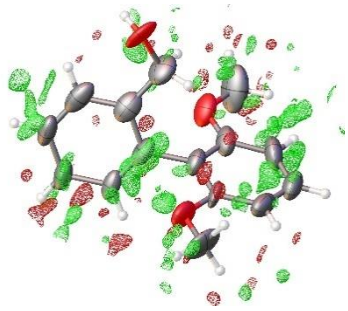

Figure S8 Geometric restraints and thermal ellipsoid (50% probability) for 4_1_96a Site A

Table S16 BBA-8,12-OMe 4_1_96b crystallographic table

CCDC Deposition Number	2342870
Empirical formula	$C_{37.73}H_{27.46}I_6N_{12}Zn_3$
Formula weight	1606.46
Temperature/K	100.00(10)
Crystal system	monoclinic
Space group	C2/c
a/Å	34.4432(11)
b/Å	15.0627(4)
c/Å	30.2136(14)
α = γ /°	90
β/°	90
Volume/Å^3	15387.3(10)
Z	8
ρ_{calc}g/cm^3	1.387
μ/mm^{-1}	20.201
F(000)	5967
Crystal size/mm^3	0.22 × 0.16 × 0.07
Radiation	Cu Kα (λ = 1.54184)
2Θ range for data collection/°	5.228 to 136.502
Index ranges	-41 ≤ h ≤ 41, -17 ≤ k ≤ 17, -35 ≤ l ≤ 35
Reflections collected	66878
Independent reflections	13871 [R_{int} = 0.0416, R_{sigma} = 0.0282]
Data/restraints/parameters	13871/139/668
Goodness-of-fit on F^2	1.047
Final R indexes [I>=2σ (I)]	R_1 = 0.0902, wR_2 = 0.2796
Largest diff. peak/hole / e Å^{-3}	1.43/-1.18
SQUEEZE analysis indicated one void with a volume of 736Å^3 and electron count of 194. This is equivalent to 4.04 cyclohexane molecules or 1.49 BBA-8,12-OMe molecules.	

Table S17 BBA-8,12-OMe 4_1_96c crystallographic table

CCDC Deposition Number	2342871
Empirical formula	$C_{123.96}H_{93.85}I_{18}N_{36}O_{2.16}Zn_{9}$
Formula weight	4994.73
Temperature/K	100(2)
Crystal system	monoclinic
Space group	C2/c
a/Å	78.371(2)
b/Å	14.9291(4)
c/Å	41.6991(13)
α = γ /°	90
β/°	90
Volume/Å^3	48066(3)
Z	8
ρ_{calc}g/cm^3	1.38
μ/mm^{-1}	19.428
F(000)	18647
Crystal size/mm^3	0.3 × 0.11 × 0.08
Radiation	Cu Kα (λ = 1.54184)
2Θ range for data collection/°	4.302 to 136.496
Index ranges	-94 ≤ h ≤ 69, -17 ≤ k ≤ 17, -50 ≤ l ≤ 48
Reflections collected	150110
Independent reflections	43655 [R_{int} = 0.0872, R_{sigma} = 0.0782]
Data/restraints/parameters	43655/790/2252
Goodness-of-fit on F^2	1.033
Final R indexes [I>=2σ (I)]	R_1 = 0.1319, wR_2 = 0.3716
Largest diff. peak/hole / e Å^{-3}	1.65/-2.61

SQUEEZE analysis indicated two voids with volumes of 655 and 1196Å^3 and electron counts of 191 and 311 respectively. This is equivalent to 3.98 and 6.48 cyclohexane molecules, or 1.47 and 2.39 BBA-8,12-OMe molecules.

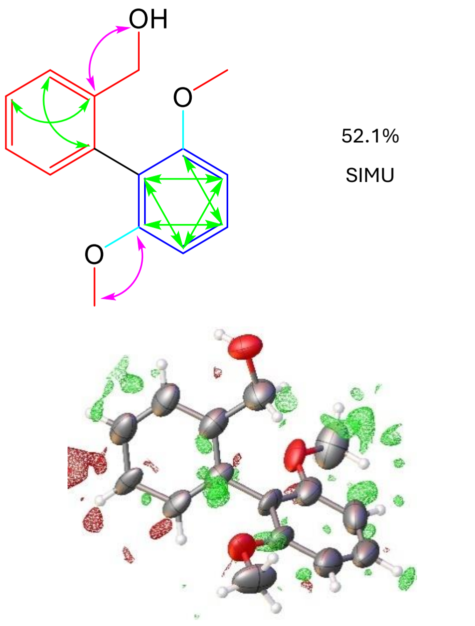

Figure S9 Geometric restraints and thermal ellipsoid (50% probability) for 4_1_96c Site A

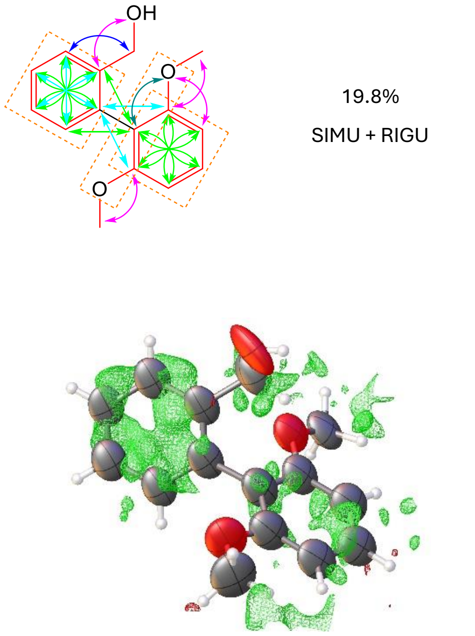

Figure S10 Geometric restraints and thermal ellipsoid (50% probability) for 4_1_96c Site B

Table S18 BBA-8,12-OMe 4_5_48a crystallographic table

CCDC Deposition Number	2342872
Empirical formula	$C_{36}H_{24}I_6N_{12}Zn_3$
Formula weight	1582.18
Temperature/K	100(2)
Crystal system	monoclinic
Space group	C2/c
a/Å	34.0757(18)
b/Å	15.0947(6)
c/Å	29.186(2)
α = γ /°	90
β/°	90
Volume/Å^3	14811.6(15)
Z	8
ρ_{calc}g/cm^3	1.419
μ/mm^{-1}	20.978
F(000)	5856
Crystal size/mm^3	0.21 × 0.13 × 0.05
Radiation	Cu Kα (λ = 1.54184)
2Θ range for data collection/°	5.258 to 136.494
Index ranges	-31 ≤ h ≤ 40, -18 ≤ k ≤ 14, -34 ≤ l ≤ 35
Reflections collected	41560
Independent reflections	13370 [R_{int} = 0.0504, R_{sigma} = 0.0366]
Data/restraints/parameters	13370/23/494
Goodness-of-fit on F^2	1.177
Final R indexes [I>=2σ (I)]	R_1 = 0.0918, wR_2 = 0.3039
Largest diff. peak/hole / e Å^{-3}	1.38/-0.73
No guests or solvent were identified from the electron density map, SQUEEZE analysis indicated one void with a volume of 796Å^3 and electron count of 191. This is equivalent to 3.98 cyclohexane molecules or 1.47 BBA-8,12-OMe molecules.	

Table S19 BBA-8,12-OMe 4_5_48b crystallographic table

CCDC Deposition Number	2342873
Empirical formula	$C_{36}H_{24}I_6N_{12}Zn_3$
Formula weight	1582.18
Temperature/K	100(2)
Crystal system	monoclinic
Space group	C2/c
a/Å	34.3094(9)
b/Å	15.0823(3)
c/Å	29.8583(10)
α = γ /°	90
β/°	90
Volume/Å^3	15190.0(7)
Z	8
ρ_{calc}g/cm^3	1.384
μ/mm^{-1}	20.455
F(000)	5856
Crystal size/mm^3	0.11 × 0.08 × 0.05
Radiation	Cu Kα (λ = 1.54184)
2Θ range for data collection/°	5.24 to 136.49
Index ranges	-36 ≤ h ≤ 41, -18 ≤ k ≤ 14, -35 ≤ l ≤ 35
Reflections collected	47451
Independent reflections	13821 [R_{int} = 0.0497, R_{sigma} = 0.0455]
Data/restraints/parameters	13821/21/614
Goodness-of-fit on F^2	1.053
Final R indexes [I>=2σ (I)]	R_1 = 0.0505, wR_2 = 0.1441
Largest diff. peak/hole / e Å^{-3}	0.73/-0.66
No guests or solvent were identified from the electron density map, SQUEEZE analysis indicated one void with a volume of 863Å^3 and electron count of 206. This is equivalent to 4.29 cyclohexane molecules or 1.58 BBA-8,12-OMe molecules.	

Table S20 BBA-8,12-OMe 4_5_48c crystallographic table

CCDC Deposition Number	2342874
Empirical formula	$C_{38.4}H_{28.81}I_6N_{12}Zn_3$
Formula weight	1615.89
Temperature/K	100(2)
Crystal system	monoclinic
Space group	C2/c
a/Å	34.3373(11)
b/Å	15.0894(3)
c/Å	29.5872(11)
α = γ /°	90
β/°	90
Volume/Å^3	15083.4(8)
Z	8
ρ_{calc}g/cm^3	1.423
μ/mm^{-1}	20.612
F(000)	6010
Crystal size/mm^3	0.21 × 0.11 × 0.06
Radiation	Cu Kα (λ = 1.54184)
2Θ range for data collection/°	5.232 to 136.5
Index ranges	-40 ≤ h ≤ 41, -18 ≤ k ≤ 9, -35 ≤ l ≤ 34
Reflections collected	44524
Independent reflections	13605 [R_{int} = 0.0346, R_{sigma} = 0.0348]
Data/restraints/parameters	13605/251/733
Goodness-of-fit on F^2	1.052
Final R indexes [I>=2σ (I)]	R_1 = 0.0746, wR_2 = 0.2339
Largest diff. peak/hole / e Å^{-3}	1.49/-0.92
SQUEEZE analysis indicated one void with a volume of 530Å^3 and electron count of 115. This is equivalent to 2.40 cyclohexane molecules or 0.88 BBA-8,12-OMe molecules.	

Table S21 BBA-8,12-OMe 4_10_24a crystallographic table

CCDC Deposition Number	2342875
Empirical formula	$C_{36}H_{24}I_6N_{12}Zn_3$
Formula weight	1582.18
Temperature/K	100(2)
Crystal system	monoclinic
Space group	C2/c
a/Å	33.994(5)
b/Å	15.0640(7)
c/Å	29.295(5)
α = γ /°	90
β/°	90
Volume/Å^3	14803(3)
Z	8
ρ_{calc}g/cm^3	1.42
μ/mm^{-1}	20.99
F(000)	5856
Crystal size/mm^3	0.17 × 0.15 × 0.07
Radiation	Cu Kα (λ = 1.54184)
2Θ range for data collection/°	5.27 to 136.5
Index ranges	-40 ≤ h ≤ 40, -9 ≤ k ≤ 18, -35 ≤ l ≤ 35
Reflections collected	43344
Independent reflections	13329 [R_{int} = 0.1134, R_{sigma} = 0.0853]
Data/restraints/parameters	13329/72/418
Goodness-of-fit on F^2	1.266
Final R indexes [I>=2σ (I)]	R_1 = 0.1223, wR_2 = 0.3725
Largest diff. peak/hole / e Å^{-3}	0.97/-0.79
No guests or solvent were identified from the electron density map, SQUEEZE analysis indicated one void with a volume of 797Å^3 and electron count of 206. This is equivalent to 4.29 cyclohexane molecules or 1.58 BBA-8,12-OMe molecules.	

Table S22 BBA-8,12-OMe 4_10_24b crystallographic table

CCDC Deposition Number	2342876
Empirical formula	$C_{36}H_{24}I_6N_{12}Zn_3$
Formula weight	1582.18
Temperature/K	100(2)
Crystal system	monoclinic
Space group	C2/c
a/Å	34.2193(11)
b/Å	15.0730(4)
c/Å	29.5041(12)
α = γ /°	90
β/°	90
Volume/Å^3	14987.6(9)
Z	8
ρ_{calc}g/cm^3	1.402
μ/mm^{-1}	20.732
F(000)	5856
Crystal size/mm^3	0.17 × 0.15 × 0.07
Radiation	Cu Kα (λ = 1.54184)
2Θ range for data collection/°	5.244 to 136.502
Index ranges	-38 ≤ h ≤ 41, -18 ≤ k ≤ 17, -35 ≤ l ≤ 35
Reflections collected	48842
Independent reflections	13632 [R_{int} = 0.0355, R_{sigma} = 0.0284]
Data/restraints/parameters	13632/93/612
Goodness-of-fit on F^2	1.101
Final R indexes [I>=2σ (I)]	R_1 = 0.0597, wR_2 = 0.1960
Largest diff. peak/hole / e Å^{-3}	0.82/-0.74
No guests or solvent were identified from the electron density map, SQUEEZE analysis indicated one void with a volume of 834Å^3 and electron count of 234. This is equivalent to 4.88 cyclohexane molecules or 1.80 BBA-8,12-OMe molecules.	

Table S23 BBA-8,12-OMe 4_10_24c crystallographic table

CCDC Deposition Number	2342877
Empirical formula	$C_{36}H_{24}I_6N_{12}Zn_3$
Formula weight	1582.18
Temperature/K	100.00(10)
Crystal system	monoclinic
Space group	C2/c
a/Å	34.3369(12)
b/Å	15.0656(4)
c/Å	29.8140(12)
α = γ /°	90
β/°	90
Volume/Å^3	15166.7(9)
Z	8
ρ_{calc}g/cm^3	1.386
μ/mm^{-1}	20.487
F(000)	5856
Crystal size/mm^3	0.2 × 0.14 × 0.09
Radiation	Cu Kα (λ = 1.54184)
2Θ range for data collection/°	5.234 to 136.504
Index ranges	-36 ≤ h ≤ 41, -18 ≤ k ≤ 18, -35 ≤ l ≤ 35
Reflections collected	70635
Independent reflections	13796 [R_{int} = 0.0428, R_{sigma} = 0.0256]
Data/restraints/parameters	13796/21/622
Goodness-of-fit on F^2	1.095
Final R indexes [I>=2σ (I)]	R_1 = 0.0653, wR_2 = 0.2153
Largest diff. peak/hole / e Å^{-3}	0.71/-0.81
No guests or solvent were identified from the electron density map, SQUEEZE analysis indicated one void with a volume of 846Å^3 and electron count of 222. This is equivalent to 4.63 cyclohexane molecules or 1.71 BBA-8,12-OMe molecules.	

Table S24 BBA-8,12-OMe 4_10_96a crystallographic table

CCDC Deposition Number	2342878
Empirical formula	$C_{36}H_{24}I_6N_{12}Zn_3$
Formula weight	1582.18
Temperature/K	100.00(10)
Crystal system	monoclinic
Space group	C2/c
a/Å	34.4940(13)
b/Å	15.0307(3)
c/Å	30.3347(13)
α = γ /°	90
β/°	90
Volume/Å^3	15435.8(10)
Z	8
ρ_{calc}g/cm^3	1.362
μ/mm^{-1}	20.13
F(000)	5856
Crystal size/mm^3	0.12 × 0.12 × 0.07
Radiation	Cu Kα (λ = 1.54184)
2Θ range for data collection/°	5.22 to 136.496
Index ranges	-40 ≤ h ≤ 41, -16 ≤ k ≤ 17, -35 ≤ l ≤ 36
Reflections collected	69226
Independent reflections	14036 [R_{int} = 0.0495, R_{sigma} = 0.0347]
Data/restraints/parameters	14036/32/617
Goodness-of-fit on F^2	1.093
Final R indexes [I>=2σ (I)]	R_1 = 0.0844, wR_2 = 0.2720
Largest diff. peak/hole / e Å^{-3}	1.03/-0.67
No guests or solvent were identified from the electron density map, SQUEEZE analysis indicated one void with a volume of 906Å^3 and electron count of 238. This is equivalent to 4.96 cyclohexane molecules or 1.83 BBA-8,12-OMe molecules.	

Table S25 BBA-8,12-OMe 4_10_96b crystallographic table

CCDC Deposition Number	2342879
Empirical formula	$C_{38.78}H_{26.97}I_6N_{12}O_{0.56}Zn_3$
Formula weight	1627.49
Temperature/K	100(2)
Crystal system	monoclinic
Space group	C2/c
a/Å	34.7075(8)
b/Å	14.9722(3)
c/Å	31.1023(9)
α = γ /°	90
β/°	90
Volume/Å³	15798.1(7)
Z	8
ρ_{calc}g/cm³	1.369
μ/mm⁻¹	19.689
F(000)	6049
Crystal size/mm³	0.16 × 0.15 × 0.07
Radiation	Cu Kα (λ = 1.54184)
2Θ range for data collection/°	5.21 to 136.502
Index ranges	-40 ≤ h ≤ 41, -18 ≤ k ≤ 17, -36 ≤ l ≤ 37
Reflections collected	53948
Independent reflections	14397 [R_{int} = 0.0416, R_{sigma} = 0.0348]
Data/restraints/parameters	14397/293/753
Goodness-of-fit on F²	1.049
Final R indexes [I>=2σ (I)]	R_1 = 0.0936, wR_2 = 0.3026
Largest diff. peak/hole / e Å⁻³	1.22/-1.13

SQUEEZE analysis indicated one void with a volume of 646Å³ and electron count of 154. This is equivalent to 3.21 cyclohexane molecules or 1.18 BBA-8,12-OMe molecules.

Manual weighting scheme applied.

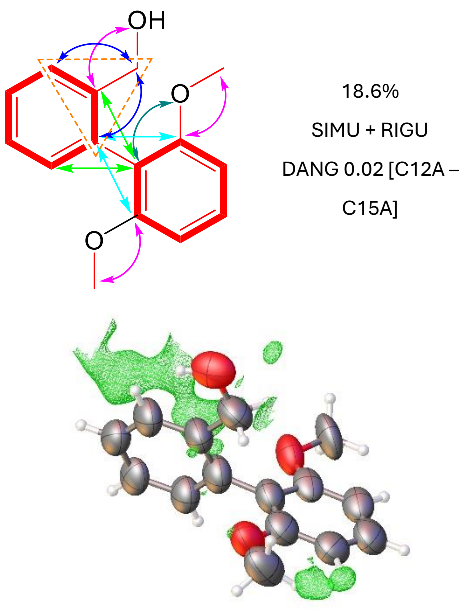

Figure S11 Geometric restraints and thermal ellipsoid (50% probability) for 4_10_96b Site A

Table S26 BBA-8,12-OMe 4_10_96c crystallographic table

CCDC Deposition Number	2342880
Empirical formula	$C_{36}H_{24}I_6N_{12}Zn_3$
Formula weight	1582.18
Temperature/K	100.00(10)
Crystal system	monoclinic
Space group	C2/c
a/Å	34.5442(16)
b/Å	15.0184(3)
c/Å	30.549(2)
α = γ /°	90
β/°	90
Volume/Å^3	15539.0(13)
Z	8
ρ_{calc}g/cm^3	1.353
μ/mm^{-1}	19.996
F(000)	5856
Crystal size/mm^3	0.16 × 0.11 × 0.07
Radiation	Cu Kα (λ = 1.54184)
2Θ range for data collection/°	5.218 to 136.494
Index ranges	-41 ≤ h ≤ 41, -11 ≤ k ≤ 17, -36 ≤ l ≤ 36
Reflections collected	51976
Independent reflections	14144 [R_{int} = 0.0488, R_{sigma} = 0.0344]
Data/restraints/parameters	14144/18/631
Goodness-of-fit on F^2	1.111
Final R indexes [I>=2σ (I)]	R_1 = 0.0920, wR_2 = 0.2866
Largest diff. peak/hole / e Å^{-3}	1.14/-1.13
No guests or solvent were identified from the electron density map, SQUEEZE analysis indicated one void with a volume of 923Å^3 and electron count of 290. This is equivalent to 6.04 cyclohexane molecules or 2.23 BBA-8,12-OMe molecules.	

Table S27 BBA-8,12-OMe 25_1_24a crystallographic table

CCDC Deposition Number	2342881
Empirical formula	$C_{122.39}H_{90.76}I_{18}N_{36}O_{2.15}Zn_{9}$
Formula weight	4972.58
Temperature/K	100(2)
Crystal system	monoclinic
Space group	C2/c
a/Å	78.142(4)
b/Å	14.9754(2)
c/Å	41.6691(13)
α = γ /°	90
β/°	99.702(4)
Volume/Å^3	48064(3)
Z	8
ρ_{calc}g/cm^3	1.374
μ/mm^{-1}	19.427
F(000)	18546
Crystal size/mm^3	0.29 × 0.16 × 0.06
Radiation	Cu Kα (λ = 1.54184)
2Θ range for data collection/°	4.522 to 136.5
Index ranges	-94 ≤ h ≤ 93, -12 ≤ k ≤ 17, -48 ≤ l ≤ 50
Reflections collected	158843
Independent reflections	41978 [R_{int} = 0.0663, R_{sigma} = 0.0466]
Data/restraints/parameters	41978/569/2102
Goodness-of-fit on F^2	1.062
Final R indexes [I>=2σ (I)]	R_1 = 0.1401, wR_2 = 0.3563
Largest diff. peak/hole / e Å^{-3}	1.50/-1.37

SQUEEZE analysis indicated three voids with volumes of 413, 86, and 1820Å^3 and electron counts of 44, 12, and 304 respectively. This is equivalent to 1.13, 0.25, and 6.33 cyclohexane molecules, or 0.42, 0.09, and 2.34 BBA-8,12-OMe molecules

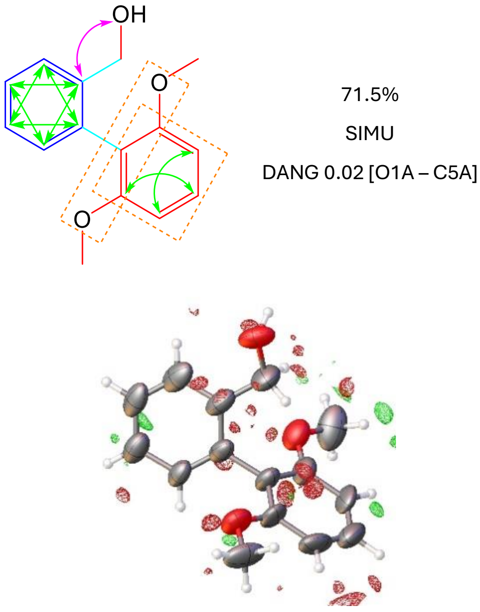

Figure S12 Geometric restraints and thermal ellipsoid (50% probability) for 25_1_24a Site A

Table S28 BBA-8,12-OMe 25_1_24b crystallographic table

CCDC Deposition Number	2342882
Empirical formula	$C_{36}H_{24}I_6N_{12}Zn_3$
Formula weight	1582.18
Temperature/K	100(2)
Crystal system	monoclinic
Space group	C2/c
a/Å	34.4567(17)
b/Å	15.0045(4)
c/Å	30.444(2)
α = γ /°	90
β/°	101.219(6)
Volume/Å^3	15438.9(14)
Z	8
ρ_{calc}g/cm^3	1.361
μ/mm^{-1}	20.126
F(000)	5856
Crystal size/mm^3	0.2 × 0.11 × 0.04
Radiation	Cu Kα (λ = 1.54184)
2Θ range for data collection/°	5.23 to 136.502
Index ranges	-41 ≤ h ≤ 35, -18 ≤ k ≤ 15, -36 ≤ l ≤ 36
Reflections collected	51251
Independent reflections	14055 [R_{int} = 0.0601, R_{sigma} = 0.0496]
Data/restraints/parameters	14055/27/625
Goodness-of-fit on F^2	1.085
Final R indexes [I>=2σ (I)]	R_1 = 0.0791, wR_2 = 0.2581
Largest diff. peak/hole / e Å^{-3}	1.05/-0.73
No guests or solvent were identified from the electron density map, SQUEEZE analysis indicated one void with a volume of 892Å^3 and electron count of 243. This is equivalent to 5.06 cyclohexane molecules or 1.87 BBA-8,12-OMe molecules.	

Table S29 BBA-8,12-OMe 25_1_24c crystallographic table

CCDC Deposition Number	2342883
Empirical formula	$C_{36}H_{24}I_6N_{12}Zn_3$
Formula weight	1582.18
Temperature/K	100.00(10)
Crystal system	monoclinic
Space group	C2/c
a/Å	34.3531(8)
b/Å	15.0637(4)
c/Å	29.9781(11)
α = γ /°	90
β/°	100.634(3)
Volume/Å^3	15246.8(8)
Z	8
ρ_{calc}g/cm^3	1.379
μ/mm^{-1}	20.379
F(000)	5856
Crystal size/mm^3	0.29 × 0.17 × 0.1
Radiation	Cu Kα (λ = 1.54184)
2Θ range for data collection/°	5.234 to 136.496
Index ranges	-40 ≤ h ≤ 41, -14 ≤ k ≤ 18, -34 ≤ l ≤ 36
Reflections collected	50575
Independent reflections	13839 [R_{int} = 0.0540, R_{sigma} = 0.0457]
Data/restraints/parameters	13839/21/614
Goodness-of-fit on F^2	1.084
Final R indexes [I>=2σ (I)]	R_1 = 0.0708, wR_2 = 0.2214
Largest diff. peak/hole / e Å^{-3}	1.19/-1.00
No guests or solvent were identified from the electron density map, SQUEEZE analysis indicated one void with a volume of 883Å^3 and electron count of 221. This is equivalent to 4.60 cyclohexane molecules or 1.70 BBA-8,12-OMe molecules.	

Table S30 BBA-8,12-OMe 25_1_48a crystallographic table

CCDC Deposition Number	2342884
Empirical formula	$C_{78.84}H_{55.3}I_{12}N_{24}O_{1.37}Zn_6$
Formula weight	3275.85
Temperature/K	100(2)
Crystal system	monoclinic
Space group	P2/n
a/Å	31.4246(9)
b/Å	14.9857(3)
c/Å	34.4395(9)
α = γ /°	90
β/°	90
Volume/Å^3	15865.2(7)
Z	4
ρ_{calc}g/cm^3	1.371
μ/mm^{-1}	19.611
F(000)	6093
Crystal size/mm^3	0.14 × 0.12 × 0.06
Radiation	Cu Kα (λ = 1.54184)
2Θ range for data collection/°	4.274 to 136.502
Index ranges	-36 ≤ h ≤ 37, -16 ≤ k ≤ 18, -36 ≤ l ≤ 41
Reflections collected	98894
Independent reflections	28477 [R_{int} = 0.0575, R_{sigma} = 0.0554]
Data/restraints/parameters	28477/329/1345
Goodness-of-fit on F^2	1.034
Final R indexes [I>=2σ (I)]	R_1 = 0.0981, wR_2 = 0.3075
Largest diff. peak/hole / e Å^{-3}	1.29/-0.70
SQUEEZE analysis indicated two voids with volumes of 981 and 674Å^3 and electron counts of 258 and 146 respectively. This is equivalent to 5.38 and 3.04 cyclohexane molecules, or 1.98 and 1.12 BBA-8,12-OMe molecules	

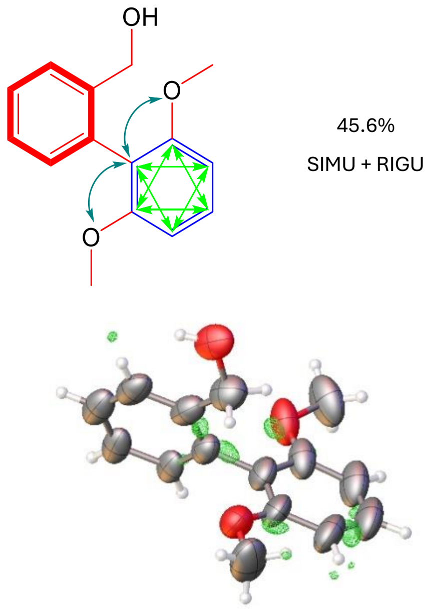

Figure S13 Geometric restraints and thermal ellipsoid (50% probability) for 25_1_48a Site A

Table S31 BBA-8,12-OMe 25_1_48b crystallographic table

CCDC Deposition Number	2342885
Empirical formula	$C_{36}H_{24}I_6N_{12}Zn_3$
Formula weight	1582.18
Temperature/K	100(2)
Crystal system	monoclinic
Space group	C2/c
a/Å	34.458(2)
b/Å	15.0078(4)
c/Å	30.486(3)
α = γ /°	90
β/°	90
Volume/Å^3	15463.2(18)
Z	8
ρ_{calc}g/cm^3	1.359
μ/mm^{-1}	20.094
F(000)	5856
Crystal size/mm^3	0.18 × 0.15 × 0.05
Radiation	Cu Kα (λ = 1.54184)
2Θ range for data collection/°	5.23 to 136.496
Index ranges	-41 ≤ h ≤ 41, -12 ≤ k ≤ 18, -36 ≤ l ≤ 32
Reflections collected	51956
Independent reflections	14069 [R_{int} = 0.0553, R_{sigma} = 0.0426]
Data/restraints/parameters	14069/37/575
Goodness-of-fit on F^2	1.117
Final R indexes [I>=2σ (I)]	R_1 = 0.0899, wR_2 = 0.2951
Largest diff. peak/hole / e Å^{-3}	0.85/-0.74
No guests or solvent were identified from the electron density map, SQUEEZE analysis indicated one void with a volume of 901Å^3 and electron count of 282. This is equivalent to 5.88 cyclohexane molecules or 2.17 BBA-8,12-OMe molecules.	

Table S32 BBA-8,12-OMe 25_1_48c crystallographic table

CCDC Deposition Number	2342886
Empirical formula	$C_{129.97}H_{101.68}I_{18}N_{36}O_{3.06}Zn_9$
Formula weight	5089.17
Temperature/K	100(2)
Crystal system	monoclinic
Space group	C2/c
a/Å	78.5587(18)
b/Å	14.9697(2)
c/Å	41.6779(6)
α = γ /°	90
β/°	90
Volume/Å³	48229.6(15)
Z	8
ρ_{calc}g/cm³	1.402
μ/mm⁻¹	19.376
F(000)	19055
Crystal size/mm³	0.13 × 0.11 × 0.05
Radiation	Cu Kα (λ = 1.54184)
2Θ range for data collection/°	4.504 to 136.498
Index ranges	-94 ≤ h ≤ 94, -18 ≤ k ≤ 17, -50 ≤ l ≤ 43
Reflections collected	143224
Independent reflections	43855 [R_{int} = 0.0370, R_{sigma} = 0.0355]
Data/restraints/parameters	43855/999/2390
Goodness-of-fit on F²	1.045
Final R indexes [I>=2σ (I)]	R_1 = 0.0700, wR_2 = 0.2201
Largest diff. peak/hole / e Å⁻³	1.98/-1.08

SQUEEZE analysis indicated three voids with volumes of 392, 88, and 1060Å³ and electron counts of 76, 17, and 238 respectively. This is equivalent to 1.58, 0.35, and 4.96 cyclohexane molecules, or 0.58, 0.13, and 1.83 BBA-8,12-OMe molecules

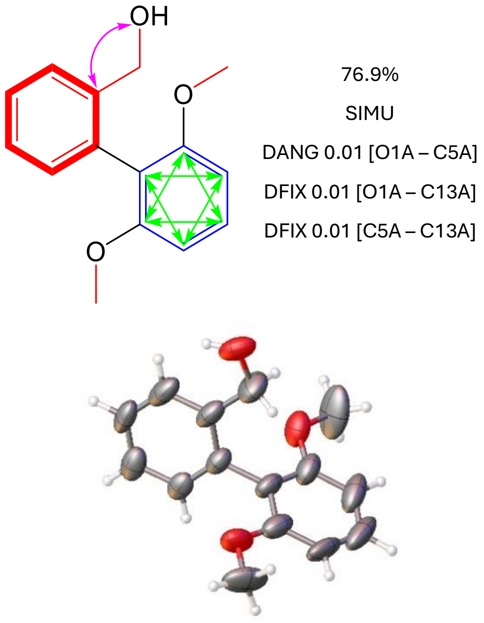

Figure S14 Geometric restraints and thermal ellipsoid (50% probability) for 25_1_48c Site A

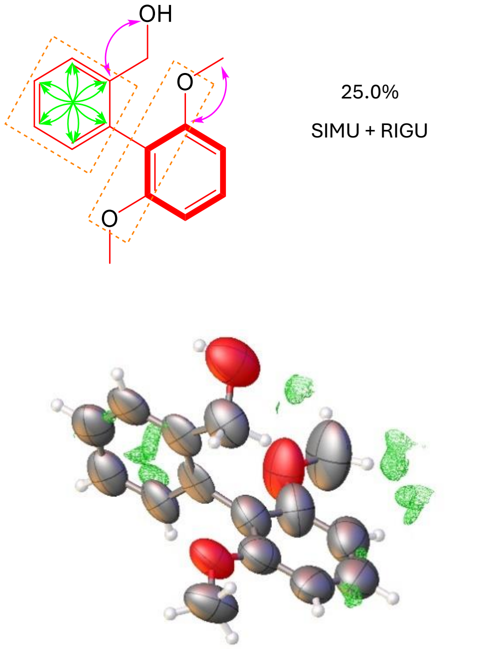

Figure S15 Geometric restraints and thermal ellipsoid (50% probability) for 25_1_48c Site B

Table S33 BBA-8,12-OMe 25_1_96a crystallographic table

CCDC Deposition Number	2342887
Empirical formula	$C_{119.57}H_{84.34}I_{18}N_{36}O_{2.31}Zn_9$
Formula weight	4934.99
Temperature/K	100(2)
Crystal system	monoclinic
Space group	C2/c
a/Å	78.8715(10)
b/Å	14.95020(10)
c/Å	41.6531(4)
α = γ /°	90
β/°	90
Volume/Å^3	48353.6(8)
Z	8
ρ_{calc}g/cm^3	1.356
μ/mm^{-1}	19.307
F(000)	18370
Crystal size/mm^3	0.21 × 0.19 × 0.08
Radiation	Cu Kα (λ = 1.54184)
2Θ range for data collection/°	4.31 to 136.502
Index ranges	-94 ≤ h ≤ 94, -17 ≤ k ≤ 18, -50 ≤ l ≤ 50
Reflections collected	157610
Independent reflections	43958 [R_{int} = 0.0498, R_{sigma} = 0.0413]
Data/restraints/parameters	43958/254/1925
Goodness-of-fit on F^2	1.055
Final R indexes [I>=2σ (I)]	R_1 = 0.0940, wR_2 = 0.2889
Largest diff. peak/hole / e Å^{-3}	1.77/-1.21

SQUEEZE analysis indicated two voids with volumes of 685 and 2008Å^3 and electron counts of 196 and 594 respectively. This is equivalent to 4.08 and 12.38 cyclohexane molecules, or 1.51 and 4.57 BBA-8,12-OMe molecules.

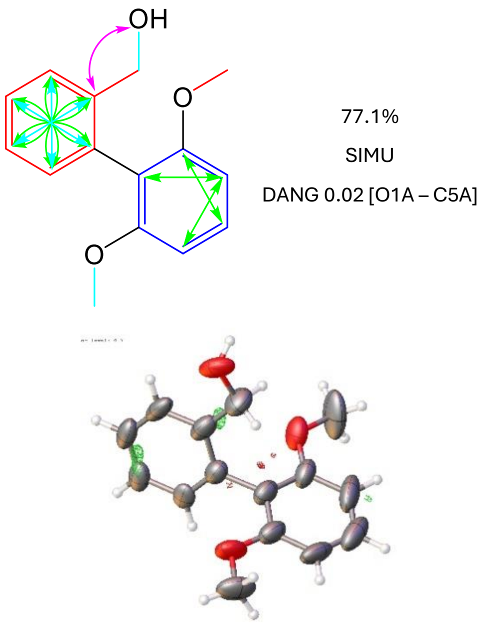

Figure S16 Geometric restraints and thermal ellipsoid (50% probability) for 25_1_96a Site A

Table S34 BBA-8,12-OMe 25_1_96b crystallographic table

CCDC Deposition Number	2342888
Empirical formula	$C_{118.5}H_{83.2}I_{18}N_{36}O_{2.1}Zn_9$
Formula weight	4917.61
Temperature/K	100(2)
Crystal system	monoclinic
Space group	C2/c
a/Å	78.606(2)
b/Å	14.9668(2)
c/Å	41.6349(6)
α = γ /°	90
β/°	90
Volume/Å^3	48219.6(16)
Z	8
ρ_{calc}g/cm^3	1.355
μ/mm^{-1}	19.358
F(000)	18296
Crystal size/mm^3	0.18 × 0.17 × 0.07
Radiation	Cu Kα (λ = 1.54184)
2Θ range for data collection/°	4.312 to 136.502
Index ranges	-94 ≤ h ≤ 94, -18 ≤ k ≤ 14, -50 ≤ l ≤ 50
Reflections collected	220686
Independent reflections	44036 [R_{int} = 0.0668, R_{sigma} = 0.0462]
Data/restraints/parameters	44036/292/1906
Goodness-of-fit on F^2	1.052
Final R indexes [I>=2σ (I)]	R_1 = 0.1121, wR_2 = 0.3256
Largest diff. peak/hole / e Å^{-3}	1.80/-1.23
SQUEEZE analysis indicated two voids with volumes of 666 and 1988Å^3 and electron counts of 183 and 540 respectively. This is equivalent to 3.81 and 11.25 cyclohexane molecules, or 1.41 and 4.15 BBA-8,12-OMe molecules.	

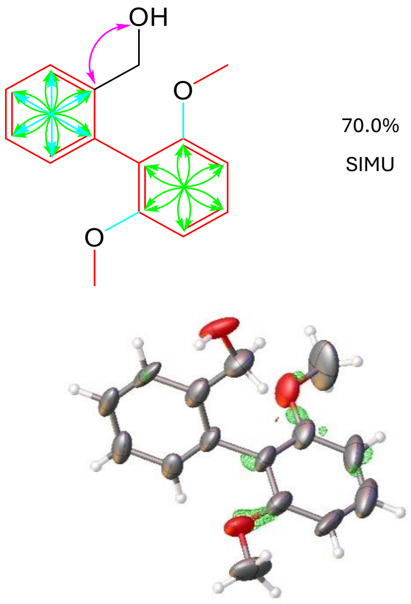

Figure S17 Geometric restraints and thermal ellipsoid (50% probability) for 25_1_96b Site A

Table S35 BBA-8,12-OMe 25_1_96c crystallographic table

CCDC Deposition Number	2342889
Empirical formula	$C_{122.16}H_{87.11}I_{18}N_{36}O_{2.83}Zn_9$
Formula weight	4977.17
Temperature/K	100.00(10)
Crystal system	monoclinic
Space group	C2/c
a/Å	77.257(6)
b/Å	15.0021(8)
c/Å	41.5124(18)
α = γ /°	90
β/°	90
Volume/Å^3	47332(5)
Z	8
ρ_{calc}g/cm^3	1.397
μ/mm^{-1}	19.73
F(000)	18550
Crystal size/mm^3	0.19 × 0.15 × 0.13
Radiation	Cu Kα (λ = 1.54184)
2Θ range for data collection/°	4.328 to 136.502
Index ranges	-92 ≤ h ≤ 92, -16 ≤ k ≤ 18, -45 ≤ l ≤ 50
Reflections collected	156082
Independent reflections	43077 [R_{int} = 0.0819, R_{sigma} = 0.0717]
Data/restraints/parameters	43077/1034/2310
Goodness-of-fit on F^2	1.033
Final R indexes [I>=2σ (I)]	R_1 = 0.1289, wR_2 = 0.3878
Largest diff. peak/hole / e Å^{-3}	1.37/-1.16
SQUEEZE analysis indicated two voids with volumes of 640 and 1894Å^3 and electron counts of 96 and 494 respectively. This is	

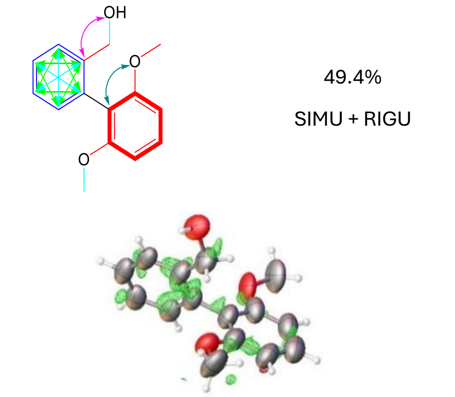

Figure S18 Geometric restraints and thermal ellipsoid (50% probability) for 25_1_96c Site A

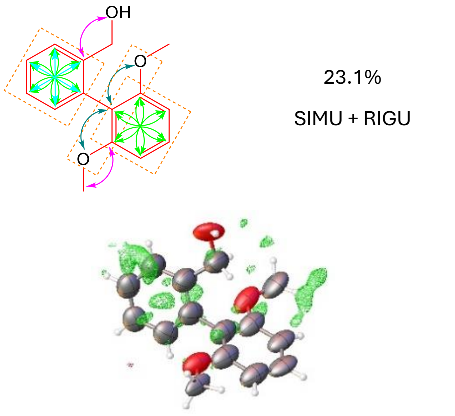

Figure S19 Geometric restraints and thermal ellipsoid (50% probability) for 25_1_96c Site B

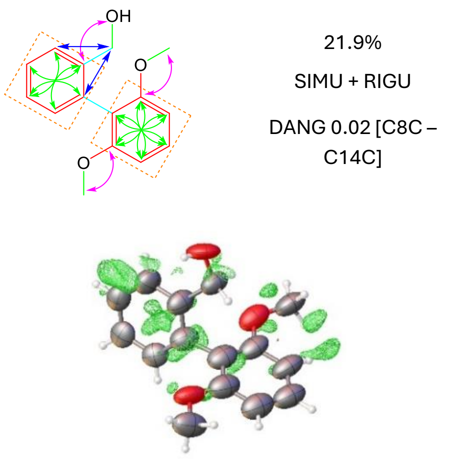

equivalent to 2.00 and 9.67 cyclohexane molecules, or 0.74 and 3.57 BBA-8,12-OMe molecules.<br>Manual weighting scheme applied.

Figure S20 Geometric restraints and thermal ellipsoid (50% probability) for 25_1_96c Site C

Table S36 BBA-8,12-OMe 25_5_24a crystallographic table

CCDC Deposition Number	2342890
Empirical formula	$C_{39.88}H_{28.14}I_6N_{12}O_{0.78}Zn_3$
Formula weight	1645.46
Temperature/K	100(2)
Crystal system	monoclinic
Space group	C2/c
a/Å	34.5084(12)
b/Å	14.9904(4)
c/Å	31.4216(16)
α = γ /°	90
β/°	101.916(4)
Volume/Å^3	15904.0(11)
Z	8
ρ_{calc}g/cm^3	1.374
μ/mm^{-1}	19.567
F(000)	6125
Crystal size/mm^3	0.21 × 0.1 × 0.05
Radiation	Cu Kα (λ = 1.54184)
2Θ range for data collection/°	5.234 to 136.492
Index ranges	-41 ≤ h ≤ 40, -17 ≤ k ≤ 17, -37 ≤ l ≤ 37
Reflections collected	49830
Independent reflections	14369 [R_{int} = 0.0497, R_{sigma} = 0.0373]
Data/restraints/parameters	14369/334/784
Goodness-of-fit on F^2	1.073
Final R indexes [I>=2σ (I)]	R_1 = 0.1258, wR_2 = 0.3841
Largest diff. peak/hole / e Å^{-3}	1.11/-1.18

SQUEEZE analysis indicated one void with a volume of 635Å^3 and electron count of 83. This is equivalent to 1.73 cyclohexane molecules or 0.64 BBA-8,12-OMe molecules.

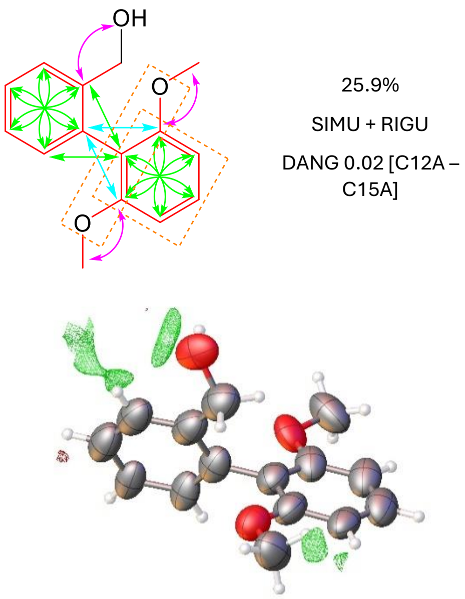

Figure S21 Geometric restraints and thermal ellipsoid (50% probability) for 25_5_24a Site A

Table S37 BBA-8,12-OMe 25_5_24b crystallographic table

CCDC Deposition Number	2342891
Empirical formula	$C_{36}H_{24}I_6N_{12}Zn_3$
Formula weight	1582.18
Temperature/K	100(2)
Crystal system	monoclinic
Space group	C2/c
a/Å	34.4933(18)
b/Å	15.0280(4)
c/Å	30.223(2)
α = γ /°	90
β/°	101.026(6)
Volume/Å^3	15377.6(15)
Z	8
ρ_{calc}g/cm^3	1.367
μ/mm^{-1}	20.206
F(000)	5856
Crystal size/mm^3	0.21 × 0.12 × 0.12
Radiation	Cu Kα (λ = 1.54184)
2Θ range for data collection/°	5.22 to 136.492
Index ranges	-41 ≤ h ≤ 41, -9 ≤ k ≤ 18, -36 ≤ l ≤ 36
Reflections collected	47934
Independent reflections	13978 [R_{int} = 0.0671, R_{sigma} = 0.0422]
Data/restraints/parameters	13978/93/605
Goodness-of-fit on F^2	1.119
Final R indexes [I>=2σ (I)]	R_1 = 0.1390, wR_2 = 0.4334
Largest diff. peak/hole / e Å^{-3}	1.56/-1.38
No guests or solvent were identified from the electron density map, SQUEEZE analysis indicated one void with a volume of 909Å^3 and electron count of 275. This is equivalent to 5.73 cyclohexane molecules or 2.12 BBA-8,12-OMe molecules. Manual weighting scheme applied.	

Table S38 BBA-8,12-OMe 25_5_24b crystallographic table

CCDC Deposition Number	2342892
Empirical formula	$C_{82.77}H_{61.36}I_{12}N_{24}O_{1.75}Zn_6$
Formula weight	3335.25
Temperature/K	100.00(10)
Crystal system	monoclinic
Space group	P2/n
a/Å	31.387(2)
b/Å	15.0302(5)
c/Å	34.2836(16)
α = γ /°	90
β/°	101.803(5)
Volume/Å^3	15831.6(14)
Z	4
ρ_{calc}g/cm^3	1.399
μ/mm^{-1}	19.665
F(000)	6224
Crystal size/mm^3	0.2 × 0.14 × 0.07
Radiation	Cu Kα (λ = 1.54184)
2Θ range for data collection/°	4.278 to 136.502
Index ranges	-37 ≤ h ≤ 37, -18 ≤ k ≤ 17, -41 ≤ l ≤ 39
Reflections collected	97471
Independent reflections	28696 [R_{int} = 0.0679, R_{sigma} = 0.0479]
Data/restraints/parameters	28696/585/1379
Goodness-of-fit on F^2	1.064
Final R indexes [I>=2σ (I)]	R_1 = 0.1782, wR_2 = 0.4197
Largest diff. peak/hole / e Å^{-3}	1.89/-1.68
SQUEEZE analysis indicated three voids with volumes of 412, 943, and 70Å^3 and electron counts of 108, 352, and 22 respectively. This is equivalent to 2.25, 7.33, and 0.46 cyclohexane molecules, or 0.83, 2.71, and 0.17 BBA-8,12-OMe molecules	

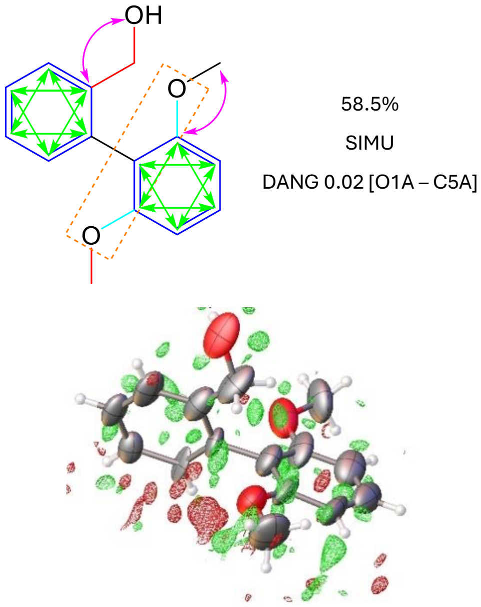

Figure S22 Geometric restraints and thermal ellipsoid (50% probability) for 25_5_24c Site A

Table S39 BBA-8,12-OMe 25_5_96a crystallographic table

CCDC Deposition Number	2342893
Empirical formula	$C_{118.14}H_{82.81}I_{18}N_{36}O_{2.03}Zn_{9}$
Formula weight	4911.63
Temperature/K	100.00(10)
Crystal system	monoclinic
Space group	C2/c
a/Å	78.813(2)
b/Å	14.9644(3)
c/Å	41.6570(9)
α = γ /°	90
β/°	90
Volume/Å^3	48374(2)
Z	8
ρ_{calc}g/cm^3	1.349
μ/mm^{-1}	19.295
F(000)	18271
Crystal size/mm^3	0.21 × 0.11 × 0.06
Radiation	Cu Kα (λ = 1.54184)
2Θ range for data collection/°	4.308 to 136.498
Index ranges	-94 ≤ h ≤ 94, -17 ≤ k ≤ 17, -50 ≤ l ≤ 49
Reflections collected	173702
Independent reflections	43411 [R_{int} = 0.0794, R_{sigma} = 0.0624]
Data/restraints/parameters	43411/684/1929
Goodness-of-fit on F^2	1.018
Final R indexes [I>=2σ (I)]	R_1 = 0.1424, wR_2 = 0.4140
Largest diff. peak/hole / e Å^{-3}	1.74/-1.27

SQUEEZE analysis indicated two voids with volumes of 698 and 2032Å^3 and electron counts of 167 and 486 respectively. This is equivalent to 3.48 and 10.13 cyclohexane molecules, or 1.28 and 3.74 BBA-8,12-OMe molecules.

Manual weighting scheme applied.

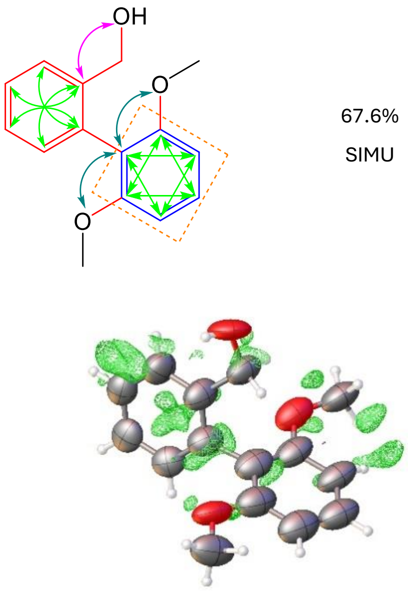

Figure S23 Geometric restraints and thermal ellipsoid (50% probability) for 25_5_96a Site A

Table S40 BBA-8,12-OMe 25_5_96b crystallographic table

CCDC Deposition Number	2342894
Empirical formula	$C_{133.79}H_{110.09}I_{18}N_{36}O_{2.89}Zn_9$
Formula weight	5140.97
Temperature/K	100(2)
Crystal system	monoclinic
Space group	C2/c
a/Å	78.4975(16)
b/Å	14.9701(2)
c/Å	41.6228(6)
α = γ /°	90
β/°	90
Volume/Å³	48167.3(14)
Z	8
ρ_{calc}g/cm³	1.418
μ/mm⁻¹	19.406
F(000)	19296
Crystal size/mm³	0.19 × 0.18 × 0.07
Radiation	Cu Kα (λ = 1.54184)
2Θ range for data collection/°	4.312 to 136.502
Index ranges	-94 ≤ h ≤ 94, -17 ≤ k ≤ 18, -50 ≤ l ≤ 50
Reflections collected	224407
Independent reflections	44037 [R_{int} = 0.0620, R_{sigma} = 0.0434]
Data/restraints/parameters	44037/1166/2493
Goodness-of-fit on F²	1.081
Final R indexes [I>=2σ (I)]	R_1 = 0.0931, wR_2 = 0.2665
Largest diff. peak/hole / e Å⁻³	1.96/-1.25
SQUEEZE analysis indicated three voids with volumes of 90, 242, and 862Å³ and electron counts of 16, 48, and 192 respectively. This is equivalent to 0.33, 1.00, and 4.00 cyclohexane molecules, or 0.12, 0.37, and 1.48 BBA-8,12-OMe molecules	

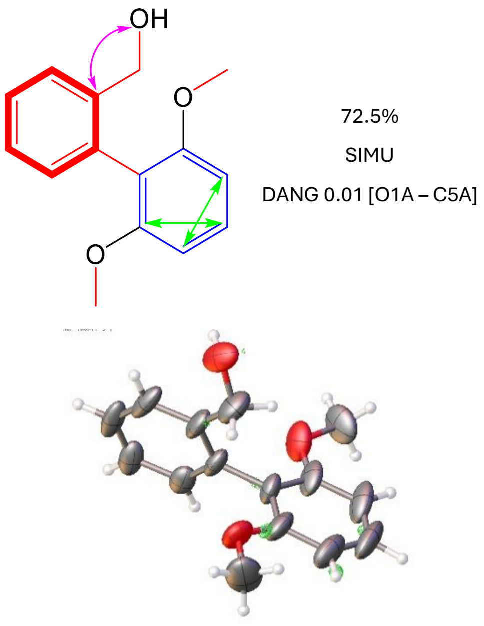

Figure S24 Geometric restraints and thermal ellipsoid (50% probability) for 25_5_96b Site A

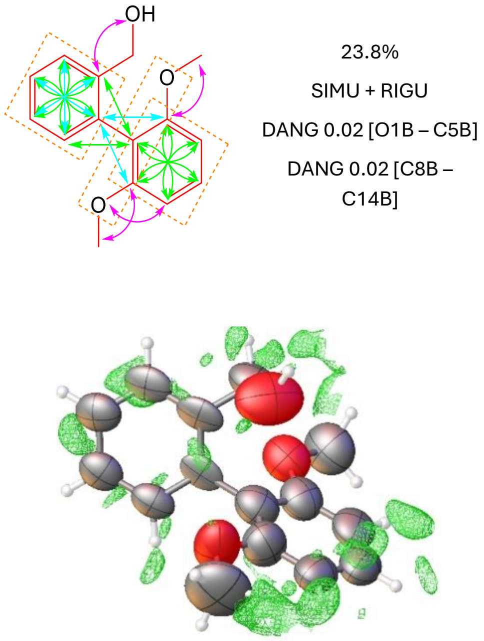

Figure S25 Geometric restraints and thermal ellipsoid (50% probability) for 25_5_96b Site B

Table S41 BBA-8,12-OMe 25_5_96c crystallographic table

CCDC Deposition Number	2342895
Empirical formula	$C_{39.49}H_{27.72}I_6N_{12}O_{0.7}Zn_3$
Formula weight	1638.96
Temperature/K	100.00(10)
Crystal system	monoclinic
Space group	C2/c
a/Å	34.4145(9)
b/Å	14.9918(3)
c/Å	31.0574(10)
α = γ /°	90
β/°	90
Volume/Å^3	15688.2(7)
Z	8
ρ_{calc}g/cm^3	1.388
μ/mm^{-1}	19.833
F(000)	6098
Crystal size/mm^3	0.29 × 0.09 × 0.06
Radiation	Cu Kα (λ = 1.54184)
2Θ range for data collection/°	5.246 to 136.5
Index ranges	-41 ≤ h ≤ 40, -17 ≤ k ≤ 12, -36 ≤ l ≤ 37
Reflections collected	64404
Independent reflections	14239 [R_{int} = 0.0417, R_{sigma} = 0.0328]
Data/restraints/parameters	14239/287/753
Goodness-of-fit on F^2	1.063
Final R indexes [I>=2σ (I)]	R_1 = 0.0933, wR_2 = 0.3037
Largest diff. peak/hole / e Å^{-3}	1.07/-1.24

SQUEEZE analysis indicated one void with a volume of 628Å^3 and electron count of 158. This is equivalent to 1.73 cyclohexane molecules or 0.64 BBA-8,12-OMe molecules.

Manual weighting scheme applied.

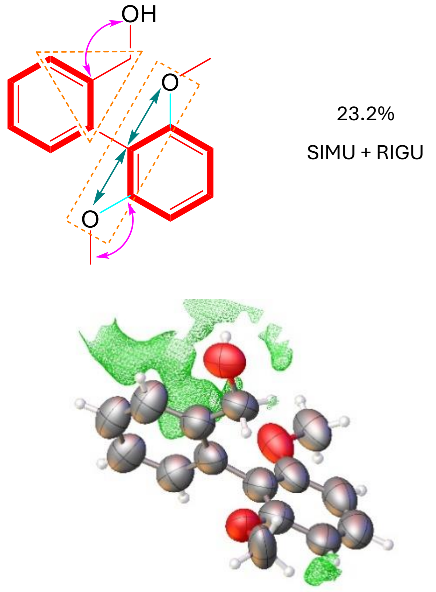

Figure S26 Geometric restraints and thermal ellipsoid (50% probability) for 25_5_96c Site A

Table S42 BBA-8,12-OMe 25_10_24a crystallographic table

CCDC Deposition Number	2342896
Empirical formula	$C_{116}H_{81.63}I_{18}N_{36}O_{1.37}Zn_9$
Formula weight	4874.19
Temperature/K	100.00(10)
Crystal system	monoclinic
Space group	C2/c
a/Å	77.629(3)
b/Å	15.0298(4)
c/Å	41.5537(14)
α = γ /°	90
β/°	90
Volume/Å^3	47679(3)
Z	8
ρ_{calc}g/cm^3	1.358
μ/mm^{-1}	19.57
F(000)	18116
Crystal size/mm^3	0.18 × 0.08 × 0.05
Radiation	Cu Kα (λ = 1.54184)
2Θ range for data collection/°	4.52 to 136.502
Index ranges	-93 ≤ h ≤ 90, -14 ≤ k ≤ 18, -50 ≤ l ≤ 47
Reflections collected	190484
Independent reflections	43357 [R_{int} = 0.0988, R_{sigma} = 0.0786]
Data/restraints/parameters	43357/494/2024
Goodness-of-fit on F^2	1.017
Final R indexes [I>=2σ (I)]	R_1 = 0.1606, wR_2 = 0.4665
Largest diff. peak/hole / e Å^{-3}	2.34/-1.90

SQUEEZE analysis indicated three voids with volumes of 400, 94, and 1910Å^3 and electron counts of 103, 16, and 580 respectively. This is equivalent to 2.14, 0.33, and 12.08 cyclohexane molecules, or 0.79, 0.12, and 4.46 BBA-8,12-OMe molecules.

Manual weighting scheme applied.

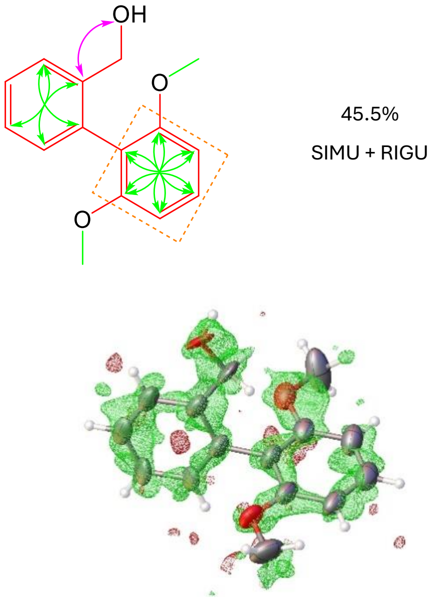

Figure S27 Geometric restraints and thermal ellipsoid (50% probability) for 25_10_24a Site A

Table S43 BBA-8,12-OMe 25_10_24b crystallographic table

CCDC Deposition Number	2342897
Empirical formula	$C_{115.54}H_{80.04}I_{18}N_{36}O_{1.51}Zn_9$
Formula weight	4869.29
Temperature/K	100(2)
Crystal system	monoclinic
Space group	C2/c
a/Å	78.288(6)
b/Å	14.9666(7)
c/Å	41.6314(19)
α = γ /°	90
β/°	90
Volume/Å^3	48051(5)
Z	8
ρ_{calc}g/cm^3	1.346
μ/mm^{-1}	19.418
F(000)	18091
Crystal size/mm^3	0.2 × 0.08 × 0.04
Radiation	Cu Kα (λ = 1.54184)
2Θ range for data collection/°	4.31 to 136.502
Index ranges	-94 ≤ h ≤ 94, -17 ≤ k ≤ 18, -50 ≤ l ≤ 49
Reflections collected	325146
Independent reflections	43965 [R_{int} = 0.2215, R_{sigma} = 0.0995]
Data/restraints/parameters	43965/532/1941
Goodness-of-fit on F^2	1.028
Final R indexes [I>=2σ (I)]	R_1 = 0.1636, wR_2 = 0.4455
Largest diff. peak/hole / e Å^{-3}	1.79/-1.21

SQUEEZE analysis indicated two voids with volumes of 686 and 1905Å^3 and electron counts of 173 and 530 respectively. This is equivalent to 3.60 and 11.04 cyclohexane molecules, or 1.33 and 4.07 BBA-8,12-OMe molecules.

Manual weighting scheme applied.

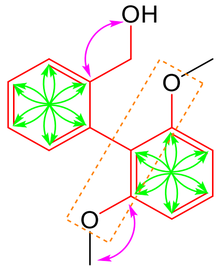

50.2%

SIMU + RIGU

DANG 0.02 [O1A – C5A]

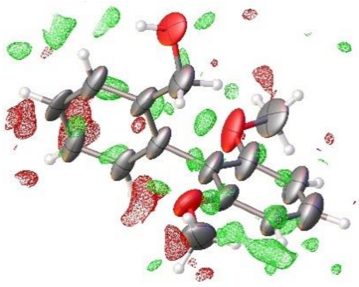

Figure S28 Geometric restraints and thermal ellipsoid (50% probability) for 25_10_24b Site A

Table S44 BBA-8,12-OMe 25_10_24c crystallographic table

CCDC Deposition Number	2342898
Empirical formula	$C_{36}H_{24}I_6N_{12}Zn_3$
Formula weight	1582.18
Temperature/K	100.00(10)
Crystal system	monoclinic
Space group	C2/c
a/Å	34.4156(9)
b/Å	15.0804(3)
c/Å	30.0607(16)
α = γ /°	90
β/°	90
Volume/Å^3	15329.8(10)
Z	8
ρ_{calc}g/cm^3	1.371
μ/mm^{-1}	20.269
F(000)	5856
Crystal size/mm^3	0.16 × 0.11 × 0.06
Radiation	Cu Kα (λ = 1.54184)
2Θ range for data collection/°	5.226 to 136.486
Index ranges	-41 ≤ h ≤ 35, -18 ≤ k ≤ 17, -36 ≤ l ≤ 36
Reflections collected	53521
Independent reflections	13960 [R_{int} = 0.0507, R_{sigma} = 0.0437]
Data/restraints/parameters	13960/22/616
Goodness-of-fit on F^2	1.087
Final R indexes [I>=2σ (I)]	R_1 = 0.0864, wR_2 = 0.2709
Largest diff. peak/hole / e Å^{-3}	1.78/-0.98
No guests or solvent were identified from the electron density map, SQUEEZE analysis indicated one void with a volume of 888Å^3 and electron count of 250. This is equivalent to 5.21 cyclohexane molecules or 1.92 BBA-8,12-OMe molecules.	

Table S45 BBA-8,12-OMe 25_10_48a crystallographic table

CCDC Deposition Number	2342899
Empirical formula	$C_{87.42}H_{68.09}I_{12}N_{24}O_{2.31}Zn_6$
Formula weight	3406.68
Temperature/K	100(2)
Crystal system	monoclinic
Space group	P2/n
a/Å	31.5433(11)
b/Å	15.0035(3)
c/Å	34.4229(12)
α = γ /°	90
β/°	90
Volume/Å^3	15936.5(9)
Z	4
ρ_{calc}g/cm^3	1.42
μ/mm^{-1}	19.55
F(000)	6380
Crystal size/mm^3	0.15 × 0.12 × 0.07
Radiation	Cu Kα (λ = 1.54184)
2Θ range for data collection/°	5.248 to 136.502
Index ranges	-38 ≤ h ≤ 38, -16 ≤ k ≤ 18, -40 ≤ l ≤ 41
Reflections collected	103867
Independent reflections	28969 [R_{int} = 0.0559, R_{sigma} = 0.0408]
Data/restraints/parameters	28969/794/1675
Goodness-of-fit on F^2	1.05
Final R indexes [I>=2σ (I)]	R_1 = 0.0978, wR_2 = 0.2843
Largest diff. peak/hole / e Å^{-3}	1.24/-0.92

Exchange site B was located over a two-fold symmetry axis and was modelled in PART -1. SQUEEZE analysis indicated three voids with volumes of 416, 484, and 90Å^3 and electron counts of 82, 114, and 18 respectively. This is equivalent to 1.71, 2.38, and 0.38 cyclohexane molecules, or 0.63, 0.88, and 0.14 BBA-8,12-OMe molecules

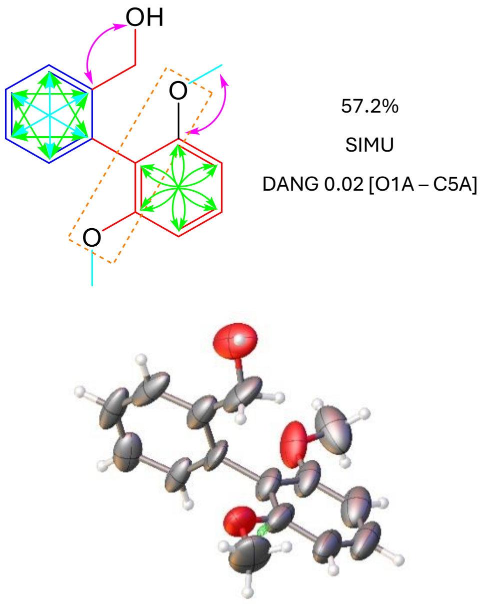

Figure S29 Geometric restraints and thermal ellipsoid (50% probability) for 25_10_48a Site A

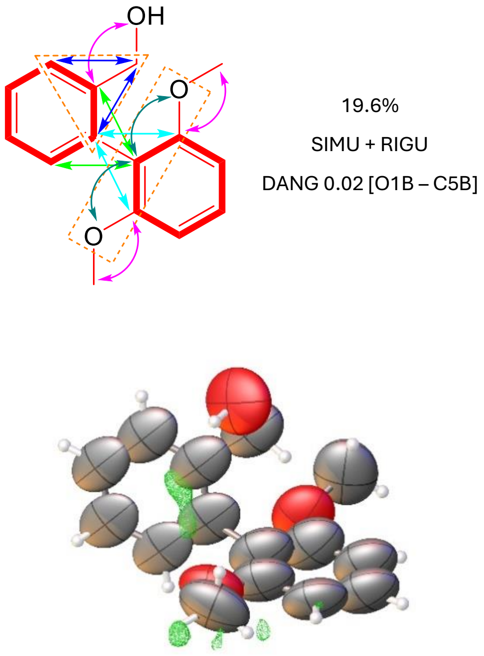

Figure S30 Geometric restraints and thermal ellipsoid (50% probability) for 25_10_48a Site B

Table S46 BBA-8,12-OMe 25_10_48b crystallographic table

CCDC Deposition Number	2342900
Empirical formula	$C_{36}H_{24}I_6N_{12}Zn_3$
Formula weight	1582.18
Temperature/K	100(2)
Crystal system	monoclinic
Space group	C2/c
a/Å	34.4800(19)
b/Å	15.0207(4)
c/Å	30.258(2)
α = γ /°	90
β/°	90
Volume/Å^3	15379.3(14)
Z	8
ρ_{calc}g/cm^3	1.367
μ/mm^{-1}	20.204
F(000)	5856
Crystal size/mm^3	0.23 × 0.11 × 0.07
Radiation	Cu Kα (λ = 1.54184)
2Θ range for data collection/°	5.224 to 136.48
Index ranges	-41 ≤ h ≤ 41, -7 ≤ k ≤ 17, -36 ≤ l ≤ 36
Reflections collected	45320
Independent reflections	13874 [R_{int} = 0.0429, R_{sigma} = 0.0356]
Data/restraints/parameters	13874/34/617
Goodness-of-fit on F^2	1.082
Final R indexes [I>=2σ (I)]	R_1 = 0.0678, wR_2 = 0.2253
Largest diff. peak/hole / e Å^{-3}	0.89/-0.62

No guests or solvent were identified from the electron density map, SQUEEZE analysis indicated one void with a volume of 885Å^3 and electron count of 234. This is equivalent to 4.88 cyclohexane molecules or 1.80 BBA-8,12-OMe molecules.

Table S47 BBA-8,12-OMe 25_10_48c crystallographic table

CCDC Deposition Number	2342901
Empirical formula	$C_{143}H_{124.58}I_{18}N_{36}O_{3.73}Zn_9$
Formula weight	5279.69
Temperature/K	100(2)
Crystal system	monoclinic
Space group	C2/c
a/Å	78.834(3)
b/Å	14.9690(3)
c/Å	41.7028(12)
α = γ /°	90
β/°	90
Volume/Å^3	48435(2)
Z	8
ρ_{calc}g/cm^3	1.448
μ/mm^{-1}	19.317
F(000)	19908
Crystal size/mm^3	0.22 × 0.13 × 0.05
Radiation	Cu Kα (λ = 1.54184)
2Θ range for data collection/°	4.306 to 136.5
Index ranges	-93 ≤ h ≤ 94, -18 ≤ k ≤ 18, -44 ≤ l ≤ 50
Reflections collected	166422
Independent reflections	44038 [R_{int} = 0.0696, R_{sigma} = 0.0590]
Data/restraints/parameters	44038/1335/2502
Goodness-of-fit on F^2	1.062
Final R indexes [I>=2σ (I)]	R_1 = 0.1201, wR_2 = 0.3400
Largest diff. peak/hole / e Å^{-3}	2.20/-1.77

SQUEEZE analysis indicated three voids with volumes of 95, 38, and 1045Å^3 and electron counts of 26, 6, and 240 respectively. This is equivalent to 0.54, 0.13, and 5.00 cyclohexane molecules, or 0.20, 0.05, and 1.85 BBA-8,12-OMe molecules

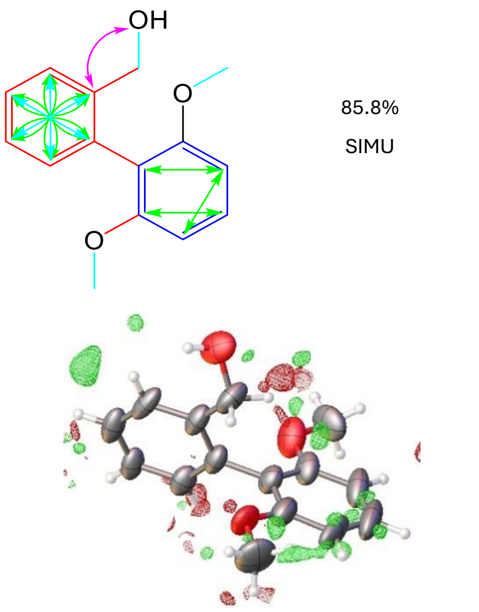

Figure S31 Geometric restraints and thermal ellipsoid (50% probability) for 25_10_48c Site A

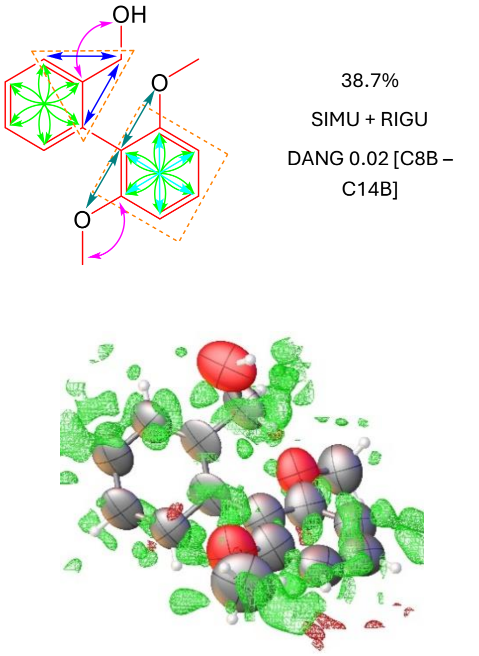

Figure S32 Geometric restraints and thermal ellipsoid (50% probability) for 25_10_48c Site B

Table S48 BBA-8,12-OMe 50_1_24a crystallographic table

CCDC Deposition Number	2342842
Empirical formula	$C_{82.19}H_{58.83}I_{12}N_{24}O_{2.04}Zn_6$
Formula weight	3330.36
Temperature/K	100(2)
Crystal system	Monoclinic
Space group	P2/n
a/Å	31.4571(13)
b/Å	14.9918(3)
c/Å	34.3610(12)
α = γ /°	90
β/°	101.834(3)
Volume/Å^3	15860.2(9)
Z	4
ρ_{calc}g/cm^3	1.395
μ/mm^{-1}	19.630
F(000)	6209.0
Crystal size/mm^3	0.23 × 0.15 × 0.08
Radiation	Cu Kα (λ = 1.54184)
2Θ range for data collection/°	5.256 to 136.502
Index ranges	-37 ≤ h ≤ 37, -17 ≤ k ≤ 18, -41 ≤ l ≤ 40
Reflections collected	97907
Independent reflections	28916 [R_{int} = 0.0569, R_{sigma} = 0.0442]
Data/restraints/parameters	28916/494/1563
Goodness-of-fit on F^2	1.057
Final R indexes [I>=2σ (I)]	R_1 = 0.1135, wR_2 = 0.3243
Largest diff. peak/hole / e Å^{-3}	1.17/-0.83
Exchange site B was located over a two-fold symmetry axis and was modelled in PART -1. SQUEEZE analysis indicated two voids with volumes of 726 and 557Å^3 and electron counts of 168 and 156 respectively. This is equivalent to 3.50 and 3.25 cyclohexane molecules, or 1.29 and 1.20 BBA-8,12-OMe molecules.	

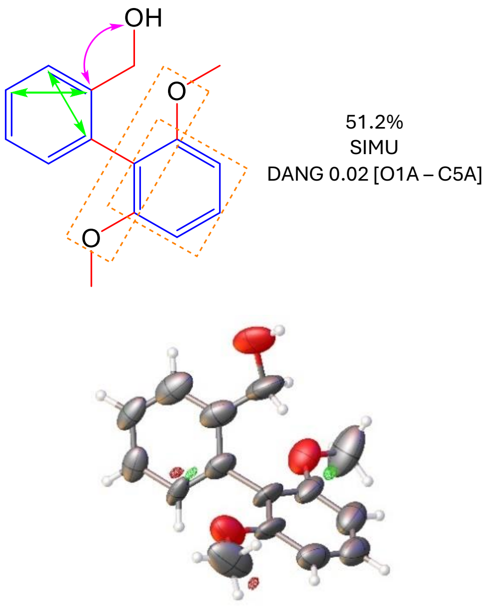

Figure S33 Geometric restraints and thermal ellipsoid (50% probability) for 50_1_24a Site A

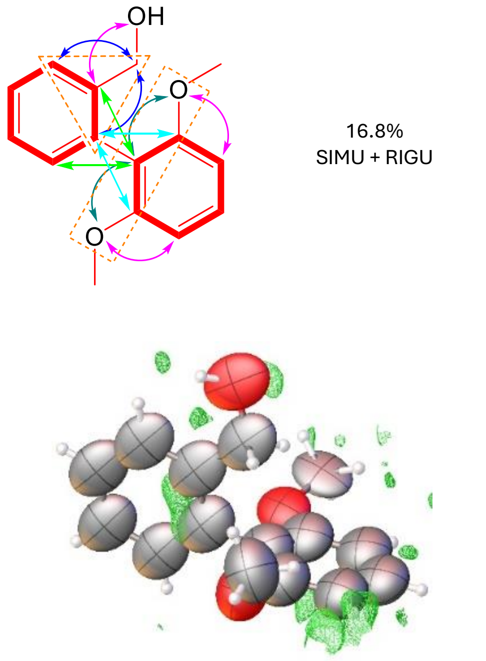

Figure S34 Geometric restraints and thermal ellipsoid (50% probability) for 50_1_24a Site B

Table S49 BBA-8,12-OMe 50_1_24b crystallographic table

CCDC Deposition Number	2342843
Empirical formula	$C_{40.06}H_{28.33}I_{6}N_{12}O_{0.81}Zn_{3}$
Formula weight	1648.34
Temperature/K	100(2)
Crystal system	monoclinic
Space group	C2/c
a/Å	34.561(2)
b/Å	14.9900(6)
c/Å	31.380(3)
α = γ /°	90
β/°	102.080(6)
Volume/Å^3	15897.2(18)
Z	8
ρ_{calc}g/cm^3	1.377
μ/mm^{-1}	19.576
F(000)	6138
Crystal size/mm^3	0.2 × 0.15 × 0.08
Radiation	Cu Kα (λ = 1.54184)
2Θ range for data collection/°	5.23 to 136.502
Index ranges	-37 ≤ h ≤ 41, -18 ≤ k ≤ 15, -37 ≤ l ≤ 36
Reflections collected	51491
Independent reflections	14455 [R_{int} = 0.0348, R_{sigma} = 0.0243]
Data/restraints/parameters	14455/309/757
Goodness-of-fit on F^2	1.067
Final R indexes [I>=2σ (I)]	R_1 = 0.0964, wR_2 = 0.3059
Largest diff. peak/hole / e Å^{-3}	1.09/-0.74
SQUEEZE analysis indicated one void with volume of 662Å^3 and electron counts of 155. This is equivalent to 3.23 cyclohexane molecules or 1.19 BBA-8,12-OMe molecules	

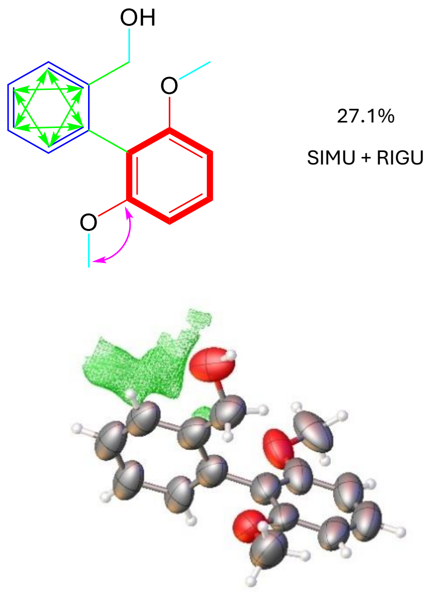

Figure S35 Geometric restraints and thermal ellipsoid (50% probability) for 50_1_24b Site A

Table S50 BBA-8,12-OMe 50_1_24c crystallographic table

CCDC Deposition Number	2342844
Empirical formula	$C_{85.05}H_{63.33}I_{12}N_{24}O_{2.09}Zn_6$
Formula weight	3369.96
Temperature/K	100(2)
Crystal system	monoclinic
Space group	P2/n
a/Å	31.5355(9)
b/Å	14.9866(4)
c/Å	34.4588(11)
α = γ /°	90
β/°	101.931(3)
Volume/Å^3	15933.8(8)
Z	4
ρ_{calc}g/cm^3	1.405
μ/mm^{-1}	19.546
F(000)	6297
Crystal size/mm^3	0.23 × 0.13 × 0.09
Radiation	Cu Kα (λ = 1.54184)
2Θ range for data collection/°	4.264 to 136.494
Index ranges	-29 ≤ h ≤ 37, -18 ≤ k ≤ 14, -41 ≤ l ≤ 41
Reflections collected	114741
Independent reflections	28949 [R_{int} = 0.0565, R_{sigma} = 0.0437]
Data/restraints/parameters	28949/350/1473
Goodness-of-fit on F^2	1.074
Final R indexes [I>=2σ (I)]	R_1 = 0.1220, wR_2 = 0.3346
Largest diff. peak/hole / e Å^{-3}	1.28/-1.15

Exchange site B was located over a two-fold symmetry axis and was modelled in PART -1. SQUEEZE analysis indicated three voids with volumes of 626, 397, and 90Å^3 and electron counts of 128, 76, and 156 respectively. This is equivalent to 2.67, 1.68, and 0.29 cyclohexane molecules, or 0.98, 0.58, and 0.11 BBA-8,12-OMe molecules

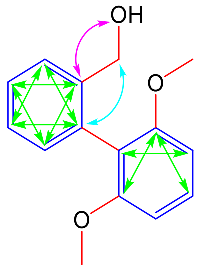

54.2%

SIMU

DANG 0.02 [O1A – C5A]

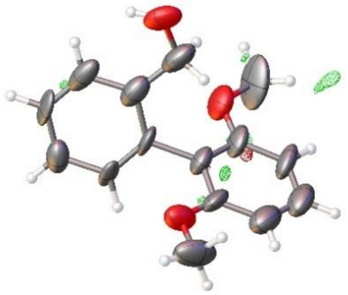

Figure S36 Geometric restraints and thermal ellipsoid (50% probability) for 50_1_24c Site A

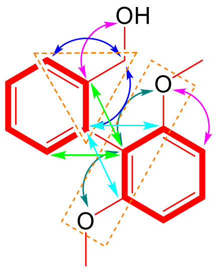

15.5%

Isotropic

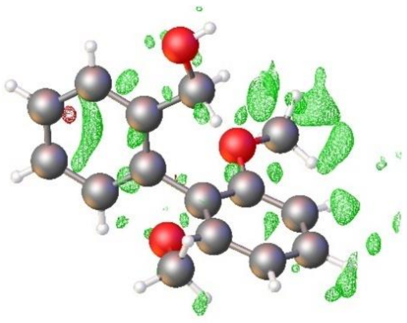

Figure S37 Geometric restraints and thermal ellipsoid (50% probability) for 50_1_24c Site B

Table S51 BBA-8,12-OMe 50_1_96a crystallographic table

CCDC Deposition Number	2342845
Empirical formula	$C_{84.39}H_{63.55}I_{12}N_{24}O_2Zn_6$
Formula weight	3360.86
Temperature/K	100(2)
Crystal system	monoclinic
Space group	P2/n
a/Å	31.6699(9)
b/Å	14.9780(2)
c/Å	34.5752(9)
α = γ /°	90
β/°	102.239(2)
Volume/Å³	16028.0(7)
Z	4
ρ_{calc}g/cm³	1.393
μ/mm⁻¹	19.429
F(000)	6280
Crystal size/mm³	0.15 × 0.12 × 0.06
Radiation	Cu Kα (λ = 1.54184)
2Θ range for data collection/°	3.438 to 136.504
Index ranges	-38 ≤ h ≤ 37, -17 ≤ k ≤ 12, -41 ≤ l ≤ 40
Reflections collected	101230
Independent reflections	29170 [R_{int} = 0.0485, R_{sigma} = 0.0431]
Data/restraints/parameters	29170/606/1521
Goodness-of-fit on F²	1.07
Final R indexes [I>=2σ (I)]	R_1 = 0.0913, wR_2 = 0.2721
Largest diff. peak/hole / e Å⁻³	1.41/-1.11

Exchange site B was located over a two-fold symmetry axis and was modelled in PART -1. SQUEEZE analysis indicated three voids with volumes of 430, 582, and 84Å³ and electron counts of 73, 139, and 15 respectively. This is equivalent to 1.52, 2.90, and 0.31 cyclohexane molecules, or 0.56, 1.07, and 0.12 BBA-8,12-OMe molecules

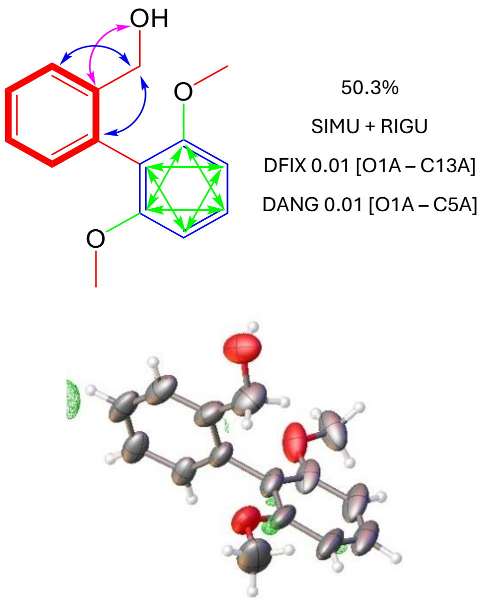

Figure S38 Geometric restraints and thermal ellipsoid (50% probability) for 50_1_96a Site A

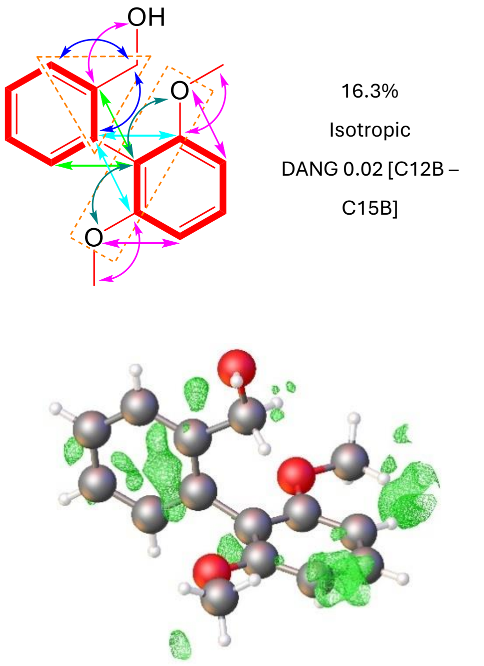

Figure S39 Geometric restraints and thermal ellipsoid (50% probability) for 50_1_96a Site B

Table S52 BBA-8,12-OMe 50_1_96b crystallographic table

CCDC Deposition Number	2342846
Empirical formula	$C_{84.05}H_{62.84}I_{12}N_{24}O_{1.98}Zn_6$
Formula weight	3355.66
Temperature/K	100(2)
Crystal system	monoclinic
Space group	P2/n
a/Å	31.5527(6)
b/Å	14.9736(2)
c/Å	34.5612(6)
α = γ /°	90
β/°	102.244(2)
Volume/Å^3	15957.3(5)
Z	4
ρ_{calc}g/cm^3	1.397
μ/mm^{-1}	19.514
F(000)	6268
Crystal size/mm^3	0.14 × 0.12 × 0.06
Radiation	Cu Kα (λ = 1.54184)
2Θ range for data collection/°	3.446 to 136.502
Index ranges	-37 ≤ h ≤ 38, -16 ≤ k ≤ 18, -38 ≤ l ≤ 41
Reflections collected	106916
Independent reflections	29111 [R_{int} = 0.0365, R_{sigma} = 0.0344]
Data/restraints/parameters	29111/471/1458
Goodness-of-fit on F^2	1.04
Final R indexes [I>=2σ (I)]	R_1 = 0.0795, wR_2 = 0.2495
Largest diff. peak/hole / e Å^{-3}	1.33/-0.79

Exchange site B was located over a two-fold symmetry axis and was modelled in PART -1. SQUEEZE analysis indicated two voids with volumes of 666 and 500Å^3 and electron counts of 130 and 119 respectively. This is equivalent to 2.71 and 2.48 cyclohexane molecules, or 1.00 and 0.92 BBA-8,12-OMe molecules.

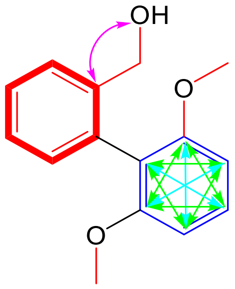

50.9%
SIMU + RIGU
DFIX 0.01 [O1A – C13A]
DANG 0.02 [O1A – C5A]

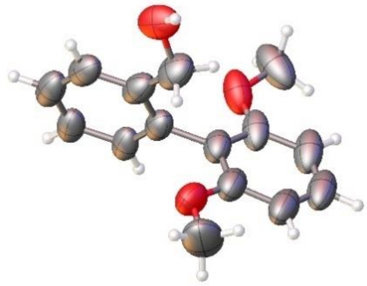

Figure S40 Geometric restraints and thermal ellipsoid (50% probability) for 50_1_96b Site A

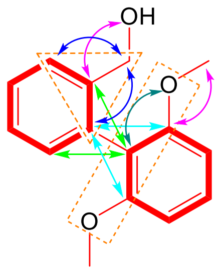

15.5%
Isotropic
DANG 0.02 [C12B – C15B]

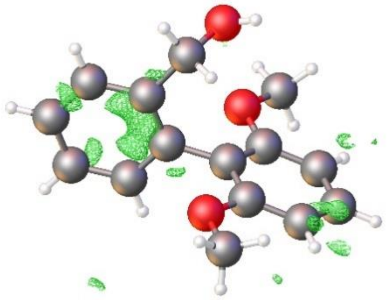

Figure S41 Geometric restraints and thermal ellipsoid (50% probability) for 50_1_96b Site B

Table S53 BBA-8,12-OMe 50_1_96c crystallographic table

CCDC Deposition Number	2342847
Empirical formula	$C_{84.73}H_{64.37}I_{12}N_{24}O_{1.95}Zn_{6}$
Formula weight	3365.01
Temperature/K	100(2)
Crystal system	monoclinic
Space group	P2/n
a/Å	31.5154(10)
b/Å	15.0261(5)
c/Å	34.3648(12)
α = γ /°	90
β/°	101.863(3)
Volume/Å^3	15926.0(9)
Z	4
ρ_{calc}g/cm^3	1.403
μ/mm^{-1}	19.554
F(000)	6290
Crystal size/mm^3	0.28 × 0.18 × 0.15
Radiation	Cu Kα (λ = 1.54184)
2Θ range for data collection/°	4.266 to 136.502
Index ranges	-37 ≤ h ≤ 37, -15 ≤ k ≤ 18, -40 ≤ l ≤ 41
Reflections collected	98591
Independent reflections	28948 [R_{int} = 0.0724, R_{sigma} = 0.0537]
Data/restraints/parameters	28948/495/1507
Goodness-of-fit on F^2	1.058
Final R indexes [I>=2σ (I)]	R_1 = 0.1268, wR_2 = 0.3622
Largest diff. peak/hole / e Å^{-3}	1.45/-1.00

SQUEEZE analysis indicated three voids with volumes of 424, 834, and 90Å^3 and electron counts of 78, 231, and 8 respectively. This is equivalent to 1.63, 4.81, and 0.17 cyclohexane molecules, or 0.60, 1.78, and 0.06 BBA-8,12-OMe molecules

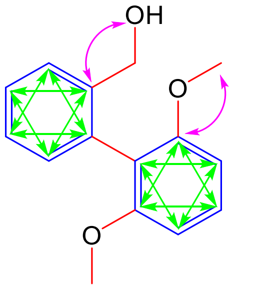

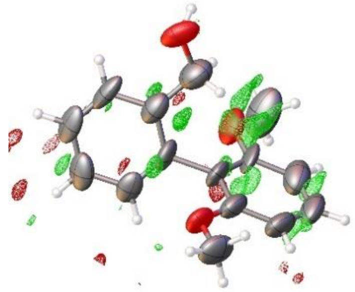

Figure S42 Geometric restraints and thermal ellipsoid (50% probability) for 50_1_96c Site A

Table S54 BBA-8,12-OMe 50_5_48a crystallographic table

CCDC Deposition Number	2342848
Empirical formula	$C_{40.42}H_{28.72}I_6N_{12}O_{0.89}Zn_3$
Formula weight	1654.24
Temperature/K	100(2)
Crystal system	monoclinic
Space group	C2/c
a/Å	34.5810(14)
b/Å	14.9867(3)
c/Å	31.5551(15)
α = γ /°	90
β/°	102.106(4)
Volume/Å^3	15989.9(11)
Z	8
ρ_{calc}g/cm^3	1.374
μ/mm^{-1}	19.466
F(000)	6163
Crystal size/mm^3	0.24 × 0.17 × 0.07
Radiation	Cu Kα (λ = 1.54184)
2Θ range for data collection/°	5.228 to 136.494
Index ranges	-41 ≤ h ≤ 41, -13 ≤ k ≤ 17, -37 ≤ l ≤ 38
Reflections collected	52421
Independent reflections	14569 [R_{int} = 0.0383, R_{sigma} = 0.0259]
Data/restraints/parameters	14569/336/758
Goodness-of-fit on F^2	1.052
Final R indexes [I>=2σ (I)]	R_1 = 0.1233, wR_2 = 0.3525
Largest diff. peak/hole / e Å^{-3}	1.13/-1.04
SQUEEZE analysis indicated one void with a volume of 668Å^3 and electron counts of 156. This is equivalent to 3.25 cyclohexane molecules, or 1.20 BBA-8,12-OMe molecules	

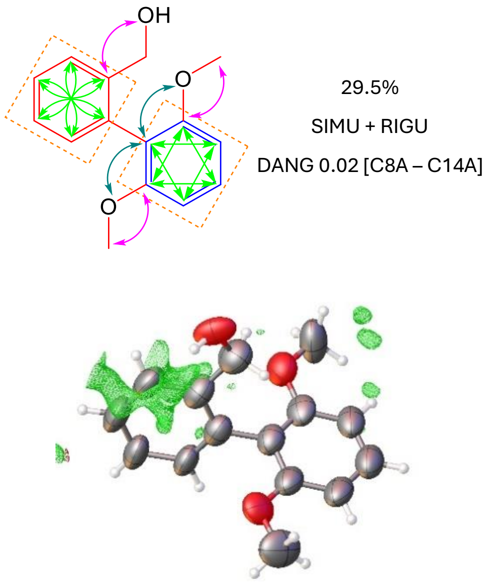

Figure S43 Geometric restraints and thermal ellipsoid (50% probability) for 50_5_48a Site A

Table S55 BBA-8,12-OMe 50_5_48b crystallographic table

CCDC Deposition Number	2342849
Empirical formula	$C_{94.73}H_{79.09}I_{12}N_{24}O_{3.08}Zn_6$
Formula weight	3517.93
Temperature/K	100(2)
Crystal system	monoclinic
Space group	P2/n
a/Å	31.6098(9)
b/Å	15.0289(2)
c/Å	34.3656(8)
α = γ /°	90
β/°	101.939(3)
Volume/Å^3	15972.6(6)
Z	4
ρ_{calc}g/cm^3	1.463
μ/mm^{-1}	19.528
F(000)	6624
Crystal size/mm^3	0.24 × 0.13 × 0.05
Radiation	Cu Kα (λ = 1.54184)
2Θ range for data collection/°	4.264 to 136.498
Index ranges	-35 ≤ h ≤ 38, -18 ≤ k ≤ 9, -41 ≤ l ≤ 40
Reflections collected	102894
Independent reflections	29074 [R_{int} = 0.0453, R_{sigma} = 0.0338]
Data/restraints/parameters	29074/824/1652
Goodness-of-fit on F^2	1.112
Final R indexes [I>=2σ (I)]	R_1 = 0.0957, wR_2 = 0.2741
Largest diff. peak/hole / e Å^{-3}	1.44/-0.85

Exchange site B was located over a two-fold symmetry axis and was modelled in PART -1. SQUEEZE analysis indicated three voids with volumes of 508, 238, and 92Å^3 and electron counts of 133, 50, and 26 respectively. This is equivalent to 2.77, 1.04, and 0.54 cyclohexane molecules, or 1.02, 0.38, and 0.20 BBA-8,12-OMe molecules

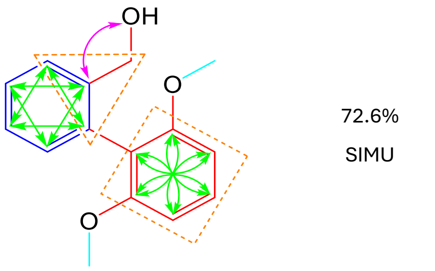

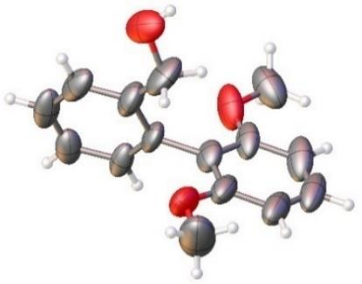

Figure S44 Geometric restraints and thermal ellipsoid (50% probability) for 50_5_48b Site A

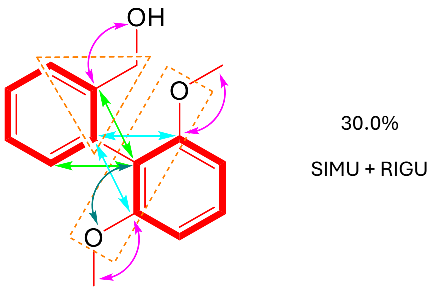

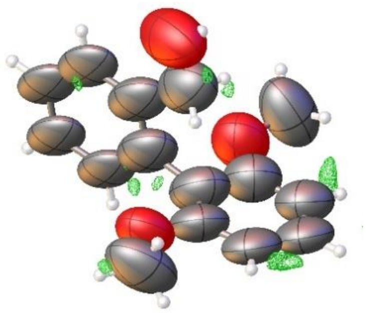

Figure S45 Geometric restraints and thermal ellipsoid (50% probability) for 50_5_48b Site B

Table S56 BBA-8,12-OMe 50_5_48c crystallographic table

CCDC Deposition Number	2342850
Empirical formula	$C_{36}H_{24}I_6N_{12}Zn_3$
Formula weight	1582.18
Temperature/K	100(2)
Crystal system	monoclinic
Space group	C2/c
a/Å	34.412(2)
b/Å	15.0303(5)
c/Å	29.994(3)
α = γ /°	90
β/°	100.858(7)
Volume/Å^3	15235.8(18)
Z	8
ρ_{calc}g/cm^3	1.38
μ/mm^{-1}	20.394
F(000)	5856
Crystal size/mm^3	0.19 × 0.17 × 0.07
Radiation	Cu Kα (λ = 1.54184)
2Θ range for data collection/°	5.23 to 136.5
Index ranges	-41 ≤ h ≤ 40, -18 ≤ k ≤ 16, -36 ≤ l ≤ 36
Reflections collected	90613
Independent reflections	13941 [R_{int} = 0.0825, R_{sigma} = 0.0437]
Data/restraints/parameters	13941/24/619
Goodness-of-fit on F^2	1.104
Final R indexes [I>=2σ (I)]	R_1 = 0.0899, wR_2 = 0.3041
Largest diff. peak/hole / e Å^{-3}	0.93/-0.75
No guests or solvent were identified from the electron density map, SQUEEZE analysis indicated one void with a volume of 847Å^3 and electron count of 232. This is equivalent to 4.83 cyclohexane molecules or 1.78 BBA-8,12-OMe molecules.	

Table S57 BBA-8,12-OMe 50_5_96a crystallographic table

CCDC Deposition Number	2342851
Empirical formula	$C_{88.38}H_{69.11}I_{12}N_{24}O_{2.48}Zn_6$
Formula weight	3422.02
Temperature/K	100(2)
Crystal system	monoclinic
Space group	P2/n
a/Å	31.5448(8)
b/Å	14.9824(2)
c/Å	34.3898(8)
α = γ /°	90
β/°	102.066(2)
Volume/Å^3	15894.1(6)
Z	4
ρ_{calc}g/cm^3	1.43
μ/mm^{-1}	19.606
F(000)	6413
Crystal size/mm^3	0.31 × 0.22 × 0.09
Radiation	Cu Kα (λ = 1.54184)
2Θ range for data collection/°	3.458 to 136.502
Index ranges	-37 ≤ h ≤ 38, -14 ≤ k ≤ 17, -41 ≤ l ≤ 38
Reflections collected	100011
Independent reflections	28888 [R_{int} = 0.0458, R_{sigma} = 0.0383]
Data/restraints/parameters	28888/793/1647
Goodness-of-fit on F^2	1.055
Final R indexes [I>=2σ (I)]	R_1 = 0.0825, wR_2 = 0.2550
Largest diff. peak/hole / e Å^{-3}	1.35/-1.03
Exchange site B was located over a two-fold symmetry axis and was modelled in PART -1. SQUEEZE analysis indicated three voids with volumes of 234, 594, and 66Å^3 and electron counts of 44, 144, and 16 respectively. This is equivalent to 0.92, 3.00, and 0.33 cyclohexane molecules, or 0.34, 1.11, and 0.12 BBA-8,12-OMe molecules	

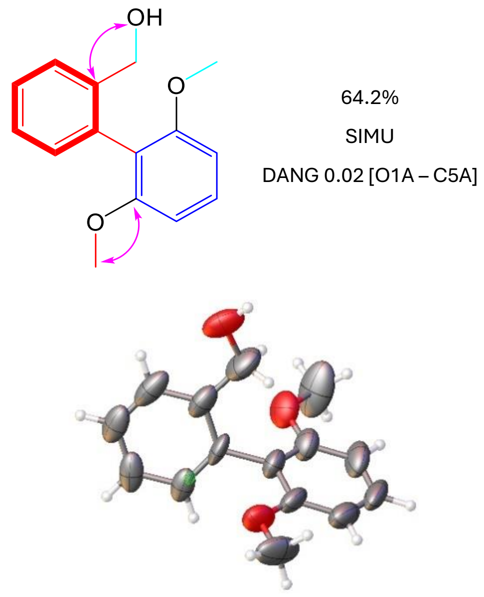

Figure S46 Geometric restraints and thermal ellipsoid (50% probability) for 50_5_96a Site A

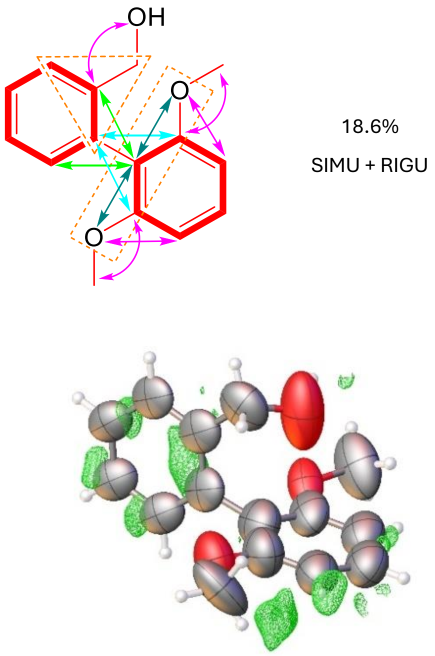

Figure S47 Geometric restraints and thermal ellipsoid (50% probability) for 50_5_96a Site B

Table S58 BBA-8,12-OMe 50_5_96b crystallographic table

CCDC Deposition Number	2342852
Empirical formula	$C_{82.5}H_{59.2}I_{12}N_{24}O_{2.1}Zn_6$
Formula weight	3335.39
Temperature/K	100(2)
Crystal system	monoclinic
Space group	P2/n
a/Å	31.6622(7)
b/Å	15.0003(2)
c/Å	34.4418(8)
α = γ /°	90
β/°	102.214(2)
Volume/Å^3	15987.6(6)
Z	4
ρ_{calc}g/cm^3	1.386
μ/mm^{-1}	19.475
F(000)	6220
Crystal size/mm^3	0.22 × 0.14 × 0.11
Radiation	Cu Kα (λ = 1.54184)
2Θ range for data collection/°	3.446 to 136.496
Index ranges	-37 ≤ h ≤ 38, -8 ≤ k ≤ 18, -40 ≤ l ≤ 41
Reflections collected	103356
Independent reflections	29162 [R_{int} = 0.0732, R_{sigma} = 0.0493]
Data/restraints/parameters	29162/1310/1363
Goodness-of-fit on F^2	1.039
Final R indexes [I>=2σ (I)]	R_1 = 0.1433, wR_2 = 0.4434
Largest diff. peak/hole / e Å^{-3}	1.58/-1.52

SQUEEZE analysis indicated two voids with volumes of 996 and 665Å^3 and electron counts of 303 and 184 respectively. This is equivalent to 6.31 and 3.83 cyclohexane molecules, or 2.33 and 1.42 BBA-8,12-OMe molecules.

Manual weighting scheme applied.

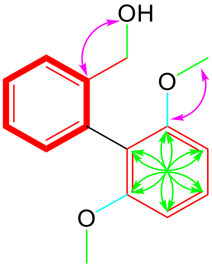

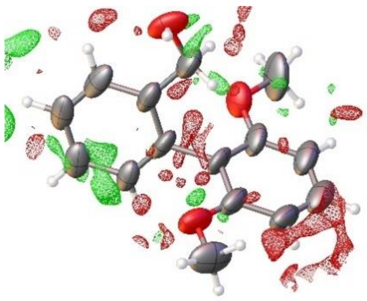

Figure S48 Geometric restraints and thermal ellipsoid (50% probability) for 50_5_96b Site A

Table S59 BBA-8,12-OMe 50_5_96c crystallographic table

CCDC Deposition Number	2342853
Empirical formula	$C_{87.95}H_{67.9}I_{12}N_{24}O_{2.58}Zn_6$
Formula weight	3417.24
Temperature/K	100(2)
Crystal system	monoclinic
Space group	P2/n
a/Å	31.4491(6)
b/Å	15.0235(2)
c/Å	34.2150(5)
α = γ /°	90
β/°	101.680(2)
Volume/Å^3	15831.0(4)
Z	4
ρ_{calc}g/cm^3	1.434
μ/mm^{-1}	19.684
F(000)	6401
Crystal size/mm^3	0.41 × 0.24 × 0.11
Radiation	Cu Kα (λ = 1.54184)
2Θ range for data collection/°	4.272 to 136.502
Index ranges	-37 ≤ h ≤ 37, -18 ≤ k ≤ 18, -40 ≤ l ≤ 41
Reflections collected	121588
Independent reflections	28909 [R_{int} = 0.0595, R_{sigma} = 0.0403]
Data/restraints/parameters	28909/719/1618
Goodness-of-fit on F^2	1.045
Final R indexes [I>=2σ (I)]	R_1 = 0.0803, wR_2 = 0.2470
Largest diff. peak/hole / e Å^{-3}	1.30/-1.00

Exchange site B was located over a two-fold symmetry axis and was modelled in PART -1. SQUEEZE analysis indicated three voids with volumes of 728, 240, and 88Å^3 and electron counts of 188, 48, and 10 respectively. This is equivalent to 3.92, 1.00, and 0.21 cyclohexane molecules, or 0.1.45, 0.37, and 0.08 BBA-8,12-OMe molecules.

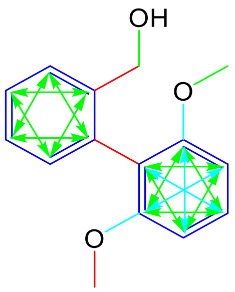

66.3%

SIMU

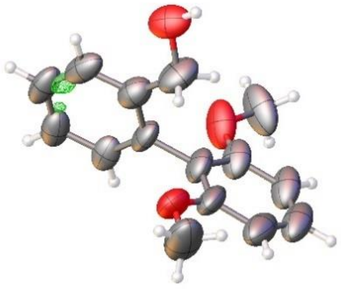

Figure S49 Geometric restraints and thermal ellipsoid (50% probability) for 50_5_96c Site A

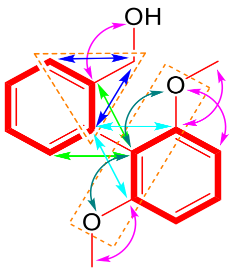

19.5%

SIMU + RIGU

DANG 0.02 [C12B – C15B]

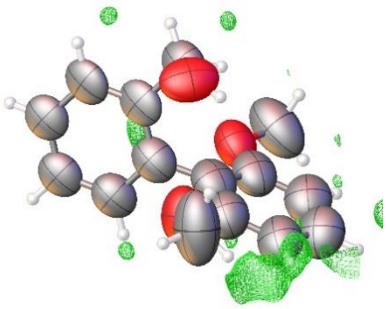

Figure S50 Geometric restraints and thermal ellipsoid (50% probability) for 50_5_96c Site B

Table S60 BBA-8,12-OMe 50_10_24a crystallographic table

CCDC Deposition Number	2342854
Empirical formula	$C_{90.62}H_{72.78}I_{12}N_{24}O_{2.67}Zn_6$
Formula weight	3455.65
Temperature/K	100(2)
Crystal system	monoclinic
Space group	P2/n
a/Å	31.5672(6)
b/Å	15.0183(2)
c/Å	34.3644(5)
α = γ /°	90
β/°	102.009(2)
Volume/Å^3	15935.1(5)
Z	4
ρ_{calc}g/cm^3	1.44
μ/mm^{-1}	19.562
F(000)	6487
Crystal size/mm^3	0.21 × 0.13 × 0.06
Radiation	Cu Kα (λ = 1.54184)
2Θ range for data collection/°	4.27 to 136.498
Index ranges	-37 ≤ h ≤ 38, -15 ≤ k ≤ 18, -41 ≤ l ≤ 36
Reflections collected	102244
Independent reflections	29077 [R_{int} = 0.0447, R_{sigma} = 0.0453]
Data/restraints/parameters	29077/881/1751
Goodness-of-fit on F^2	1.046
Final R indexes [I>=2σ (I)]	R_1 = 0.0766, wR_2 = 0.2403
Largest diff. peak/hole / e Å^{-3}	1.40/-1.03

Exchange site B was located over a two-fold symmetry axis and was modelled in PART -1. SQUEEZE analysis indicated three voids with volumes of 245, 474, and 89Å^3 and electron counts of 58, 125, and 20 respectively. This is equivalent to 1.21, 2.60, and 0.42 cyclohexane molecules, or 0.45, 0.96, and 0.15 BBA-8,12-OMe molecules

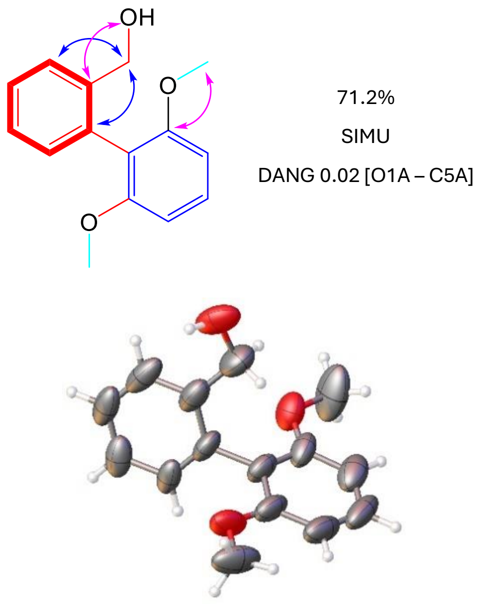

Figure S51 Geometric restraints and thermal ellipsoid (50% probability) for 50_10_24a Site A

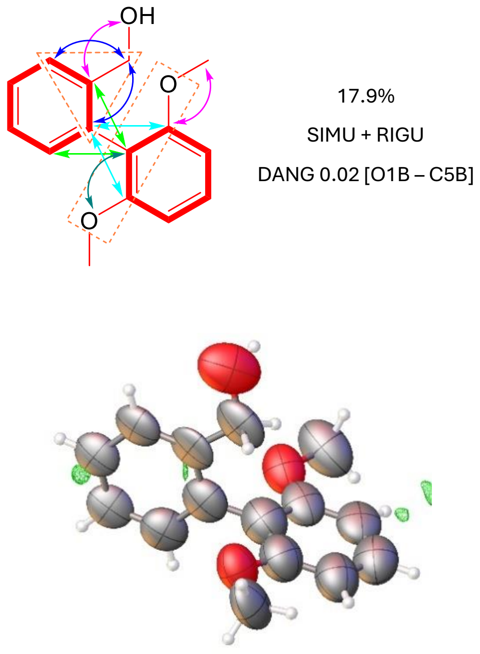

Figure S52 Geometric restraints and thermal ellipsoid (50% probability) for 50_10_24a Site B

Table S61 BBA-8,12-OMe 50_10_24b crystallographic table

CCDC Deposition Number	2342855
Empirical formula	$C_{85.35}H_{63.69}I_{12}N_{24}O_{2.36}Zn_6$
Formula weight	3378.3
Temperature/K	100.00(10)
Crystal system	monoclinic
Space group	P2/n
a/Å	31.4790(8)
b/Å	15.0233(3)
c/Å	34.2503(6)
α = γ /°	90
β/°	101.732(2)
Volume/Å^3	15859.2(6)
Z	4
ρ_{calc}g/cm^3	1.415
μ/mm^{-1}	19.641
F(000)	6315
Crystal size/mm^3	0.35 × 0.12 × 0.06
Radiation	Cu Kα (λ = 1.54184)
2Θ range for data collection/°	4.27 to 136.502
Index ranges	-37 ≤ h ≤ 37, -17 ≤ k ≤ 18, -41 ≤ l ≤ 28
Reflections collected	157903
Independent reflections	28931 [R_{int} = 0.0592, R_{sigma} = 0.0382]
Data/restraints/parameters	28931/669/1576
Goodness-of-fit on F^2	1.016
Final R indexes [I>=2σ (I)]	R_1 = 0.1149, wR_2 = 0.3533
Largest diff. peak/hole / e Å^{-3}	1.33/-1.19

Exchange site B was located over a two-fold symmetry axis and was modelled in PART -1. SQUEEZE analysis indicated three voids with volumes of 728, 426, and 86Å^3 and electron counts of 176, 86, and 22 respectively. This is equivalent to 3.67, 1.79, and 0.46 cyclohexane molecules, or 1.35, 0.66, and 0.17 BBA-8,12-OMe molecules

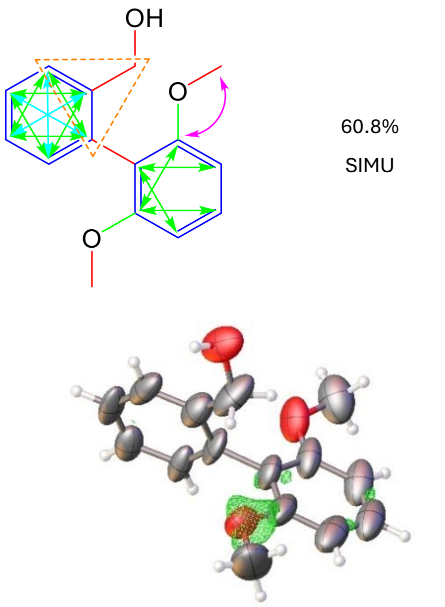

Figure S53 Geometric restraints and thermal ellipsoid (50% probability) for 50_10_24b Site A

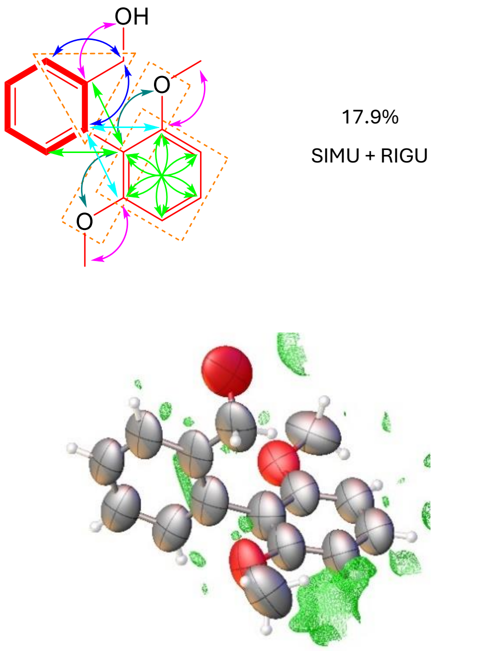

Figure S54 Geometric restraints and thermal ellipsoid (50% probability) for 50_10_24b Site B

Table S62 BBA-8,12-OMe 50_10_24c crystallographic table

CCDC Deposition Number	2342856
Empirical formula	$C_{90.91}H_{73.02}I_{12}N_{24}O_{2.74}Zn_6$
Formula weight	3460.47
Temperature/K	100(2)
Crystal system	monoclinic
Space group	P2/n
a/Å	31.5490(9)
b/Å	15.0248(3)
c/Å	34.3298(10)
α = γ /°	90
β/°	101.853(3)
Volume/Å^3	15925.9(7)
Z	4
ρ_{calc}g/cm^3	1.443
μ/mm^{-1}	19.574
F(000)	6497
Crystal size/mm^3	0.19 × 0.08 × 0.06
Radiation	Cu Kα (λ = 1.54184)
2Θ range for data collection/°	3.466 to 136.5
Index ranges	-38 ≤ h ≤ 38, -17 ≤ k ≤ 18, -35 ≤ l ≤ 41
Reflections collected	122860
Independent reflections	28986 [R_{int} = 0.0611, R_{sigma} = 0.0408]
Data/restraints/parameters	28986/739/1608
Goodness-of-fit on F^2	1.061
Final R indexes [I>=2σ (I)]	R_1 = 0.1239, wR_2 = 0.3249
Largest diff. peak/hole / e Å^{-3}	1.32/-1.70
Exchange site B was located over a two-fold symmetry axis and was modelled in PART -1. SQUEEZE analysis indicated three voids with volumes of 248, 646, and 90Å^3 and electron counts of 52, 169, and 20 respectively. This is equivalent to 1.08, 3.52, and 0.42 cyclohexane molecules, or 0.40, 1.30, and 0.15 BBA-8,12-OMe molecules	

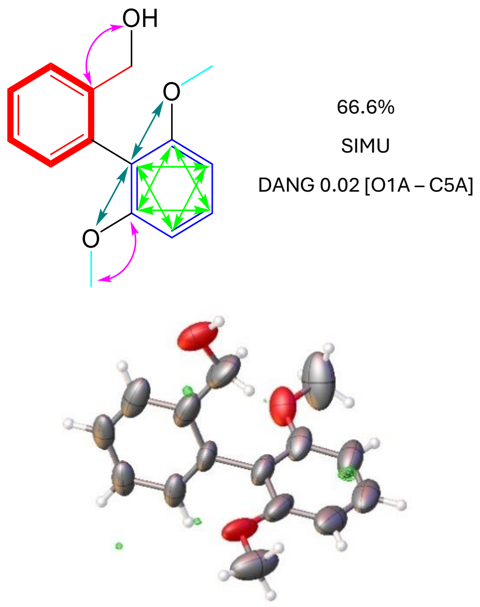

Figure S55 Geometric restraints and thermal ellipsoid (50% probability) for 50_10_24c Site A

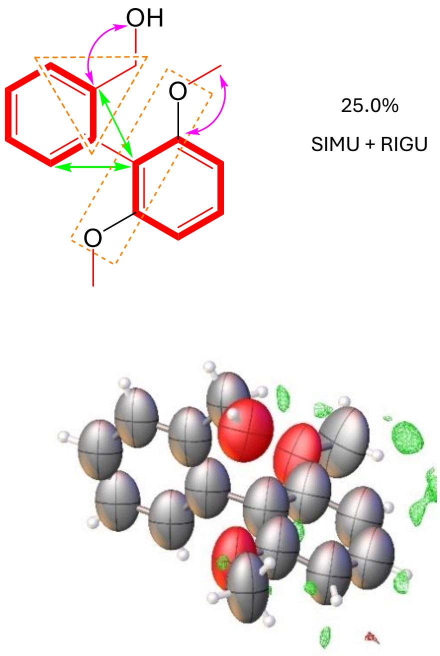

Figure S56 Geometric restraints and thermal ellipsoid (50% probability) for 50_1_24c Site B

Table S63 BBA-8,12-OMe 50_10_48a crystallographic table

CCDC Deposition Number	2342857
Empirical formula	$C_{100.98}H_{91.97}I_{12}N_{24}O_{2.88}Zn_6$
Formula weight	3602.84
Temperature/K	100(2)
Crystal system	monoclinic
Space group	P2/n
a/Å	31.5077(5)
b/Å	14.99910(10)
c/Å	34.4289(6)
α = γ /°	90
β/°	102.0860(10)
Volume/Å^3	15910.0(4)
Z	4
ρ_{calc}g/cm^3	1.504
μ/mm^{-1}	19.618
F(000)	6820
Crystal size/mm^3	0.28 × 0.17 × 0.07
Radiation	Cu Kα (λ = 1.54184)
2Θ range for data collection/°	4.274 to 136.498
Index ranges	-37 ≤ h ≤ 37, -18 ≤ k ≤ 11, -41 ≤ l ≤ 41
Reflections collected	105993
Independent reflections	29014 [R_{int} = 0.0408, R_{sigma} = 0.0360]
Data/restraints/parameters	29014/1288/1990
Goodness-of-fit on F^2	1.064
Final R indexes [I>=2σ (I)]	R_1 = 0.0705, wR_2 = 0.2089
Largest diff. peak/hole / e Å^{-3}	0.95/-0.94

Exchange site B was located over a two-fold symmetry axis and was modelled in PART -1. SQUEEZE analysis indicated one void with a volume of 172Å^3 and electron count of 32. This is equivalent to 0.67 cyclohexane molecules or 0.27 BBA-8,12-OMe molecules.

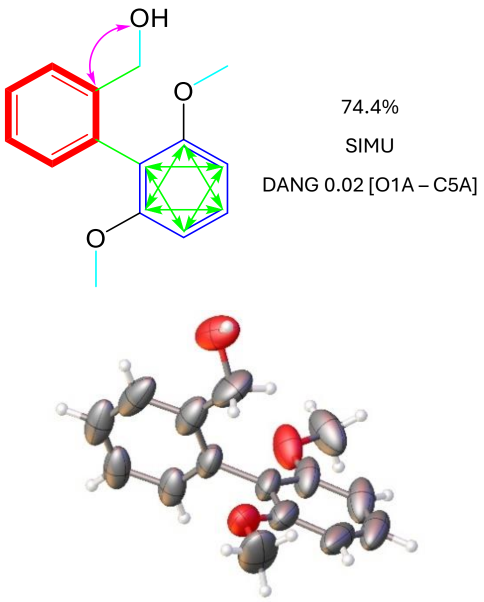

Figure S57 Geometric restraints and thermal ellipsoid (50% probability) for 50_10_48a Site A

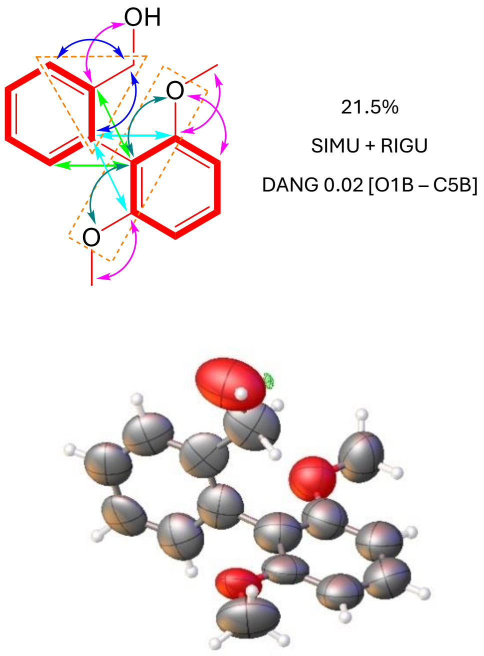

Figure S58 Geometric restraints and thermal ellipsoid (50% probability) for 50_10_48a Site B

Table S64 BBA-8,12-OMe 50_10_48b crystallographic table

CCDC Deposition Number	2342858
Empirical formula	$C_{90.17}H_{71.8}I_{12}N_{24}O_{2.69}Zn_6$
Formula weight	3449.54
Temperature/K	100(2)
Crystal system	monoclinic
Space group	P2/n
a/Å	31.5685(8)
b/Å	15.0410(3)
c/Å	34.3133(9)
α = γ /°	90
β/°	101.955(2)
Volume/Å³	15939.3(7)
Z	4
ρ_{calc}g/cm³	1.437
μ/mm⁻¹	19.556
F(000)	6473
Crystal size/mm³	0.23 × 0.15 × 0.07
Radiation	Cu Kα (λ = 1.54184)
2Θ range for data collection/°	4.27 to 136.498
Index ranges	-37 ≤ h ≤ 37, -18 ≤ k ≤ 14, -41 ≤ l ≤ 41
Reflections collected	96418
Independent reflections	28902 [R_{int} = 0.0498, R_{sigma} = 0.0356]
Data/restraints/parameters	28902/903/1713
Goodness-of-fit on F²	1.091
Final R indexes [I>=2σ (I)]	R_1 = 0.1219, wR_2 = 0.3321
Largest diff. peak/hole / e Å⁻³	1.42/-1.24

Exchange site B was located over a two-fold symmetry axis and was modelled in PART -1. SQUEEZE analysis indicated three voids with volumes of 483, 420, and 83Å³ and electron counts of 128, 94, and 22 respectively. This is equivalent to 2.67, 1.96, and 0.46 cyclohexane molecules, or 0.98, 0.72, and 0.17 BBA-8,12-OMe molecules

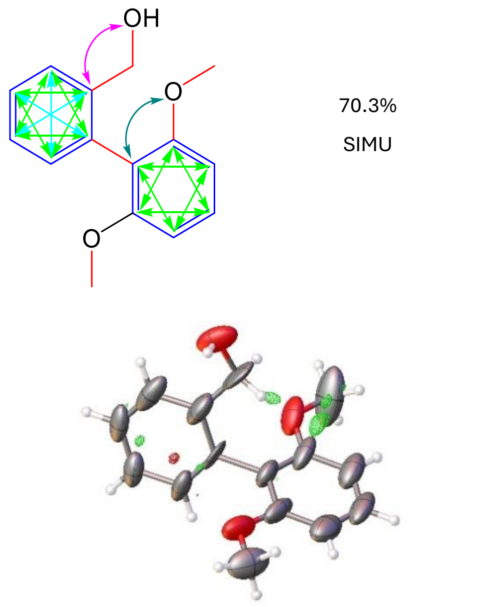

Figure S59 Geometric restraints and thermal ellipsoid (50% probability) for 50_10_48b Site A

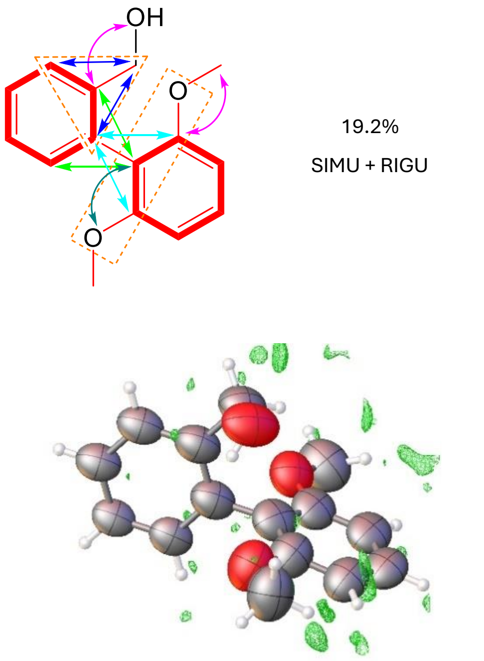

Figure S60 Geometric restraints and thermal ellipsoid (50% probability) for 50_10_48b Site B

Table S65 BBA-8,12-OMe 50_10_48c crystallographic table

CCDC Deposition Number	2342859
Empirical formula	$C_{36}H_{24}I_6N_{12}Zn_3$
Formula weight	1582.18
Temperature/K	100(2)
Crystal system	monoclinic
Space group	C2/c
a/Å	34.3804(16)
b/Å	15.0439(5)
c/Å	30.3200(17)
α = γ /°	90
β/°	101.107(5)
Volume/Å^3	15388.2(13)
Z	8
ρ_{calc}g/cm^3	1.366
μ/mm^{-1}	20.192
F(000)	5856
Crystal size/mm^3	0.26 × 0.12 × 0.1
Radiation	Cu Kα (λ = 1.54184)
2Θ range for data collection/°	5.942 to 136.496
Index ranges	-41 ≤ h ≤ 38, -17 ≤ k ≤ 18, -36 ≤ l ≤ 33
Reflections collected	51653
Independent reflections	13991 [R_{int} = 0.0607, R_{sigma} = 0.0510]
Data/restraints/parameters	13991/95/634
Goodness-of-fit on F^2	1.116
Final R indexes [I>=2σ (I)]	R_1 = 0.1191, wR_2 = 0.3901
Largest diff. peak/hole / e Å^{-3}	1.90/-1.12

No guests or solvent were identified from the electron density map, SQUEEZE analysis indicated one void with a volume of 867Å^3 and electron count of 210. This is equivalent to 4.38 cyclohexane molecules or 1.62 BBA-8,12-OMe molecules.

Manual weighting scheme applied.

Table S66 BBA-8,12-OMe 50_10_96a crystallographic table

CCDC Deposition Number	2342860
Empirical formula	$C_{92.12}H_{75.43}I_{12}N_{24}O_{2.75}Zn_6$
Formula weight	3477.6
Temperature/K	100(2)
Crystal system	monoclinic
Space group	P2/n
a/Å	31.6878(7)
b/Å	14.9984(3)
c/Å	34.4068(8)
α = γ /°	90
β/°	102.171(2)
Volume/Å^3	15984.8(6)
Z	4
ρ_{calc}g/cm^3	1.445
μ/mm^{-1}	19.505
F(000)	6536
Crystal size/mm^3	0.23 × 0.17 × 0.07
Radiation	Cu Kα (λ = 1.54184)
2Θ range for data collection/°	4.266 to 136.502
Index ranges	-38 ≤ h ≤ 38, -7 ≤ k ≤ 18, -41 ≤ l ≤ 41
Reflections collected	136060
Independent reflections	29217 [R_{int} = 0.0483, R_{sigma} = 0.0328]
Data/restraints/parameters	29217/803/1640
Goodness-of-fit on F^2	1.095
Final R indexes [I>=2σ (I)]	R_1 = 0.0874, wR_2 = 0.2576
Largest diff. peak/hole / e Å^{-3}	1.51/-0.88
Exchange site B was located over a two-fold symmetry axis and was modelled in PART -1. SQUEEZE analysis indicated three voids with volumes of 660, 35, and 88Å^3 and electron counts of 162, 5, and 14 respectively. This is equivalent to 3.38, 0.10, and 0.29 cyclohexane molecules, or 1.24, 0.04, and 0.11 BBA-8,12-OMe molecules	

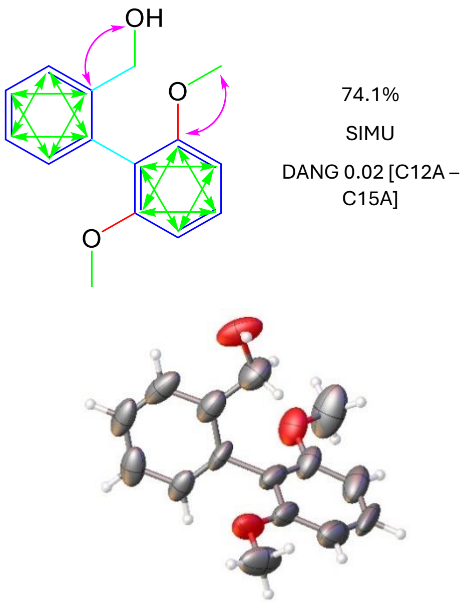

Figure S61 Geometric restraints and thermal ellipsoid (50% probability) for 50_10_96a Site A

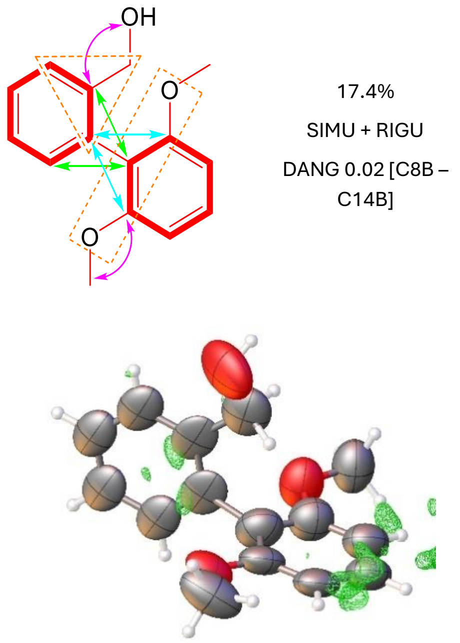

Figure S62 Geometric restraints and thermal ellipsoid (50% probability) for 50_10_96a Site B

Table S67 BBA-8,12-OMe 50_10_96b crystallographic table

CCDC Deposition Number	2342861
Empirical formula	$C_{88.11}H_{68.2}I_{12}N_{24}O_{2.57}Zn_6$
Formula weight	3419.3
Temperature/K	100(2)
Crystal system	monoclinic
Space group	P2/n
a/Å	31.5313(6)
b/Å	15.0299(2)
c/Å	34.2616(5)
α = γ /°	90
β/°	101.863(2)
Volume/Å^3	15890.2(5)
Z	4
ρ_{calc}g/cm^3	1.429
μ/mm^{-1}	19.611
F(000)	6406
Crystal size/mm^3	0.26 × 0.15 × 0.1
Radiation	Cu Kα (λ = 1.54184)
2Θ range for data collection/°	3.47 to 136.504
Index ranges	-37 ≤ h ≤ 37, -18 ≤ k ≤ 17, -41 ≤ l ≤ 35
Reflections collected	103014
Independent reflections	28957 [R_{int} = 0.0370, R_{sigma} = 0.0351]
Data/restraints/parameters	28957/718/1617
Goodness-of-fit on F^2	1.035
Final R indexes [I>=2σ (I)]	R_1 = 0.0796, wR_2 = 0.2610
Largest diff. peak/hole / e Å^{-3}	1.44/-1.31
Exchange site B was located over a two-fold symmetry axis and was modelled in PART -1. SQUEEZE analysis indicated three voids with volumes of 624, 237, and 72Å^3 and electron counts of 143, 37, and 19 respectively. This is equivalent to 2.97, 0.77, and 0.40 cyclohexane molecules, or 1.10, 0.28, and 0.15 BBA-8,12-OMe molecules	

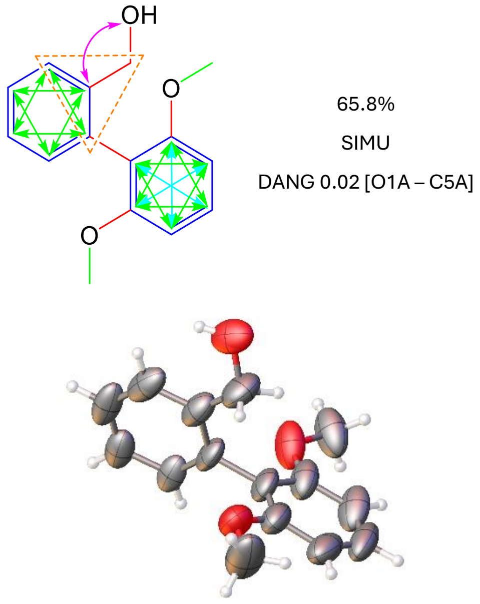

Figure S63 Geometric restraints and thermal ellipsoid (50% probability) for 50_10_96b Site A

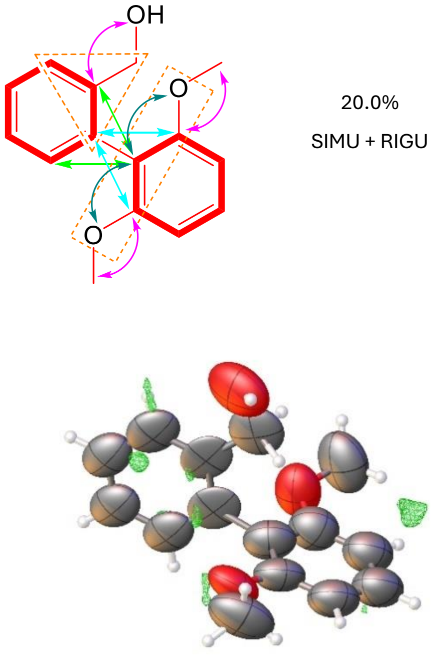

Figure S64 Geometric restraints and thermal ellipsoid (50% probability) for 50_10_96b Site B

Table S68 BBA-8,12-OMe 50_10_96c crystallographic table

CCDC Deposition Number	2342862
Empirical formula	$C_{95.66}H_{80.05}I_{12.07}N_{24}O_{3.27}Zn_{6.03}$
Formula weight	3543.84
Temperature/K	100.00(10)
Crystal system	monoclinic
Space group	P2/n
a/Å	31.6222(19)
b/Å	14.9929(6)
c/Å	34.405(2)
α = γ /°	90
β/°	102.192(6)
Volume/Å³	15943.7(16)
Z	4
ρ_{calc}g/cm³	1.476
μ/mm⁻¹	19.675
F(000)	6675
Crystal size/mm³	0.27 × 0.17 × 0.12
Radiation	Cu Kα (λ = 1.54184)
2Θ range for data collection/°	4.272 to 136.502
Index ranges	-37 ≤ h ≤ 38, -8 ≤ k ≤ 17, -41 ≤ l ≤ 41
Reflections collected	100270
Independent reflections	28894 [R_{int} = 0.0712, R_{sigma} = 0.0462]
Data/restraints/parameters	28894/823/1609
Goodness-of-fit on F²	1.105
Final R indexes [I>=2σ (I)]	R_1 = 0.1477, wR_2 = 0.3504
Largest diff. peak/hole / e Å⁻³	1.41/-1.89
Exchange site B was located over a two-fold symmetry axis and was modelled in PART -1. SQUEEZE analysis indicated three voids with volumes of 430, 507, and 88Å³ and electron counts of 114, 129, and 19 respectively. This is equivalent to 2.38, 2.69, and 0.40 cyclohexane molecules, or 0.88, 0.99, and 0.15 BBA-8,12-OMe molecules	

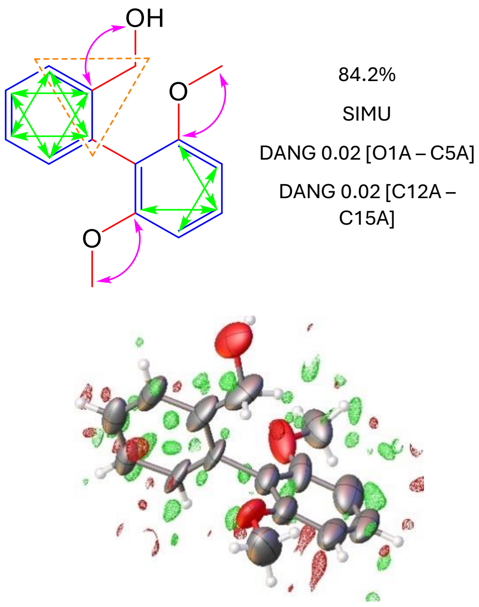

Figure S65 Geometric restraints and thermal ellipsoid (50% probability) for 50_10_96c Site A

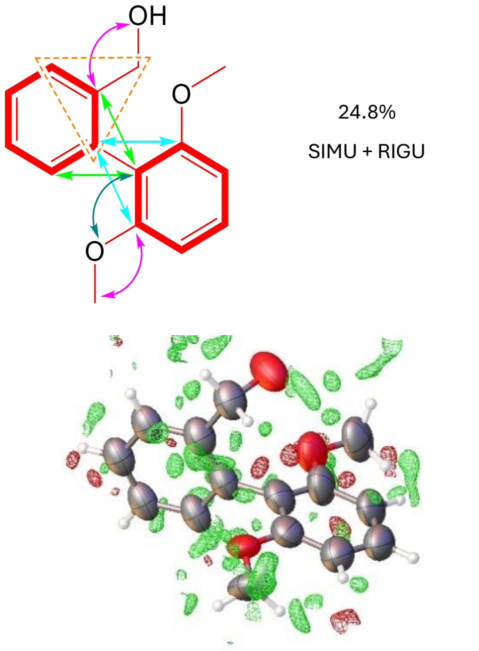

Figure S66 Geometric restraints and thermal ellipsoid (50% probability) for 50_10_96c Site B

S6. Guest Exchange Rates Full Tabulation

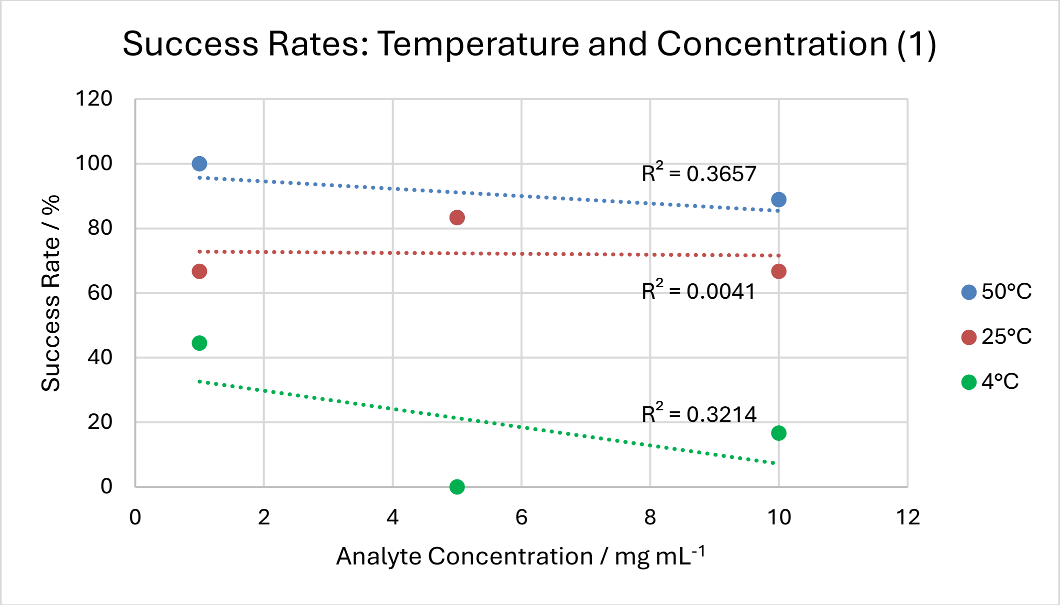

Figure S67 Exchange temperature and analyte concentration influence on exchange success (1)

Table S69 Linear regression statistics for exchange success with temperature and concentration (1)

	Standard Error	t-stat	P-value
50 °C	9.559669	-0.75932	0.586554
25 °C	13.57493	-0.06415	0.959217
4 °C	26.15397	-0.68819	0.616273

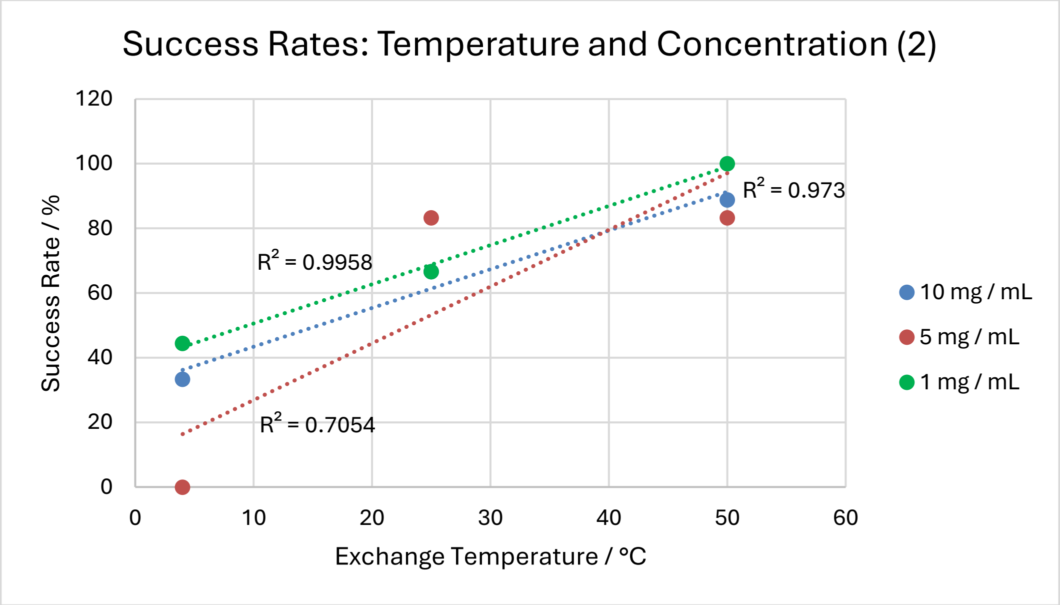

Figure S68 Exchange temperature and analyte concentration influence on exchange success (2)

Table S70 Linear regression statistics for exchange success with temperature and concentration (2)

	Standard Error	t-stat	P-value
10 mg mL^{-1}	6.503901	5.997909	0.105173
5 mg mL^{-1}	36.93102	1.547299	0.365268
1 mg mL^{-1}	2.555965	15.44022	0.041174

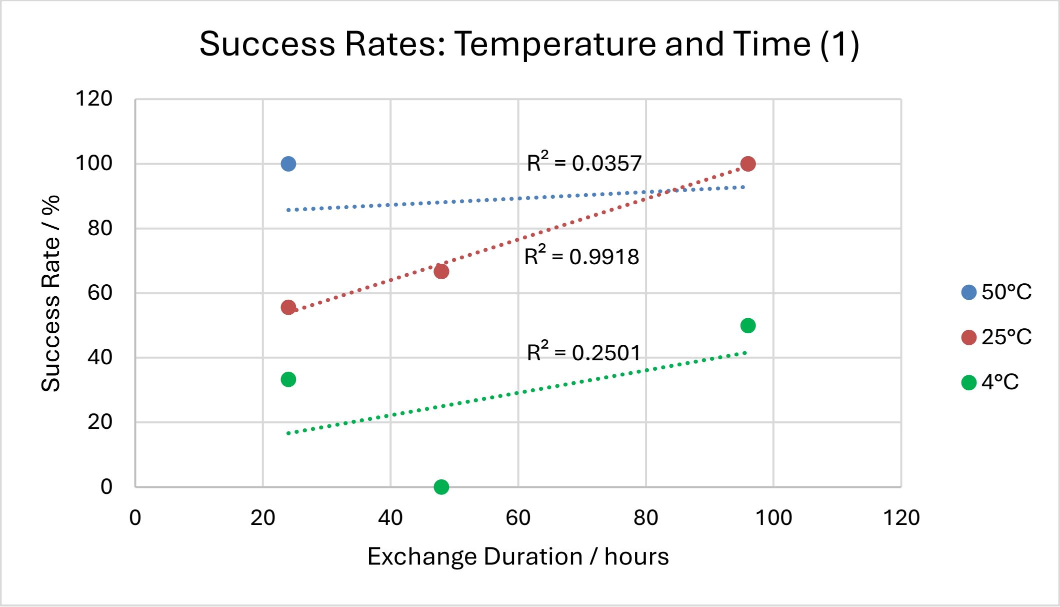

Figure S69 Exchange temperature and exchange duration influence on exchange success (1)

Table S71 Linear regression statistics for exchange success with temperature and duration (1)

	Standard Error	t-stat	P-value
50 °C	26.72345	0.19245	0.878962
25 °C	2.969272	10.96966	0.057875
4 °C	31.1787	0.577449	0.666619

Experiments conducted at 25°C exhibited a positive correlation with respect to time, this may suggest that systems with lower kinetic energy may rely more heavily on longer exchange durations for successful diffusion and settling within the pores.

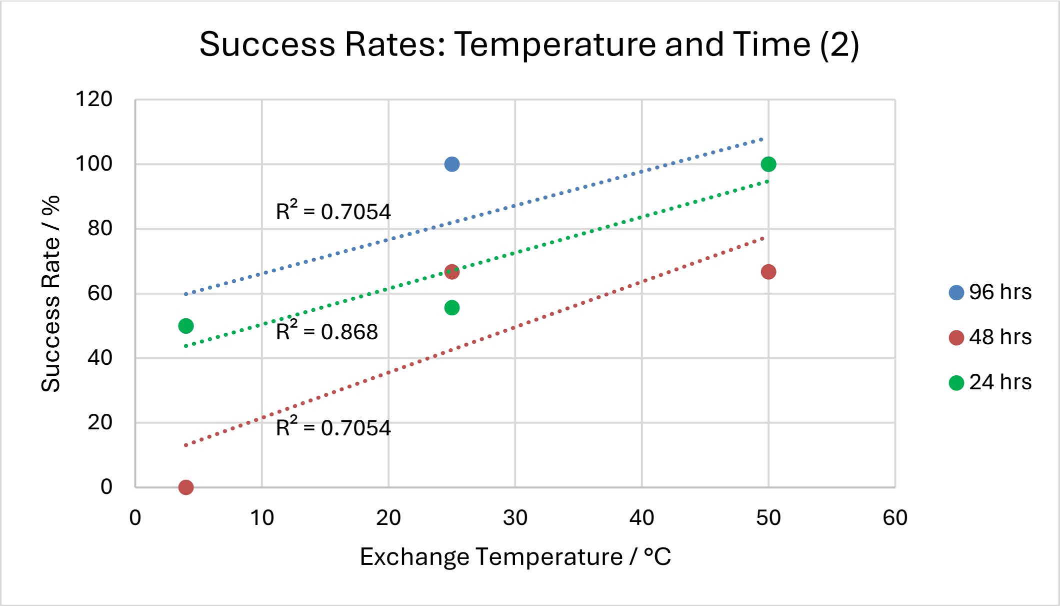

Figure S70 Exchange temperature and exchange duration influence on exchange success (2)

Table S72 Linear regression statistics for exchange success with temperature and duration (2)

	Standard Error	t-stat	P-value
96 hours	22.1595	1.547299	0.365268
48 hours	29.54748	1.547299	0.365268
24 hours	14.07997	2.564405	0.236705

Longer exchange durations of 96 hours in combination with variable temperature all displayed successful results but a clear step in guest exchange rates was observed for 25°C and above as 100% exchange success was achieved. Further studies could investigate a more gradual increase in temperature to better understand the role the factor plays.

Another feature identified for 48-hour exchange experiments was the significant decline in success when combined with the lowest exchange temperature (4°C). These two variables were identified as the least successful from the initial analysis but is highlighted more clearly here in comparison to 25°C and 50°C conditions.

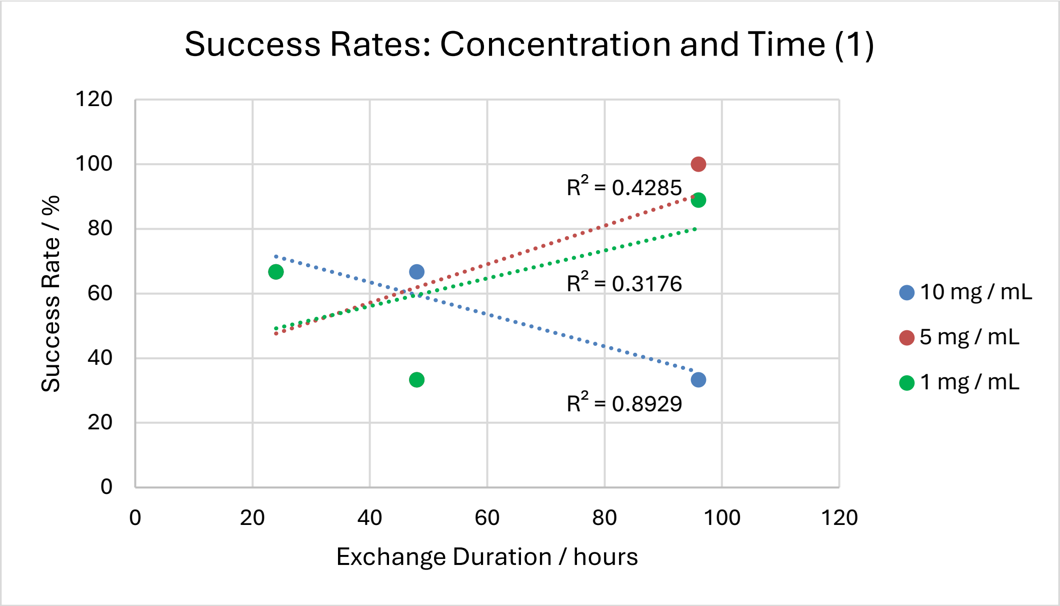

Figure S71 Exchange concentration and exchange duration influence on exchange success (1)

Table S73 Linear regression statistics for exchange success with concentration and duration (1)

	Standard Error	t-stat	P-value
96 hours	8.91049	-2.88675	0.212296
48 hours	35.63929	0.865874	0.545684
24 hours	32.67001	0.682203	0.618868

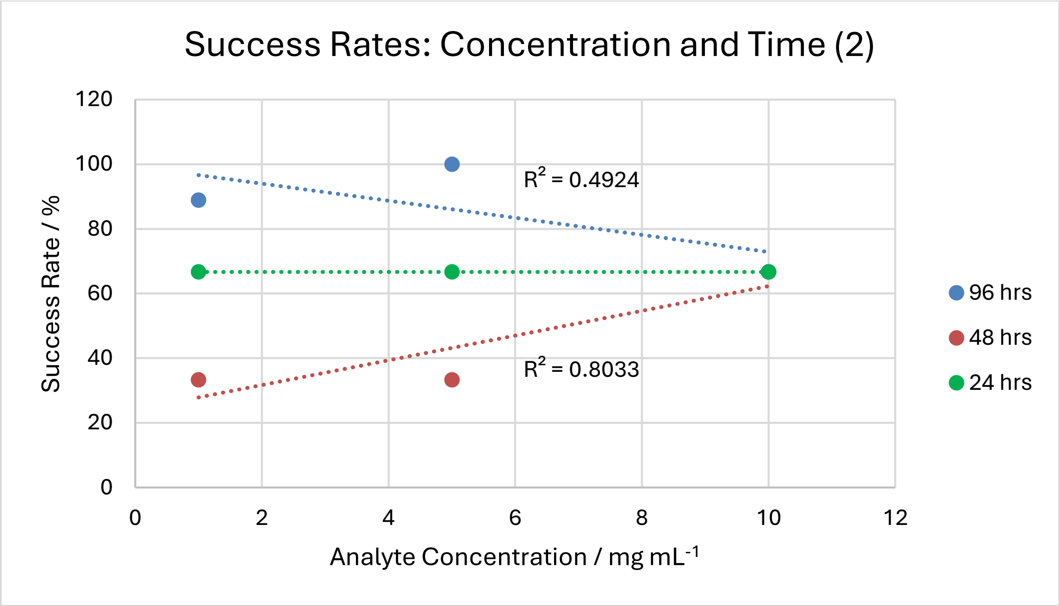

Figure S72 Exchange concentration and exchange duration influence on exchange success (2)

Table S74 Linear regression statistics for exchange success with concentration and duration (2)

	Standard Error	t-stat	P-value
10 mg mL^{-1}	17.09949	-0.98489	0.504846
5 mg mL^{-1}	12.07385	2.020726	0.29255
1 mg mL^{-1}	0	-	-

Guest exchange over 48 hours displayed no difference in success for 1 mg mL^{-1} or 5 mg mL^{-1} with 33.33% of experiments resulting in guest modelling. However, increasing the concentration to 10 mg mL^{-1} doubled the number of successes (66.67%) which suggests a significantly stronger concentration gradient can have a positive influence on the result.

S7. Crystallographic Analysis of P2/n and C2/c [48k] Forms

To rationalise the differences between P2/n and C2/c [48k] forms, more detailed consideration of their respective secondary site locations and interactions is required. For P2/n, the weaker secondary exchange resides over a 2-fold symmetry axis and maximises interaction with the host framework through oxygen ··· hydrogen (O ··· H) contacts. Practically, this requires modelling the site in PART -1 with the application of considerable restraints and constraints to achieve a stable structure.

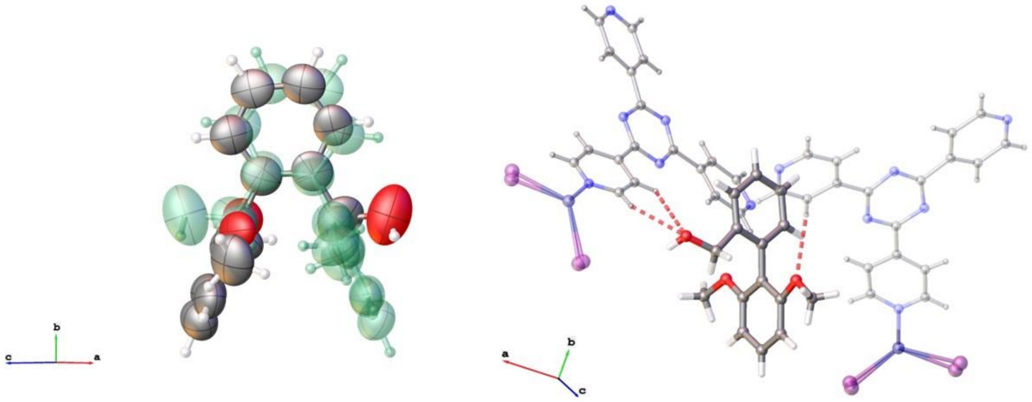

Figure S73 P2/n site B 2-fold disorder (left) and example O ··· H interactions with host (right)

The secondary site occupied for C2/c [48k] structures is similar in location to that observed for P2/n, however it has a slight adjustment in orientation that prioritises interaction with a neighbouring guest molecule through aromatic ··· aromatic (Ar ··· Ar) and oxygen ··· aromatic (O ··· Ar) contacts.

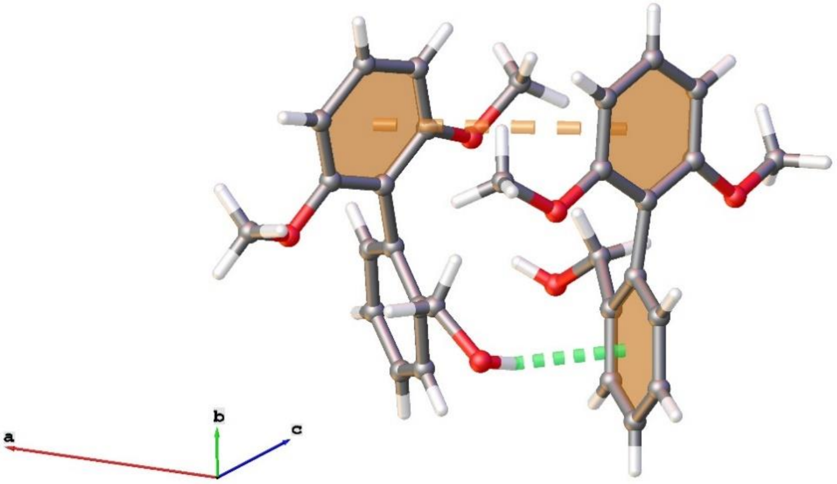

Figure S74 C2/c [48k] site B / C guest-guest Ar ··· Ar and O ··· Ar contacts

This subtle change of guest location and interaction results in expansion of the unit cell, shifting the location of the 2-fold symmetry axis. This enables two crystallographically unique molecules to be modelled on the site without disorder and consequently requires less intervention with restraints and constraints for modelling.

Although the secondary (and tertiary) site adopted in C2/c [48k] enables guests to be modelled more easily, the reliance on nearby guest molecules for contact is much less reliable than interaction with the host framework. This is evidenced by 80% of P2/n structures possessing a secondary site, but only 40% of C2/c [48k] structures. This highlights a key difficulty of small molecule characterisation inside larger networks: although there may be sufficient diffraction from guest molecules to violate systematic absences exhibited by the host symmetry, this does not mean that there is sufficient information for full modelling of these guests.

It is possible to combat this with batch refinement strategies, commonly used for variable temperature or pressure experiments, where .hkl files from better resolved structures are used as the basis for new models. However, this of course requires already obtaining high-quality data of the target guest and is therefore only applicable in these larger systematic studies.

S8. Crystallographic Form Occurrence

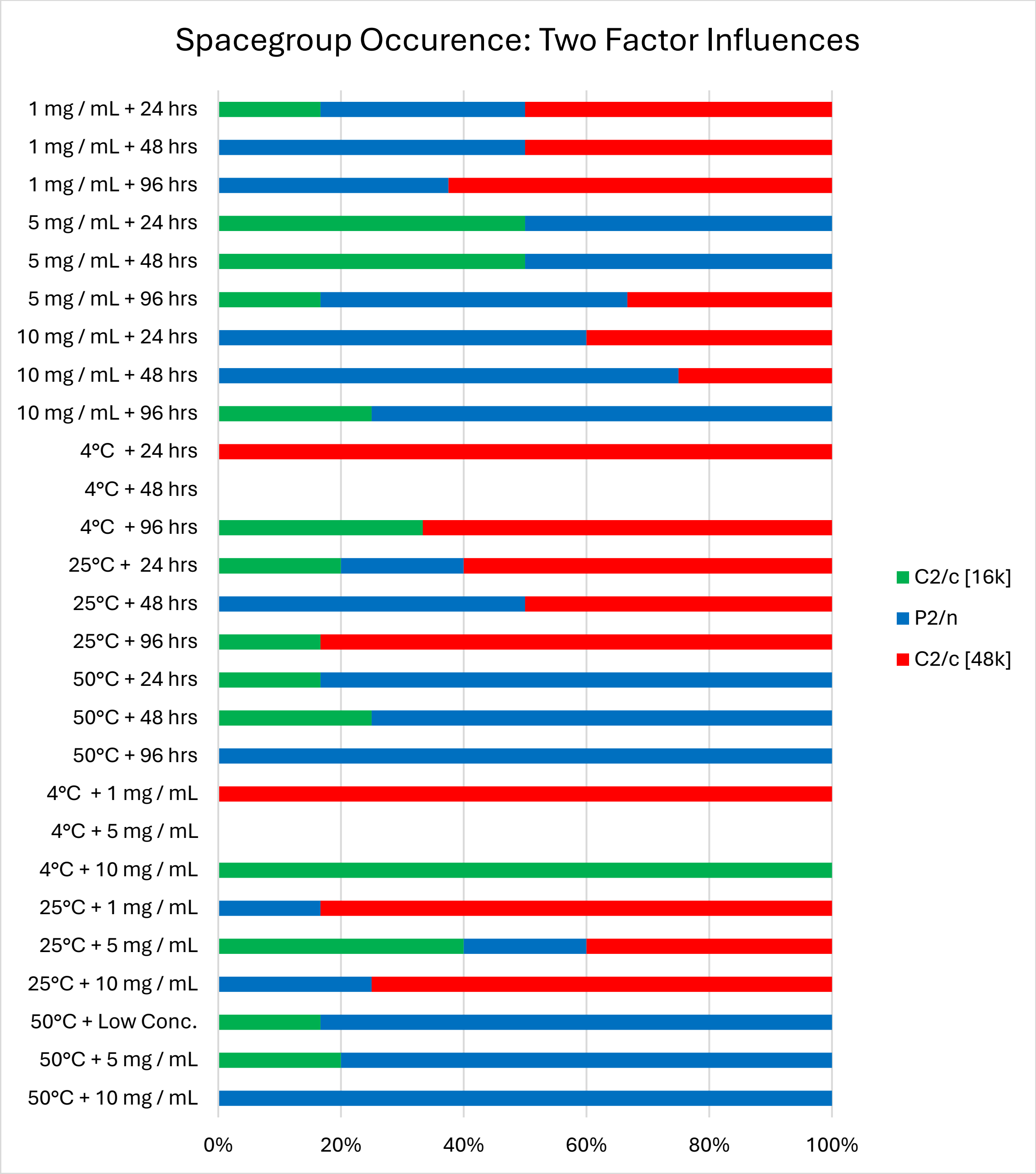

Figure S75 Two factor influence on crystallographic form occurrence for BBA-8,12-OMe (1).

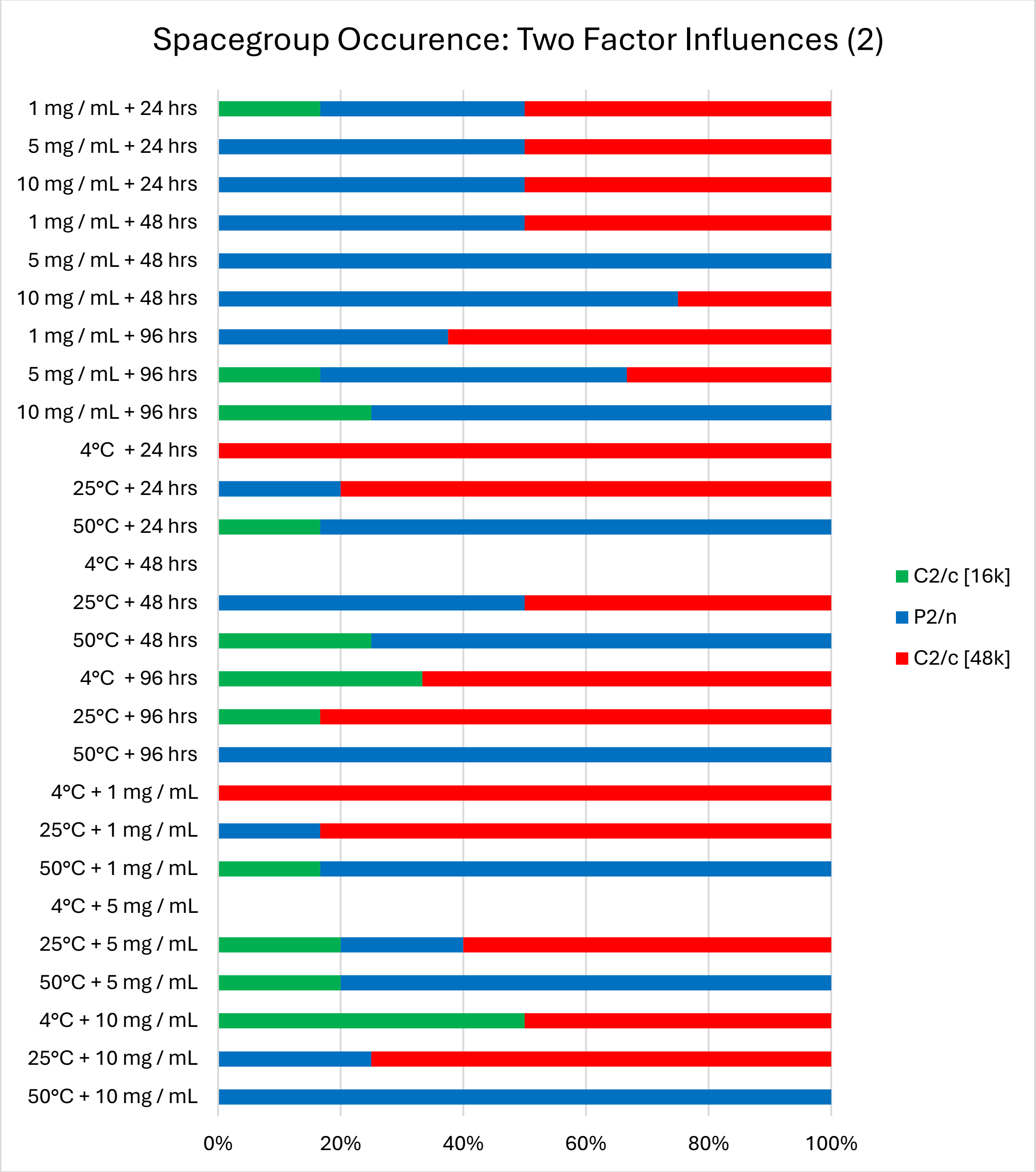

Figure S76 Two factor influence on crystallographic form occurrence for BBA-8,12-OMe (2).

S9. Crystallographic Form Interaction Analysis

Interaction analysis was conducted when model refinement had been finalised. To identify the interactions, the Olex2, PLATON, and Mercury programmes were utilised. (Dolomanov *et al.*, 2009; Spek, 2003; Groom *et al.*, 2016)

Reliable analysis and comparison of interactions within confined pores relies on a consistent set of parameters to define contacts. The cut-off criteria for the contacts analysed herein have been derived from accepted standards commonly used in small molecule crystallography, as defined in PLATON. These have also been corroborated with frequency distributions of close contacts found within the Cambridge Structural Database. These criteria are summarised below in Table S69.

Table S75 Intermolecular interaction criteria

Interaction	Distance / Å	Angle / °	Dihedral Angle Between Planes I and J / °	Angle Cg(I) → Cg(J) or Cg(I) → Me vector and normal to plane I / °
X ⋯ Y	< 3.6	> 60	-	-
X ⋯ I – Zn*	< 3.6	> 60	-	-
X ⋯ Cg	< 4.0	> 90	-	-
H ⋯ Cg	< 3.6	> 90	-	-
Cg ⋯ Cg	< 6.0	< 20	< 20	< 60

* Interactions only accounted for with the major component of ZnI_2 disorder. Where ‘Planes I and J’ refer to the planes defined by respective aromatic rings, and Cg(I) / Cg(J) refer to the centre of gravity for rings I and J.

Table S76 C2/c [16k] atom interactions (50_1_24b)

Type 1	Type 2	Comp. 1	Comp. 2	Symm. Op. 1	Symm. Op. 2	Length / Å	Angle / °
Host - Guest	O ⋯ H	O1A	H328	x,y,z	-1/2+x,1/2+y,z	2.42(3)	105(1)
		O1A	H329	x,y,z	-1/2+x,1/2+y,z	2.29(3)	148(1)
	H ⋯ I	H1A	I301	x,y,z	1/2-x,1/2+y,1/2-z	2.51(3)	113(1)
		H2A	I301	x,y,z	1/2-x,1/2+y,1/2-z	2.22(2)	124(1)
		H3A	I304	x,y,z	x,-1+y,z	3.11(6)	140(2)
Guest - Guest	O ⋯ H	O3A	H13B	x,y,z	-x,y,1/2-z	2.56(3)	118(1)

Table S77 C2/c [16k] ring interactions (50_1_24b)

Type 1	Type 2	Comp. 1	Comp. 2	Symm. Op. 1	Symm. Op. 2	Cg – Cg / Å	α / °	Slippage / Å
Guest - Guest	Ar ··· Ar	C1A - C6A Cen.	C1A - C6A Cen.	x,y,z	-x,y,1/2-z	5.5397(5)	11.6(18)	4.252(7)
		C7A - C12A Cen.	C7A - C12A Cen.	x,y,z	-x,y,1/2-z	5.1928(5)	13.9(7)	3.331(9)

Table S78 P2/n atom interactions (50_10_48a)

Type 1	Type 2	Comp. 1	Comp. 2	Symm. Op. 1	Symm. Op. 2	Length / Å	Angle / °
Host - Guest	O ··· H	O1A	H340	x,y,z	1/2-x,+y,1/2-z	2.740(16)	95.6(9)
		O1A	H341	x,y,z	1/2-x,+y,1/2-z	2.450(16)	138.4(9)
		O2A	H333	x,y,z	x,y,z	2.820(12)	126.3(6)
		O3A	H350	x,y,z	1/2-x,+y,1/2-z	2.720(14)	108.8(5)
		O3A	H351	x,y,z	1/2-x,+y,1/2-z	2.922(9)	140.6(5)
		O1B	H366	x,y,z	1/2+x,1-y,-1/2+z	3.08(5)	146.7(14)
		O1B	H301	x,y,z	3/2-x,+y,1/2-z	2.56(5)	133.1(14)
		O1B	H302	x,y,z	3/2-x,+y,1/2-z	2.88(6)	117.7(11)
		O2B	H310	x,y,z	3/2-x,+y,1/2-z	3.18(4)	107.3(9)
		O2B	H311	x,y,z	3/2-x,+y,1/2-z	2.59(4)	132.0(12)
		O3B	H302	x,y,z	x,y,z	2.69(6)	95(2)
		O3B	H310	x,y,z	x,y,z	2.74(4)	134.0(19)
	C-H ··· N	H14A	N308	x,y,z	x,y,z	2.852(6)	135.4(10)
	H ··· I	H3B	I303	x,y,z	3/2-x,y,1/2-z	3.069(4)	137.93(6)
Guest - Guest	H ··· Ar	H14B	C7A - C12A Cen.	x,y,z	1/2-x,+y,1/2-z	3.00(5)	147.2(11)
	O ··· H	O2A	H13B	x,y,z	1/2-x,+y,1/2-z	2.530(19)	119.2(5)
	O ··· Ar	O1A	C1A - C6A Cen.	x,y,z	1/2-x,+y,1/2-z	3.376(8)	118.7(9)

Table S79 P2/n ring interactions (50_10_48a)

Type 1	Type 2	Comp. 1	Comp. 2	Symm. Op. 1	Symm. Op. 2	Cg – Cg / Å	α / °	Slippage / Å
Guest - Guest	Ar ··· Ar	C1A - C6A Cen.	C1A - C6A Cen.	x,y,z	1/2-x,+y,1/2-z	5.756(5)	6.9(4)	4.360
		C7A - C12A Cen.	C7A - C12A Cen.	x,y,z	1/2-x,+y,1/2-z	5.205(6)	15.3(4)	3.457

Table S80 C2/c [48k] atom interactions (25_1_96c)

Type 1	Type 2	Comp. 1	Comp. 2	Symm. Op. 1	Symm. Op. 2	Length / Å	Angle / °
Host - Guest	H ··· Ar	C1C - C6C Cen.	H376	x,y,z	1/2-x,-1/2+y,1/2-z	2.50(2)	126(3)
	O ··· H	O1A	H317	x,y,z	-x,1-y,-z	2.77(2)	95.5(17)
		O1A	H318	x,y,z	-x,1-y,-z	2.48(1)	143(1)
		O2A	H313	x,y,z	-x,1-y,-z	2.89(1)	122(2)
		O3A	H320	x,y,z	x,1-y,1/2+z	2.90(2)	145(1)
		O1B	H350	x,y,z	x,y,z	2.33(2)	149(1)
		O1B	H351	x,y,z	x,y,z	2.57(2)	158(1)
		O1C	H386	x,y,z	1/2-x,1/2+y,1/2-z	2.35(4)	131(2)
		O1C	H387	x,y,z	1/2-x,1/2+y,1/2-z	2.61(5)	114(2)
		O3C	H376	x,y,z	1/2-x,1/2+y,1/2-z	2.86(4)	124(2)
	C-H ··· N	H15C	N309	x,y,z	x,1-y,1/2+z	2.75(2)	154(1)
		H14G	N331	x,y,z	-x,1-y,-z	2.87(2)	161(2)
Guest - Guest	H ··· Ar	H1BA	C7C - C12C Cen.	x,y,z	x,y,z	2.61(3)	131(4)
		H1CA	C7B - C12B Cen.	x,y,z	x,y,z	2.37(4)	160(3)
	O ··· H	O3A	H13B	x,y,z	-x,y,1/2-z	2.49(2)	115(1)
	O ··· Ar	O1B	C1C - C6C Cen.	x,y,z	x,y,z	3.189(2)	114(5)
		O1C	C1B - C6B Cen.	x,y,z	x,y,z	3.081(2)	137(4)

Table S81 C2/c [48k] ring interactions (25_1_96c)

Type 1	Type 2	Comp. 1	Comp. 2	Symm. Op. 1	Symm. Op. 2	Cg – Cg / Å	α / °	Slippage / Å
Guest - Guest	Ar ··· Ar	C1A - C6A Cen.	C1A - C6A Cen.	x,y,z	-x,y,1/2-z	5.5533(4)	9.0(12)	4.22(5)
		C7A - C12A Cen.	C7A - C12A Cen.	x,y,z	-x,y,1/2-z	5.2739(3)	14.2(6)	3.520(6)
		C1B - C6B Cen.	C1C - C6C Cen.	x,y,z	x,y,z	5.22(5)	14(3)	4.05(13)
		C7B - C12B Cen.	C7C - C12C Cen.	x,y,z	x,y,z	5.33(5)	11(2)	3.46(10)

S10. BBA-8,12-OMe Analyte Grades Summary

Table S82 BBA-8,12-OMe exchange site and average grade full tabulation.

Dataset	Site A	Site B	Site C	Average
4_1_24a	5.76	-	-	-
4_1_24b	-	-	-	-
4_1_24c	4.82	11.58	-	8.20
4_1_48a	-	-	-	-
4_1_48b	-	-	-	-
4_1_48c	-	-	-	-
4_1_96a	4.34	-	-	-
4_1_96b	-	-	-	-
4_1_96c	4.16	10.39	-	7.28
4_5_48a	-	-	-	-
4_5_48b	-	-	-	-
4_5_48c	-	-	-	-
4_10_24a	-	-	-	-
4_10_24b	-	-	-	-
4_10_24c	-	-	-	-
4_10_96a	-	-	-	-
4_10_96b	12.37	-	-	-
4_10_96c	-	-	-	-
25_1_24a	4.21	-	-	-
25_1_24b	-	-	-	-
25_1_24c	-	-	-	-
25_1_48a	5.32	-	-	-
25_1_48b	-	-	-	-
25_1_48c	5.26	11.00	-	8.13
25_1_96a	3.53	-	-	-
25_1_96b	4.68	-	-	-
25_1_96c	8.03	10.08	9.03	9.04
25_5_24a	8.68	-	-	-
25_5_24b	-	-	-	-
25_5_24c	4.26	-	-	-
25_5_96a	4.66	-	-	-
25_5_96b	5.00	10.34	-	7.67
25_5_96c	11.61	-	-	-
25_10_24a	7.08	-	-	-
25_10_24b	6.13	-	-	-
25_10_24c	-	-	-	-
25_10_48a	5.32	12.34	-	8.46
25_10_48b	-	-	-	-
25_10_48c	3.13	9.34	-	6.24
50_1_24a	4.58	12.34	-	8.46
50_1_24b	8.55	-	-	-
50_1_24c	4.16	12.74	-	8.45
50_1_96a	7.03	13.00	-	10.01

Table S82 (cont.)

50_1_96b	7.03	12.68	-	9.86
50_1_96c	3.76	-	-	-
50_5_48a	8.97	-	-	-
50_5_48b	4.42	10.53	-	7.47
50_5_48c	-	-	-	-
50_5_96a	5.34	12.24	-	8.79
50_5_96b	5.79	-	-	-
50_5_96c	3.61	12.50	-	8.05
50_10_24a	5.87	12.18	-	9.03
50_10_24b	4.37	11.39	-	7.88
50_10_24c	5.55	10.50	-	8.03
50_10_48a	4.89	12.00	-	8.45
50_10_48b	3.42	11.37	-	7.39
50_10_48c	-	-	-	-
50_10_96a	3.00	11.37	-	7.18
50_10_96b	4.53	12.63	-	8.58
50_10_96c	2.92	10.61	-	6.76

S11. Analyte Grading: Single Factor Plots

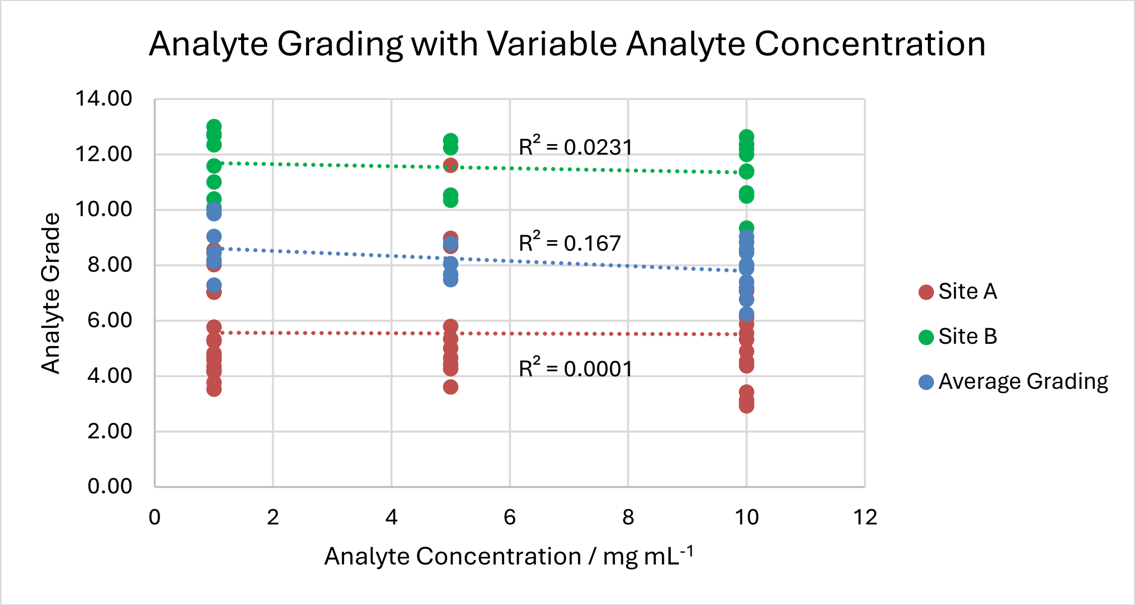

Figure S77 Scatterplot of analyte grade relationship to variable analyte concentration.

Table S83 Linear regression statistics for analyte grading with variable analyte concentration

	Standard Error	t-stat	P-value
Site A	2.195996	-0.06592	0.947797
Site B	1.046717	-0.68712	0.499898
Average	0.863419	-2.00242	0.058985

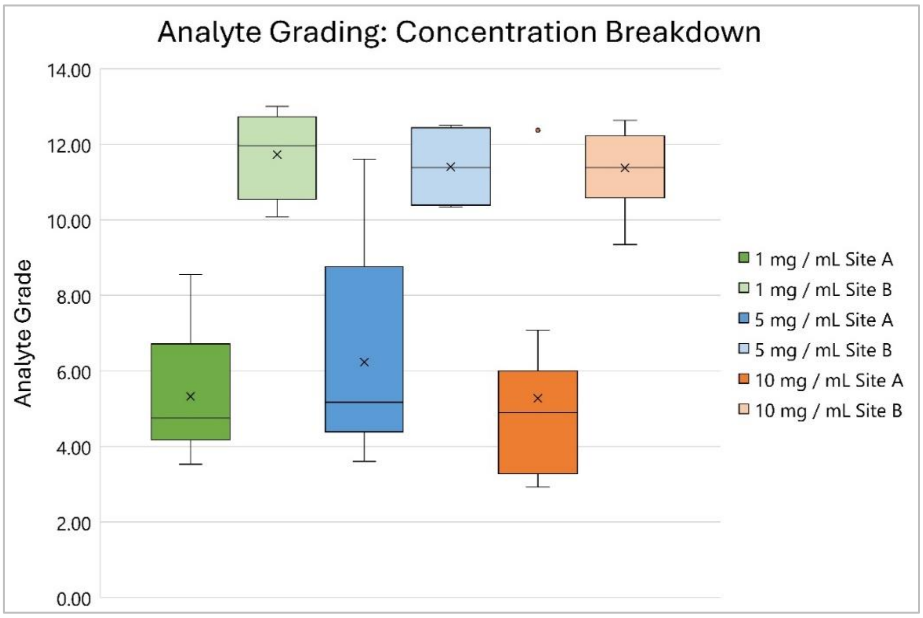

Figure S78 Box and whisker plot of analyte grade relationship to variable analyte concentration with components separated into crystallographic space groups.

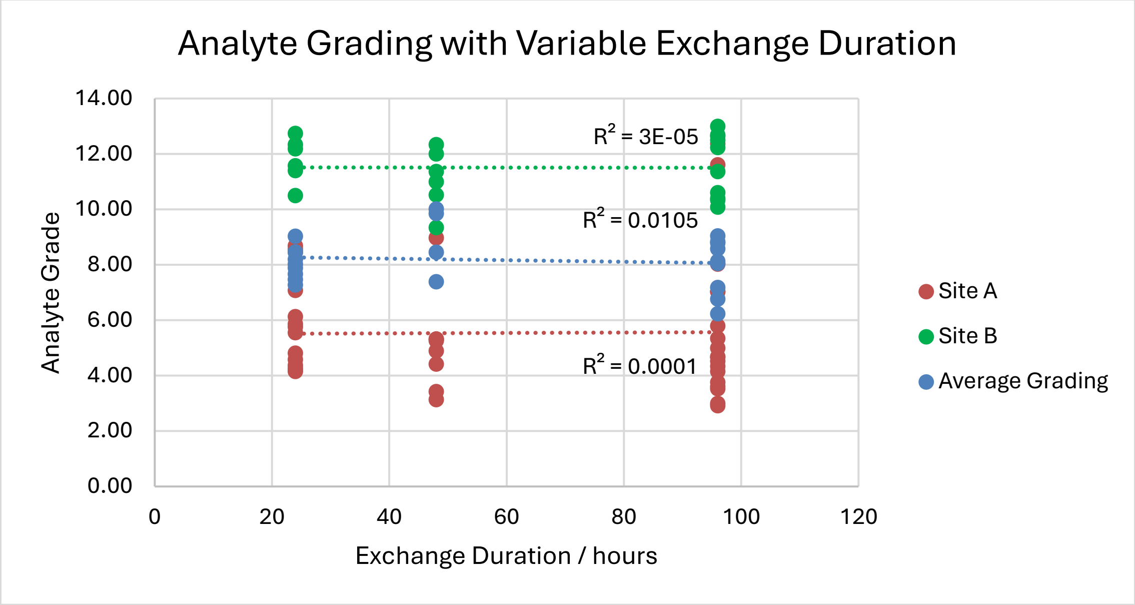

Figure S79 Scatterplot of analyte grade relationship to variable exchange duration

Table S84 Linear regression statistics for analyte grading with variable exchange duration

	Standard Error	t-stat	P-value
Site A	2.195991	0.067257	0.946740
Site B	1.058985	-0.023740	0.981295
Average	0.943135	0.350069	0.729946

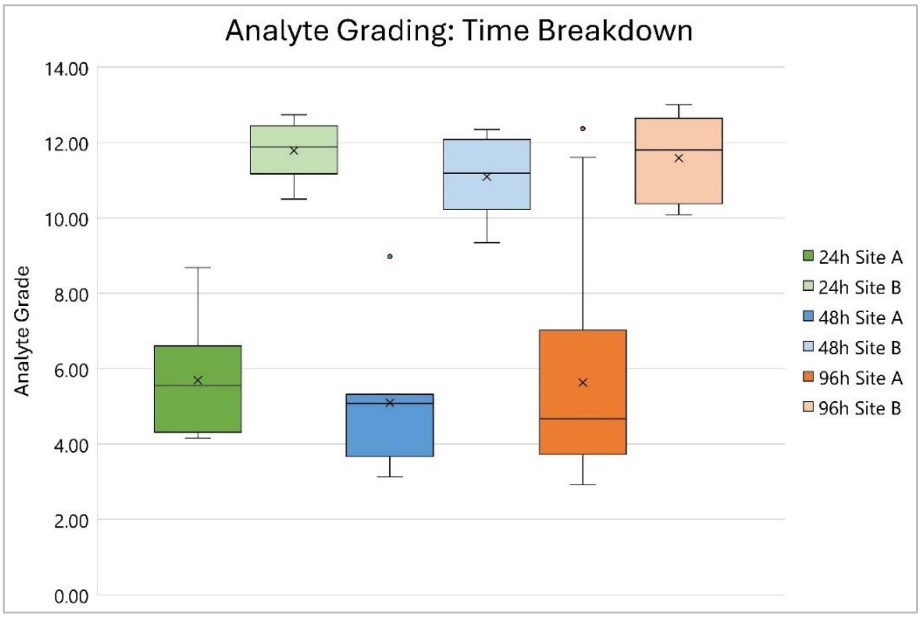

Figure S80 Box and whisker plot of analyte grade relationship to variable exchange duration with components separated into crystallographic space groups.

S12. Analyte Grading: Two Factor Influences

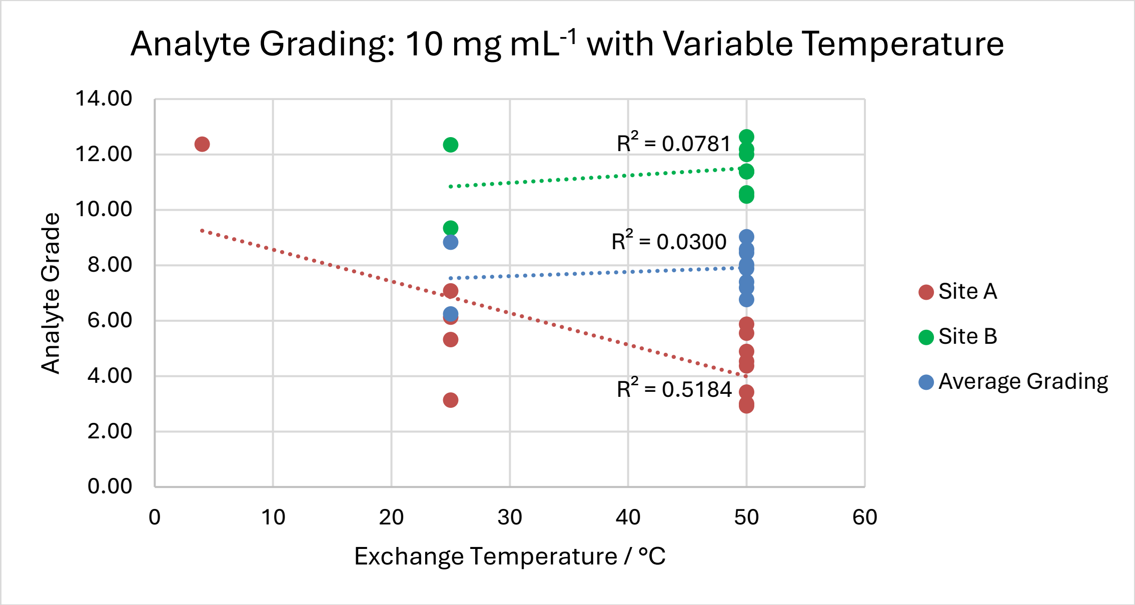

Figure S81 Scatterplot of analyte grade relationship to variable exchange temperature at 10 mg mL^{-1} analyte concentration.

Table S85 Linear regression statistics for analyte grading 10 mg mL^{-1} with variable exchange temperature

	Standard Error	t-stat	P-value
Site A	1.865053	-3.343530	0.007446
Site B	1.003216	0.837806	0.429814
Average	1.031561	0.465879	0.65545

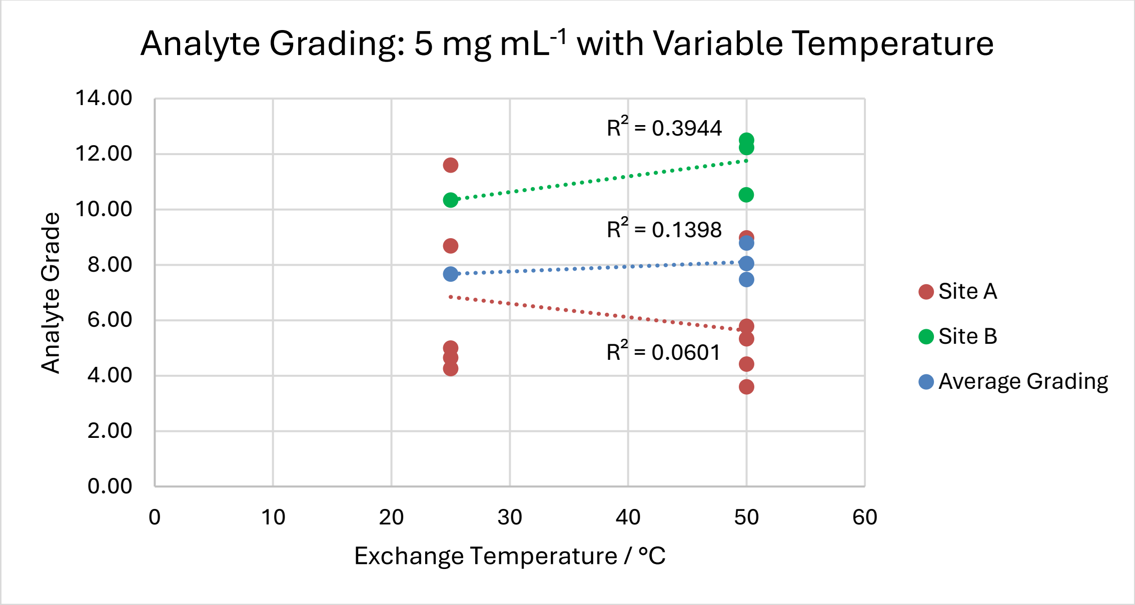

Figure S82 Scatterplot of analyte grade relationship to variable exchange temperature at 5 mg mL^{-1} analyte concentration.

Table S86 Linear regression statistics for analyte grading 5 mg mL^{-1} with variable exchange temperature

	Standard Error	t-stat	P-value
Site A	2.859992	-0.672150	0.523049
Site B	1.129007	1.083315	0.474554
Average	0.932536	0.403241	0.755985

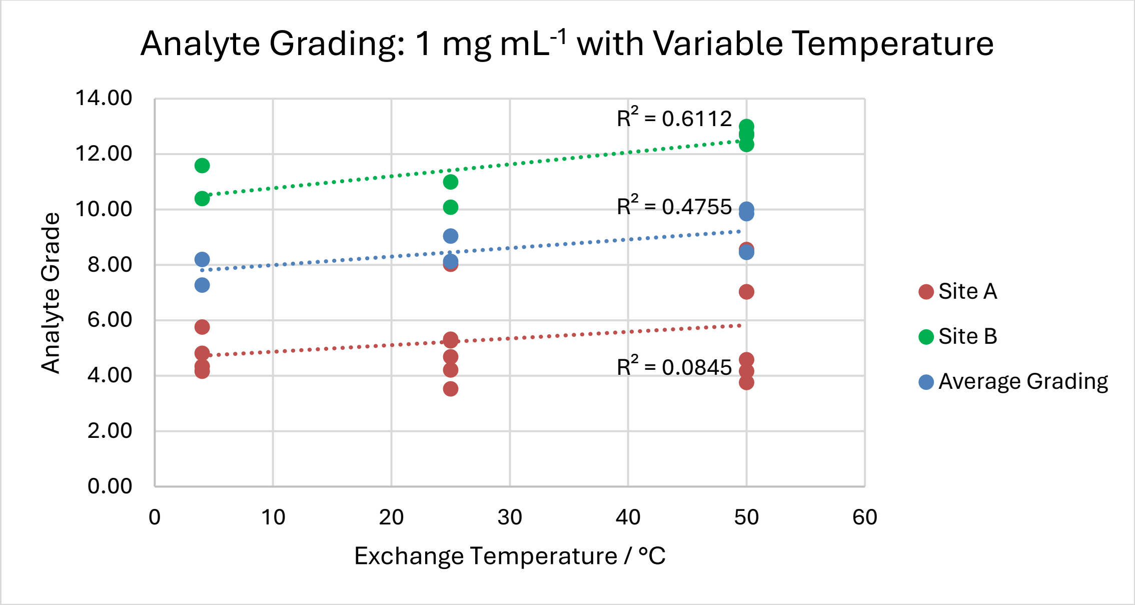

Figure S83 Scatterplot of analyte grade relationship to variable exchange temperature at 1 mg mL^{-1} analyte concentration.

Table S87 Linear regression statistics for analyte grading 1 mg mL^{-1} with variable exchange temperature

	Standard Error	t-stat	P-value
Site A	1.483870	1.167996	0.263783
Site B	0.805894	2.906461	0.033538
Average	0.613558	2.729176	0.041321

At lower concentration, an increase in temperature results in a poorer quality model for the secondary site. One possible explanation for this is the increase in kinetic energy improves diffusion and wider distribution of guests throughout the crystal, but because the analyte material is not in excess this leads to lower average occupancies overall, which negatively impacts data quality.

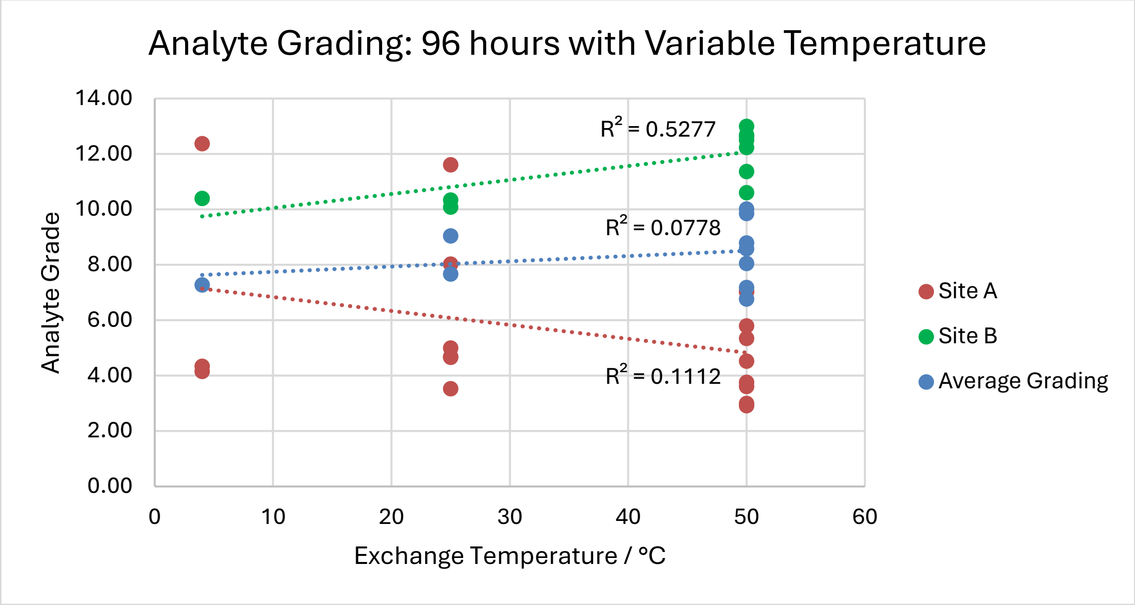

Figure S84 Scatterplot of analyte grade relationship to variable exchange temperature at 96 hours exchange duration.

Table S88 Linear regression statistics for analyte grading 96 hours with variable exchange temperature

	Standard Error	t-stat	P-value
Site A	2.677260	-1.389080	0.185087
Site B	0.891517	2.799623	0.026539
Average	1.219359	0.769765	0.466617

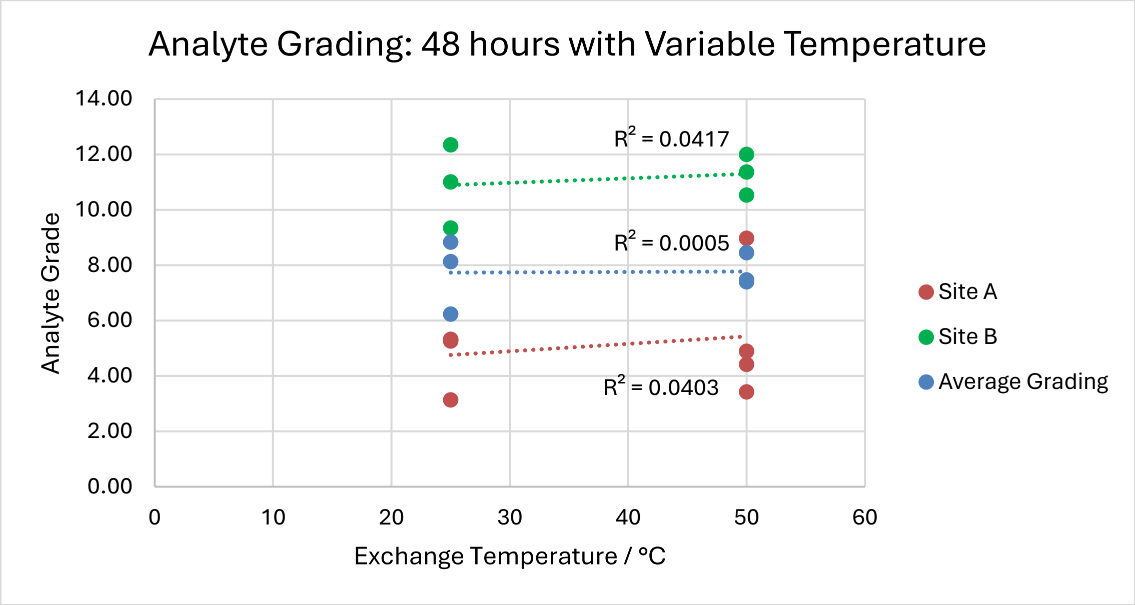

Figure S85 Scatterplot of analyte grade relationship to variable exchange temperature at 48 hours exchange duration.

Table S89 Linear regression statistics for analyte grading 48 hours with variable exchange temperature

	Standard Error	t-stat	P-value
Site A	2.014923	0.470992	0.657479
Site B	1.366564	0.361633	0.741596
Average	1.165116	0.041494	0.969509

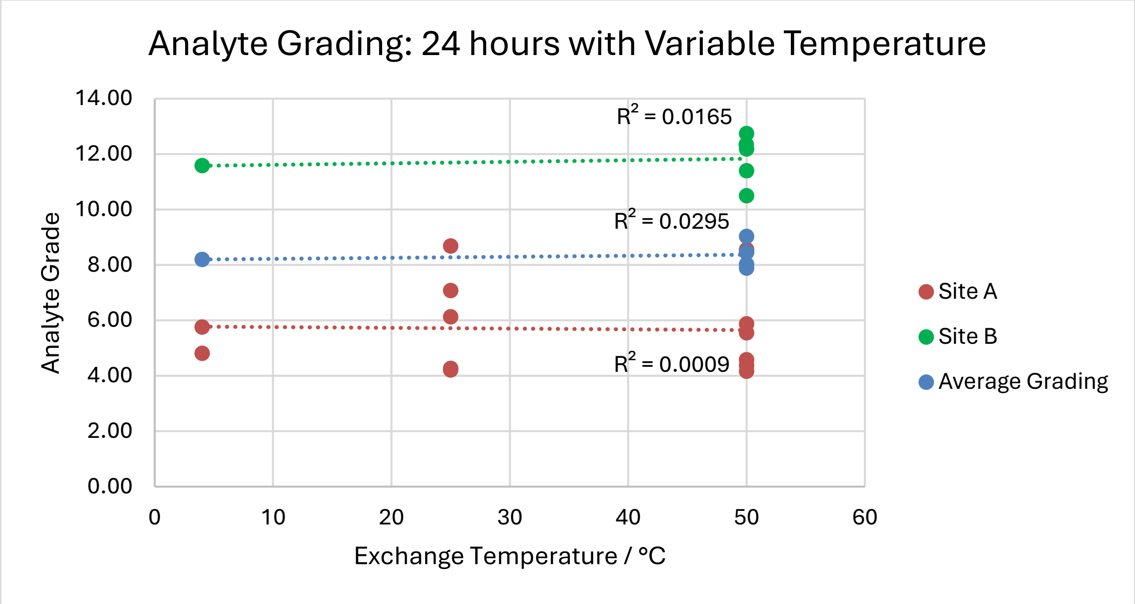

Figure S86 Scatterplot of analyte grade relationship to variable exchange temperature at 24 hours exchange duration.

Table S90 Linear regression statistics for analyte grading 24 hours with variable exchange temperature

	Standard Error	t-stat	P-value
Site A	1.623249	-0.102540	0.920355
Site B	1.025437	0.224899	0.836506
Average	0.370009	0.422013	0.701432

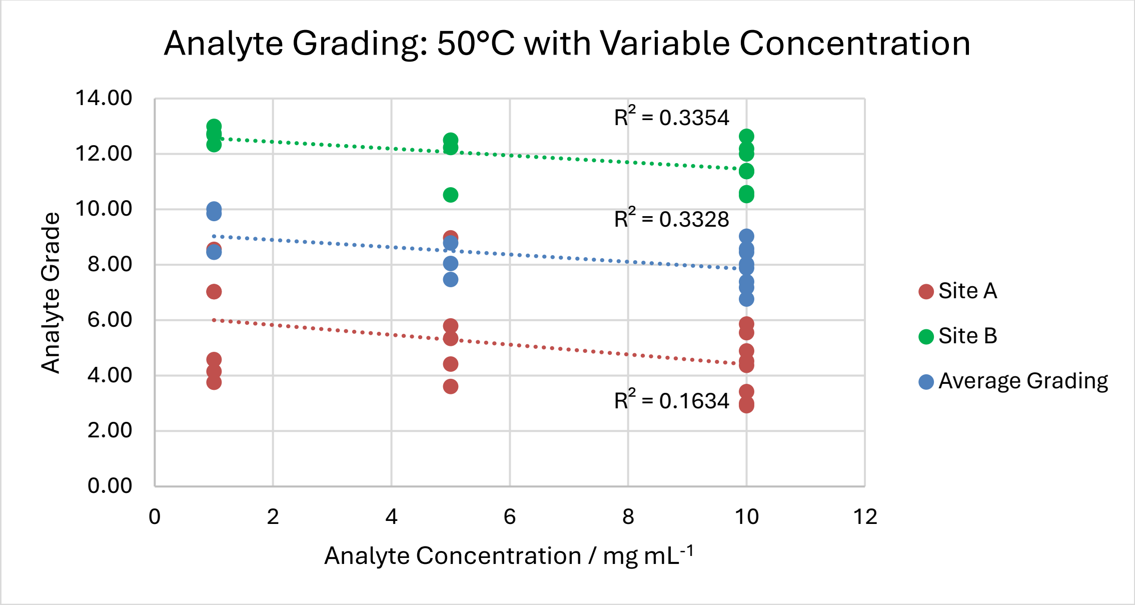

Figure S88 Scatterplot of analyte grade relationship to variable analyte concentration at 50°C exchange temperature.

Table S91 Linear regression statistics for analyte grading 50°C with variable analyte concentration

	Standard Error	t-stat	P-value
Site A	1.597613	-1.862210	0.081042
Site B	0.710877	-2.595750	0.023408
Average	0.729539	-2.703470	0.019186

At 50°C, increasing concentration has a positive correlation with quality of both primary and secondary guest sites. It is suggested that the combination of higher kinetic energy and a significant analyte concentration gradient around the host crystal have additive benefits for efficient diffusion of BBA-8,12-OMe molecules throughout the host framework. It should be noted that at 10 mg mL^{-1} there is a significant excess of guest as evidenced by the large amount of amorphous material which is observed in the bottom of the soaking vial.

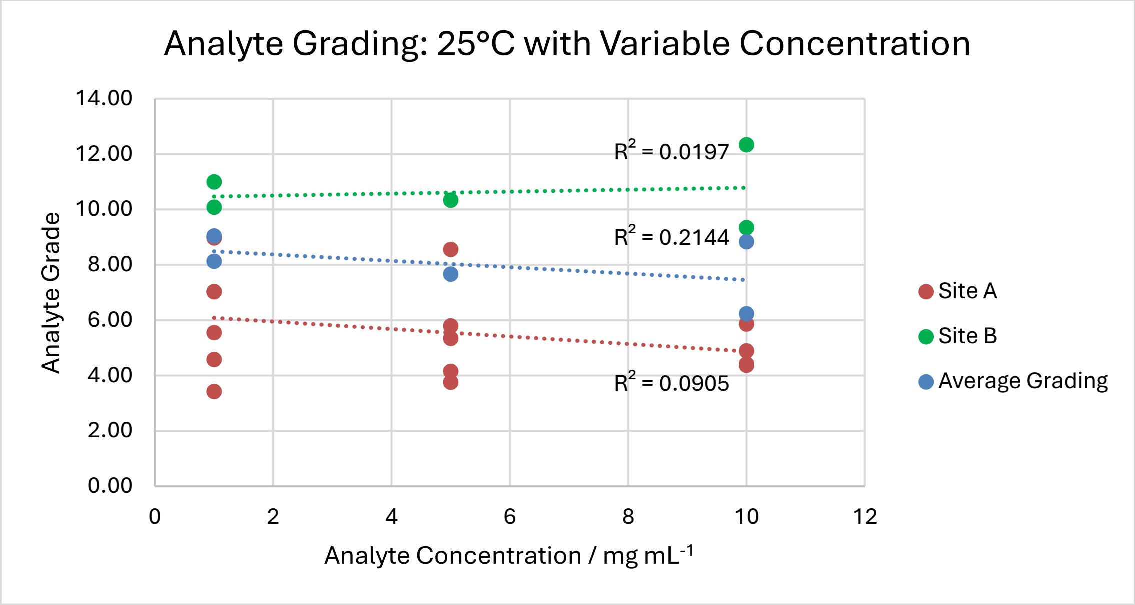

Figure S89 Scatterplot of analyte grade relationship to variable analyte concentration at 25°C exchange temperature.

Table S92 Linear regression statistics for analyte grading 25°C with variable analyte concentration

	Standard Error	t-stat	P-value
Site A	2.370451	0.222268	0.827844
Site B	0.413482	0.768441	0.522560
Average	0.725648	-1.428030	0.289465

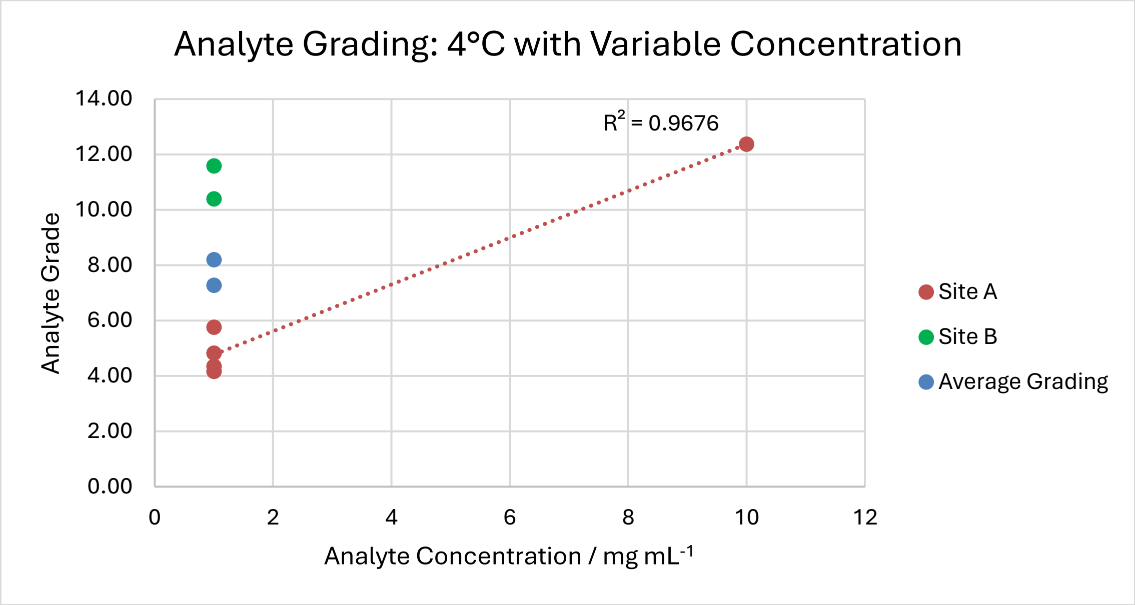

Figure S90 Scatterplot of analyte grade relationship to variable analyte concentration at 4°C exchange temperature.

Table S93 Linear regression statistics for analyte grading 4°C with variable analyte concentration

	Standard Error	t-stat	P-value
Site A	0.726861	9.350444	0.011245
Site B	-	-	-
Average	-	-	-

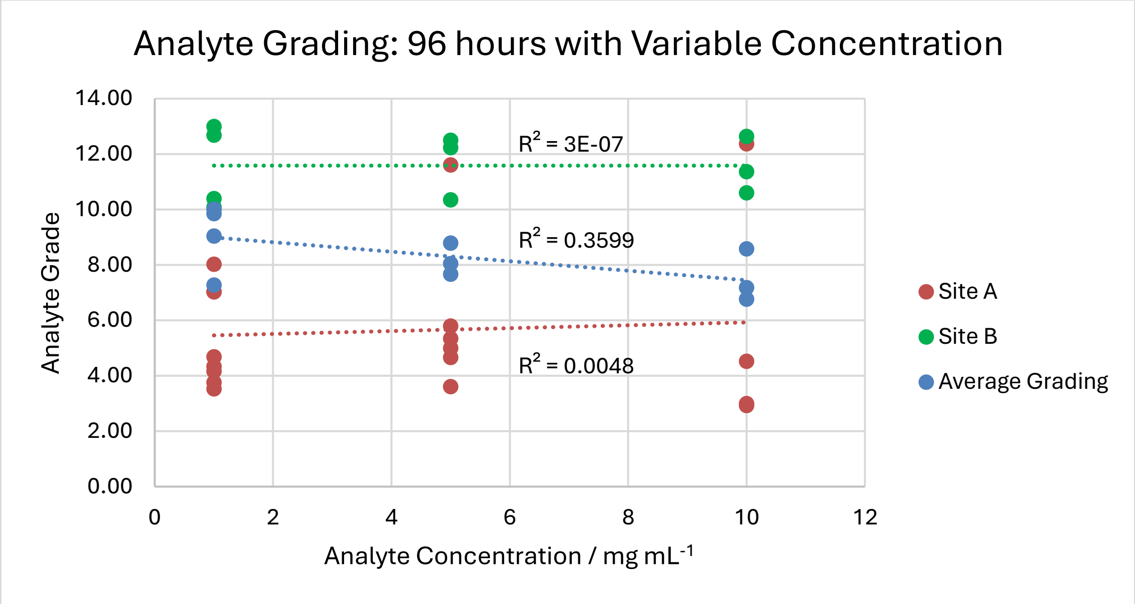

Figure S91 Scatterplot of analyte grade relationship to variable analyte concentration at 96 hours exchange duration.

Table S94 Linear regression statistics for analyte grading 96 hours with variable analyte concentration

	Standard Error	t-stat	P-value
Site A	2.816816	0.273991	0.787829
Site B	1.291204	-0.001380	0.998935
Average	1.012312	-1.994860	0.086271

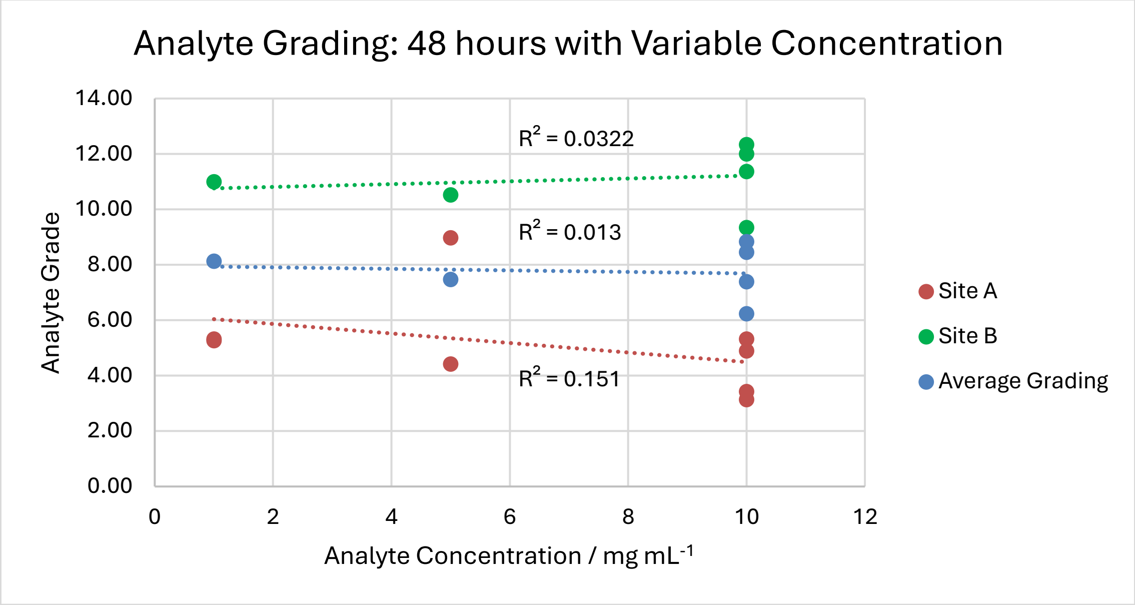

Figure S92 Scatterplot of analyte grade relationship to variable analyte concentration at 48 hours exchange duration.

Table S95 Linear regression statistics for analyte grading 48 hours with variable analyte concentration

	Standard Error	t-stat	P-value
Site A	1.876224	-0.978670	0.37269
Site B	1.374057	0.316086	0.772652
Average	1.162718	-0.203070	0.852075

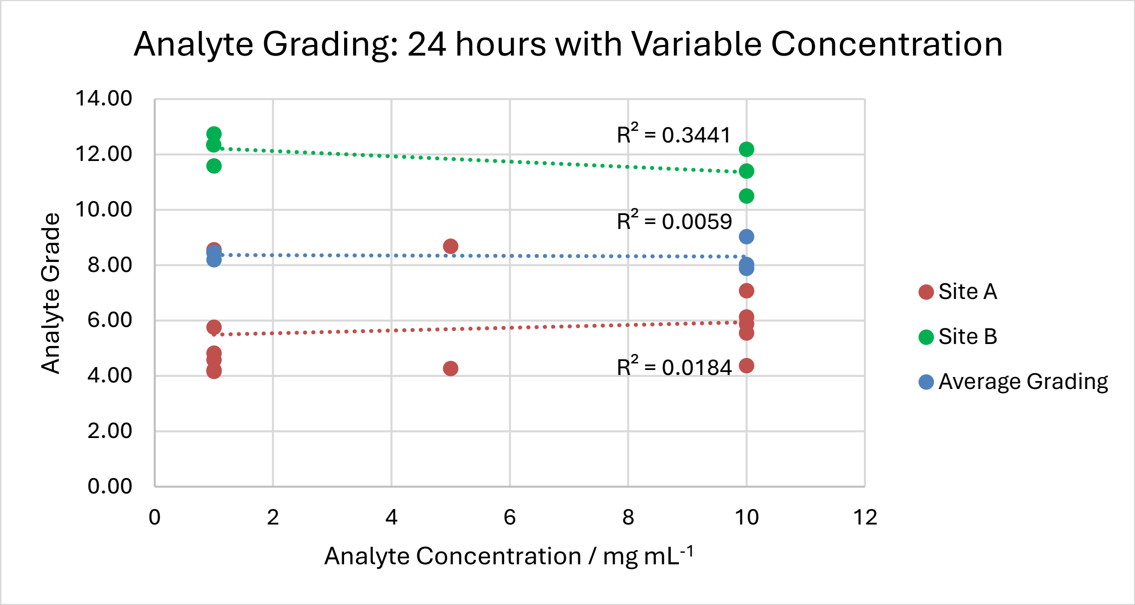

Figure S93 Scatterplot of analyte grade relationship to variable analyte concentration at 24 hours exchange duration.

Table S96 Linear regression statistics for analyte grading 24 hours with variable analyte concentration

	Standard Error	t-stat	P-value
Site A	1.618050	0.456962	0.657464
Site B	0.616842	-1.706840	0.186391
Average	0.325398	-0.214600	0.843835

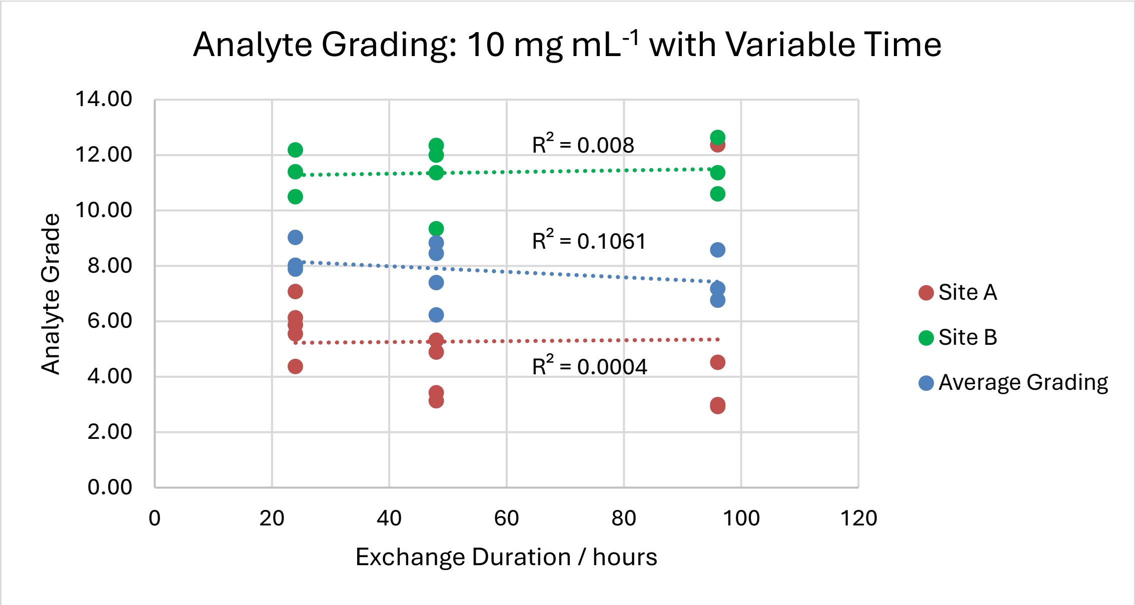

Figure S94 Scatterplot of analyte grade relationship to variable exchange duration at 10 mg mL^{-1} analyte concentration.

Table S97 Linear regression statistics for analyte grading 10 mg mL^{-1} with variable exchange duration

	Standard Error	t-stat	P-value
Site A	2.735822	0.065349	0.949184
Site B	1.070752	0.251333	0.808775
Average	0.987330	-0.914950	0.390646

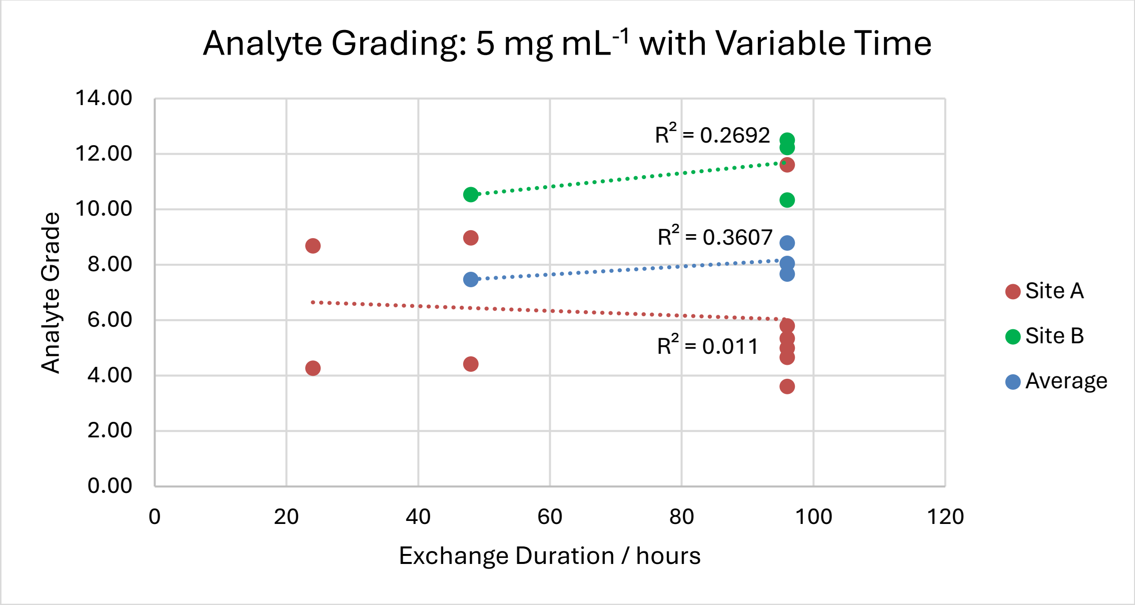

Figure S95 Scatterplot of analyte grade relationship to variable exchange duration at 5 mg mL^{-1} analyte concentration.

Table S98 Linear regression statistics for analyte grading 5 mg mL^{-1} with variable exchange duration

	Standard Error	t-stat	P-value
Site A	2.849677	-0.288580	0.781259
Site B	1.549944	0.651871	0.632232
Average	0.726358	0.831462	0.558420

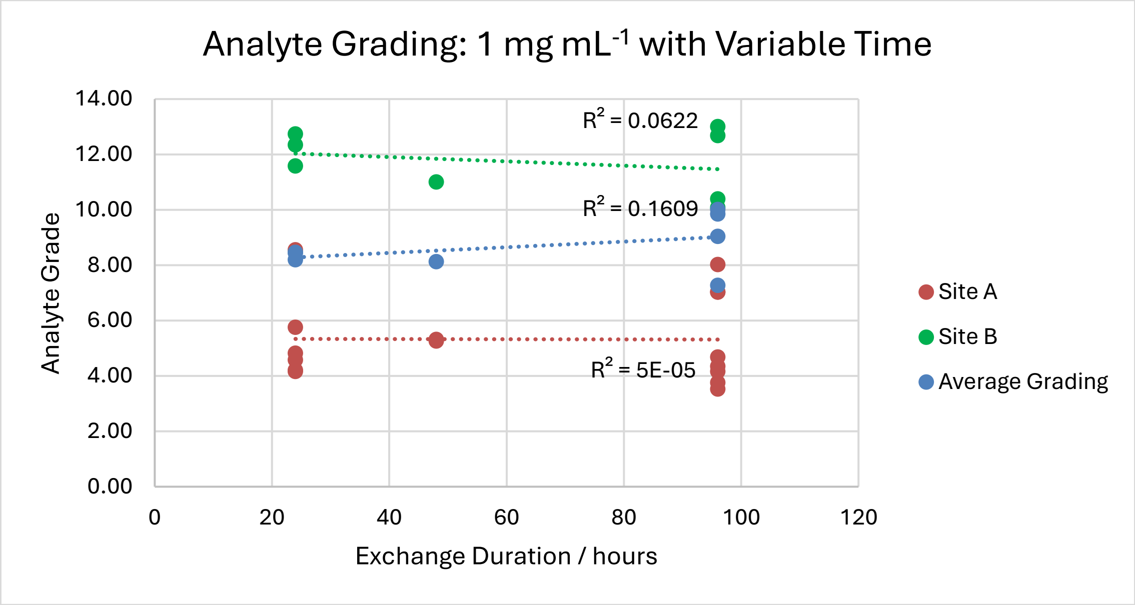

Figure S96 Scatterplot of analyte grade relationship to variable exchange duration at 1 mg mL^{-1} analyte concentration.

Table S99 Linear regression statistics for analyte grading 1 mg mL^{-1} with variable exchange duration

	Standard Error	t-stat	P-value
Site A	1.587913	-0.025740	0.979859
Site B	1.062194	-0.703420	0.513190
Average	0.890122	1.094311	0.323712

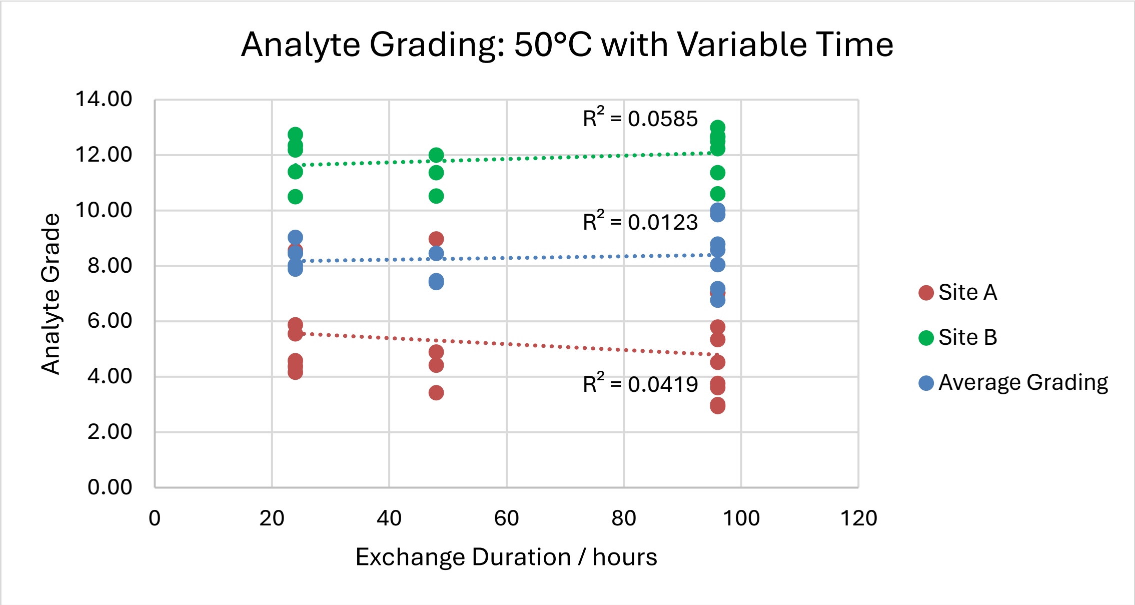

Figure S97 Scatterplot of analyte grade relationship to variable exchange duration at 50°C exchange temperature.

Table S100 Linear regression statistics for analyte grading 50°C with variable exchange duration

	Standard Error	t-stat	P-value
Site A	1.743155	-0.864450	0.400116
Site B	0.890314	0.865316	0.403833
Average	0.956621	0.396909	0.698403

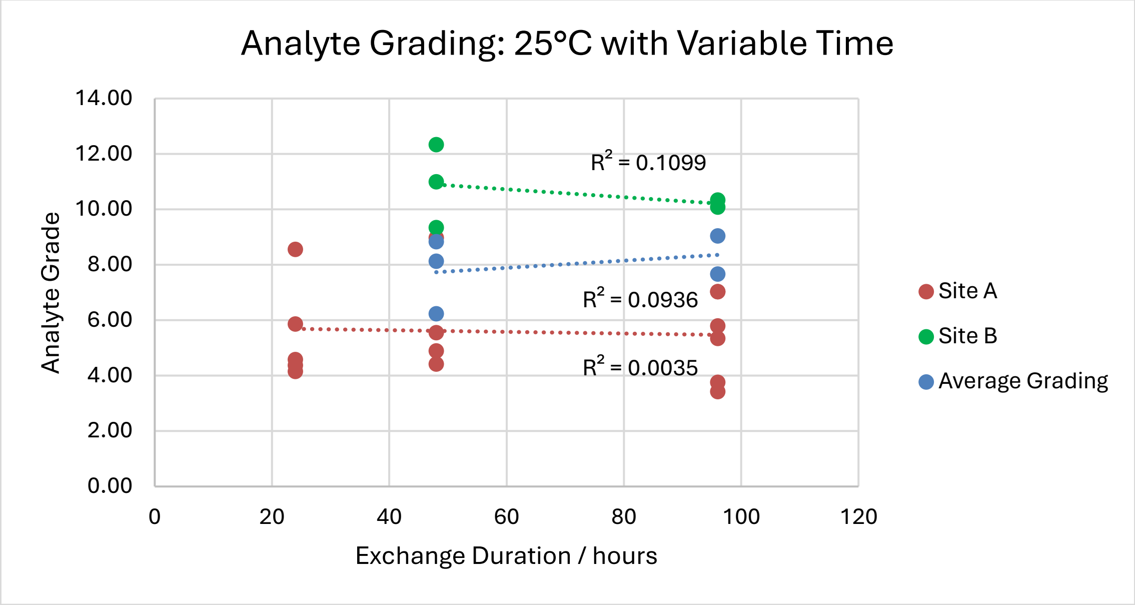

Figure S98 Scatterplot of analyte grade relationship to variable exchange duration at 50°C exchange temperature.

Table S101 Linear regression statistics for analyte grading 25°C with variable exchange duration

	Standard Error	t-stat	P-value
Site A	2.353325	0.341826	0.738394
Site B	0.725788	-1.032690	0.410271
Average	0.789450	0.867253	0.477229

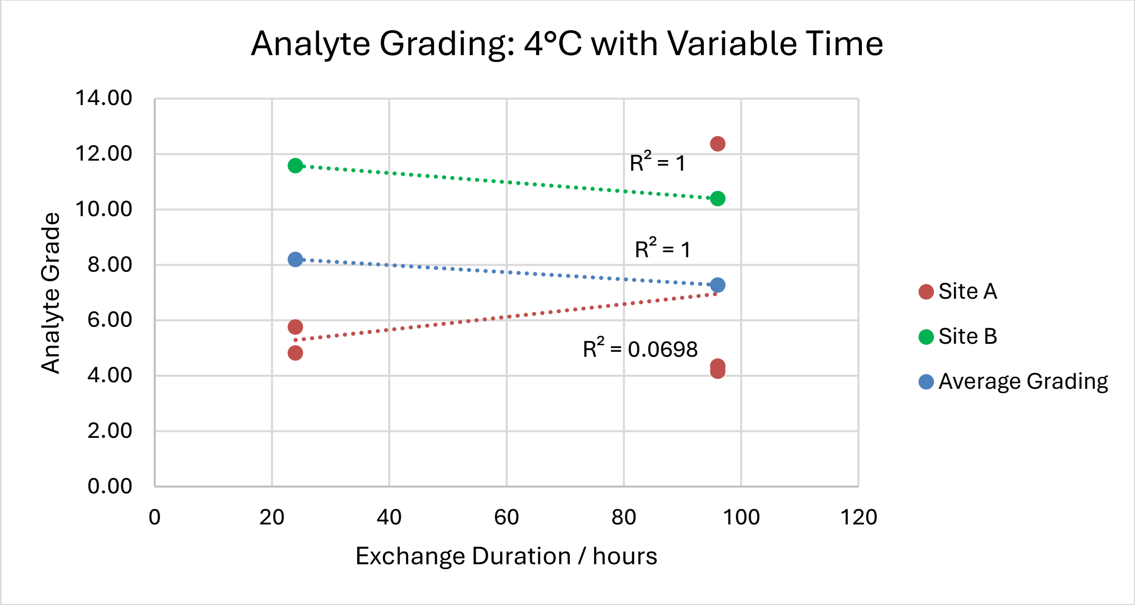

Figure S99 Scatterplot of analyte grade relationship to variable exchange duration at 4°C exchange temperature.

Table S102 Linear regression statistics for analyte grading 4°C with variable exchange duration

	Standard Error	t-stat	P-value
Site A	4.207144	0.433962	0.706643
Site B	-	-	-
Average	-	-	-

S13. Analyte Grading: Traditional Crystallographic Metrics

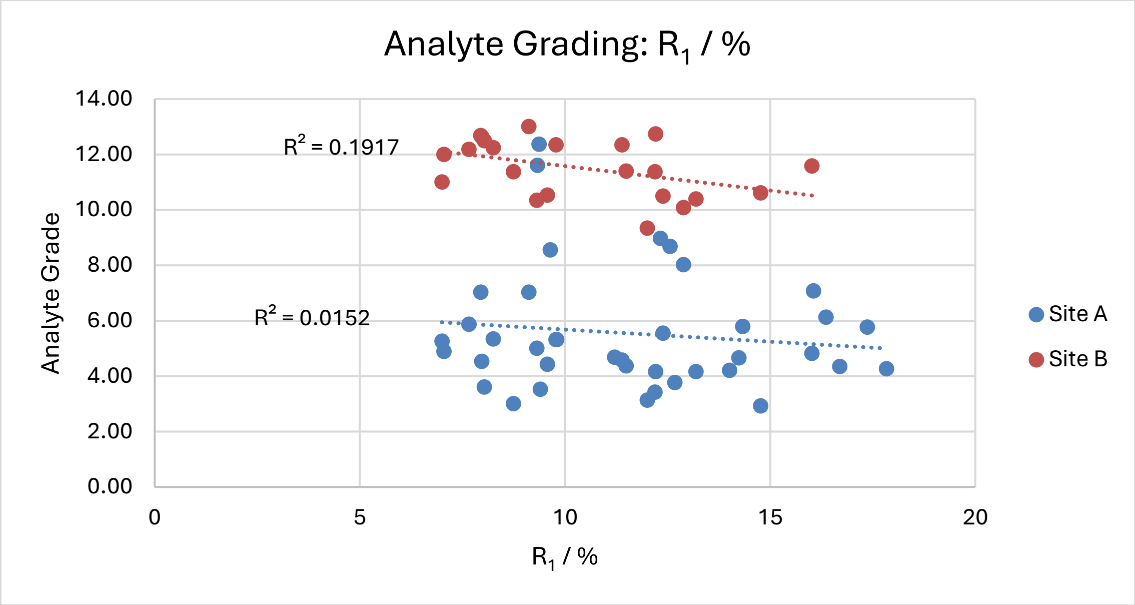

Figure S100 Scatterplot of analyte grade relationship to R_1 / %.

Table S103 Linear regression statistics for analyte grade relationship to R_1 / %

	Standard Error	t-stat	P-value
Site A	2.179360	-0.755940	0.454468
Site B	0.952129	-2.177560	0.041584

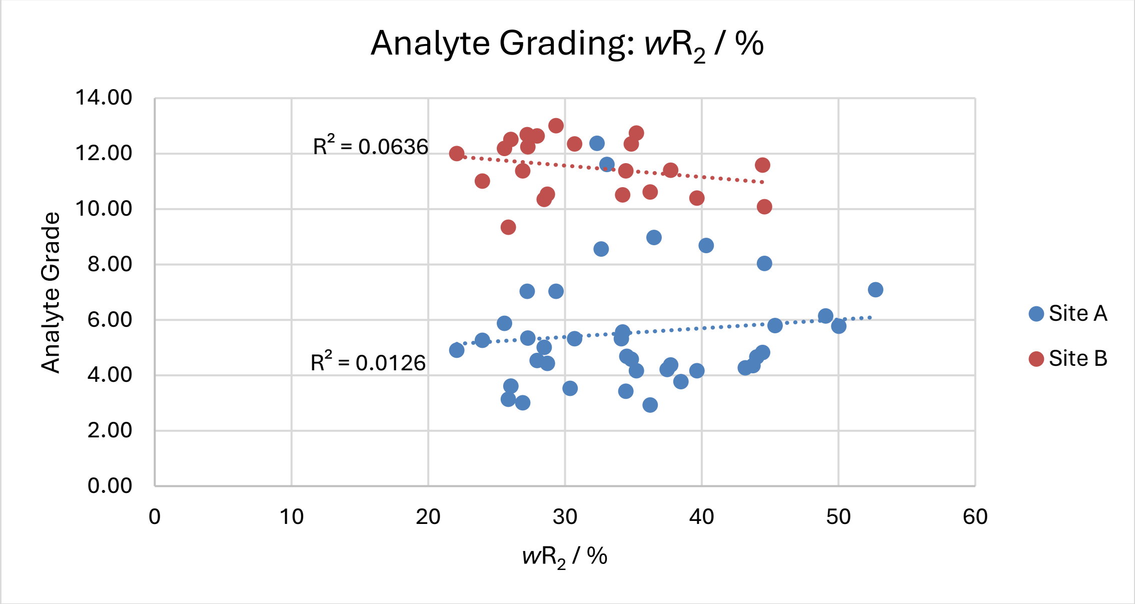

Figure S101 Scatterplot of analyte grade relationship to wR_2 / %.

Table S104 Linear regression statistics for analyte grade relationship to wR_2 / %

	Standard Error	t-stat	P-value
Site A	2.182232	0.687484	0.496065
Site B	1.024794	-1.165080	0.257697

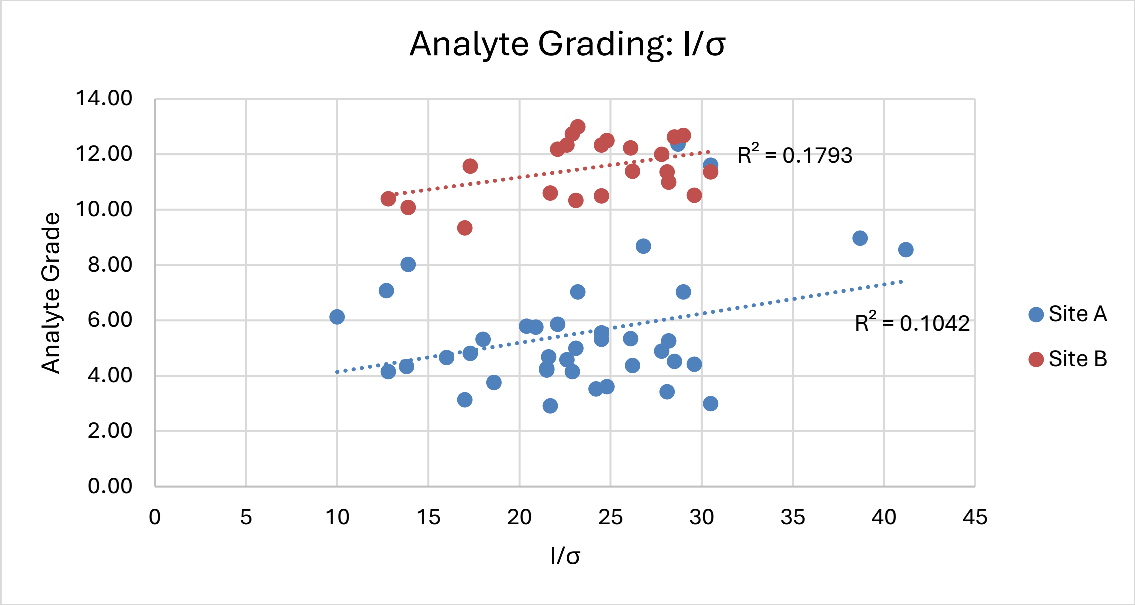

Figure S102 Scatterplot of analyte grade relationship to intensity-to-signal (I/σ) ratio.

Table S105 Linear regression statistics for analyte grade relationship to intensity-to-signal (I/σ) ratio

	Standard Error	t-stat	P-value
Site A	2.078575	2.074437	0.045050
Site B	0.959367	2.090414	0.049558

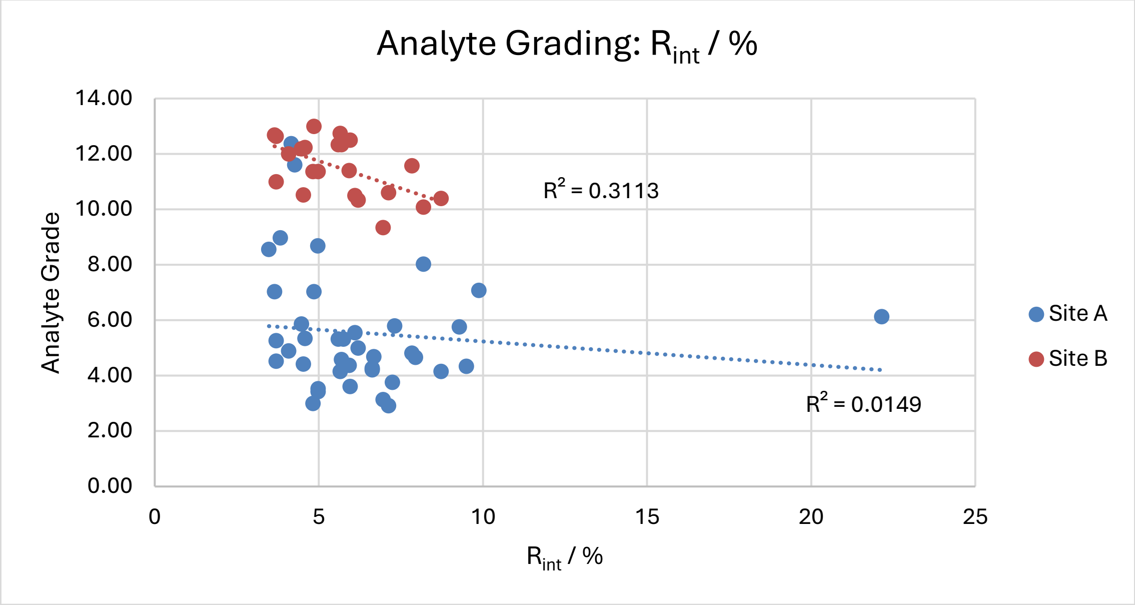

Figure S103 Scatterplot of analyte grade relationship to R_{int} / %.

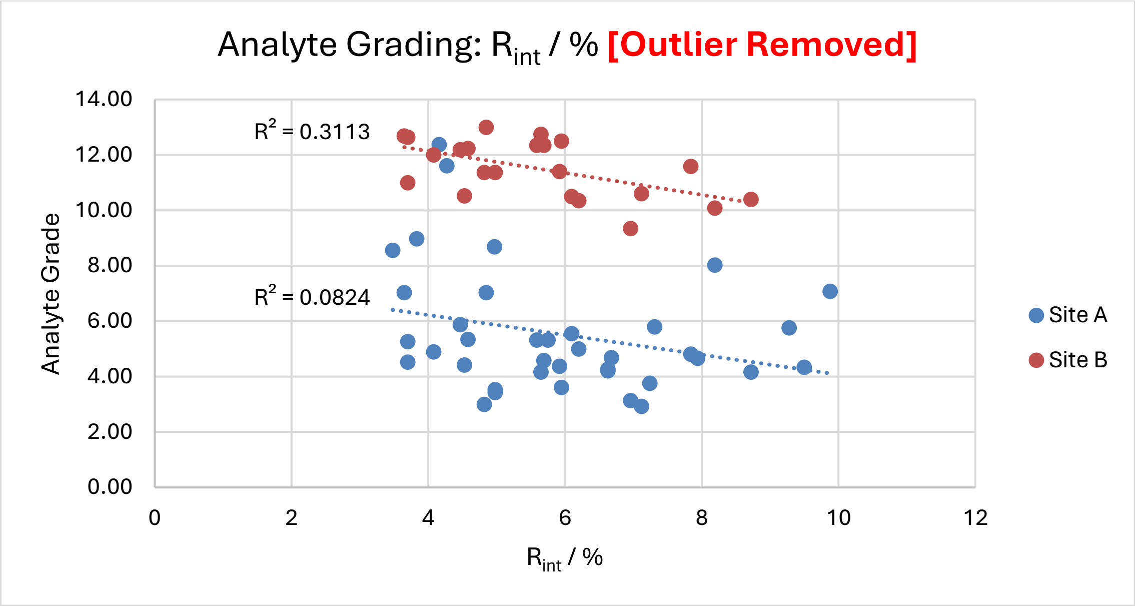

Figure S104 Scatterplot of analyte grade relationship to R_{int} / % (outlier data removed).

Table S106 Linear regression statistics for analyte grade relationship to R_{int} / %

	Standard Error	t-stat	P-value
Site A	2.179720	-0.747680	0.459379
Site A (Outlier Removed)	2.130620	-1.797630	0.080628
Site B	0.878840	-3.006720	0.006970

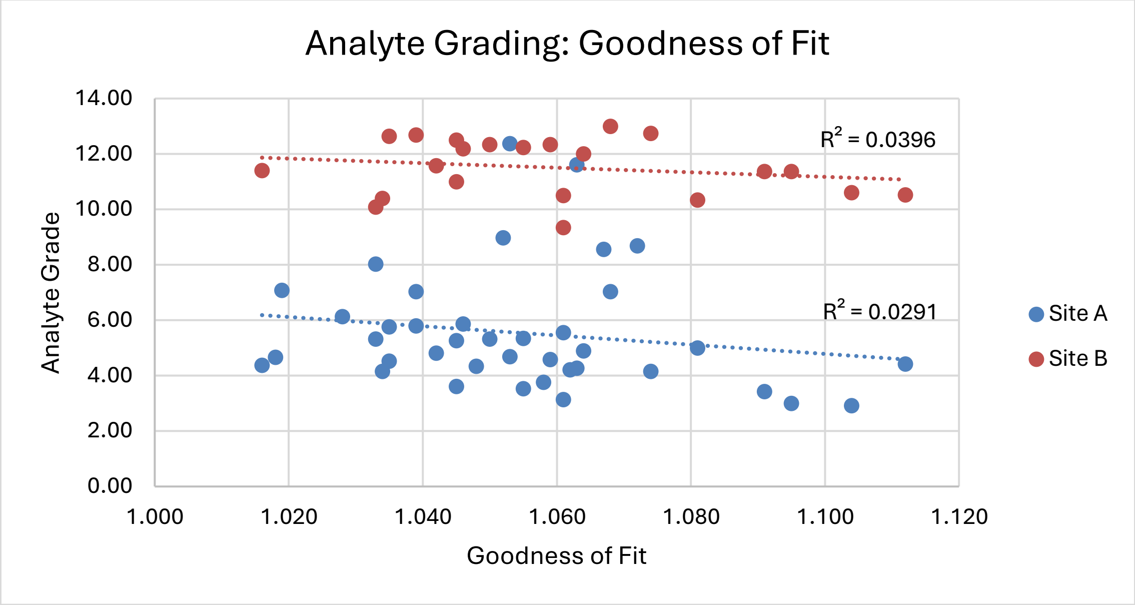

Figure S105 Scatterplot of analyte grade relationship to goodness of fit (GooF).

Table S107 Linear regression statistics for analyte grade relationship to goodness of fit (GooF)

	Standard Error	t-stat	P-value
Site A	2.163956	-1.052750	0.299285
Site B	1.037819	-0.908140	0.374620

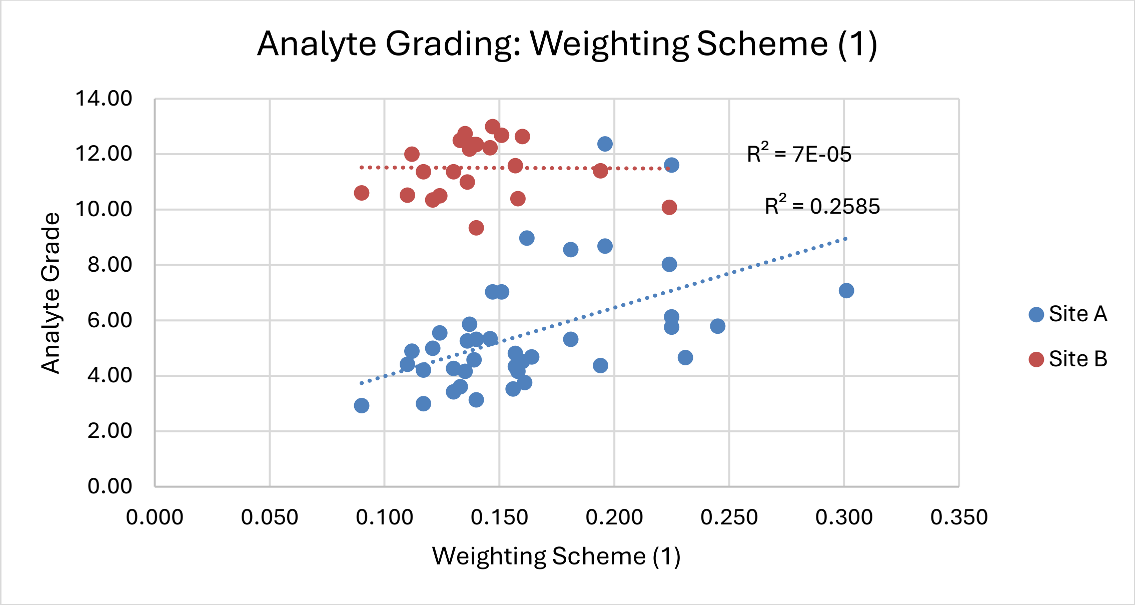

Figure S106 Scatterplot of analyte grade relationship to weighting scheme (WGHT) first value.

Table S108 Linear regression statistics for analyte grade relationship to weighting scheme first value

	Standard Error	t-stat	P-value
Site A	1.891052	3.591795	0.000950
Site B	1.058965	-0.03629	0.971410

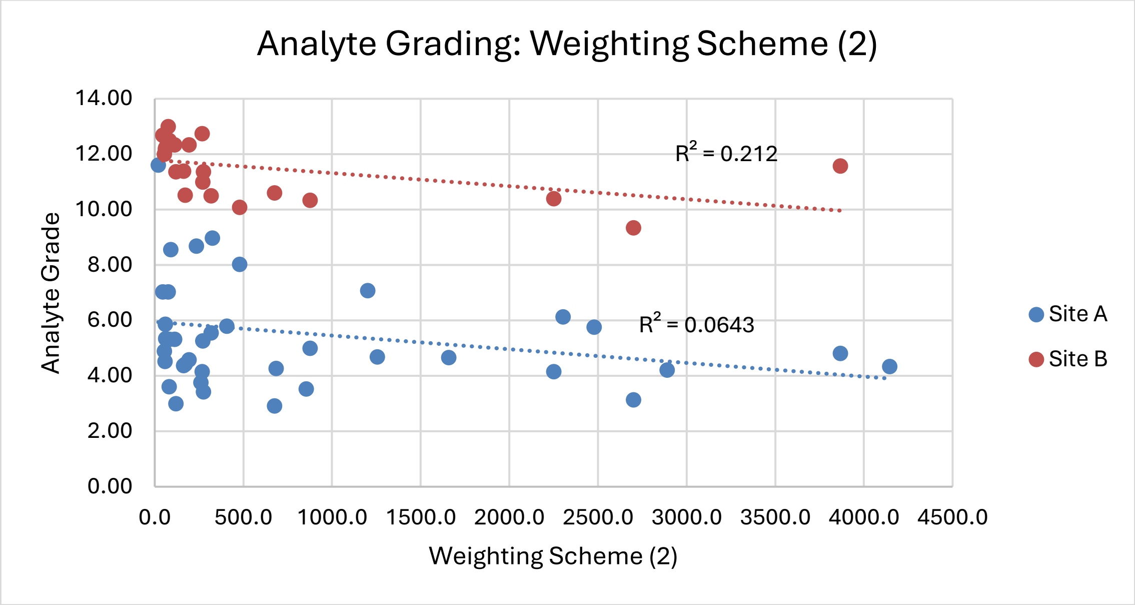

Figure S107 Scatterplot of analyte grade relationship to weighting scheme (WGHT) second value.

Table S109 Linear regression statistics for analyte grade relationship to weighting scheme second value

	Standard Error	t-stat	P-value
Site A	2.124333	-1.594710	0.119286
Site B	0.940062	-2.319700	0.031051

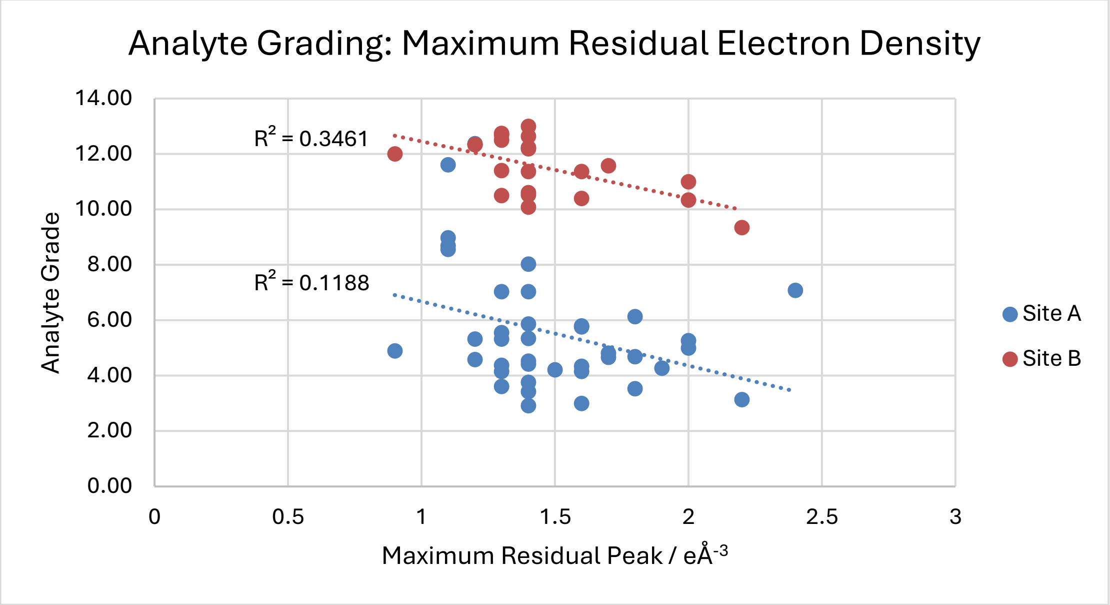

Figure S108 Scatterplot of analyte grade relationship to maximum residual electron density peak

Table S110 Linear regression statistics for analyte grade relationship to maximum residual electron density

	Standard Error	t-stat	P-value
Site A	2.061497	-2.233930	0.031615
Site B	0.856337	-3.253720	0.003977

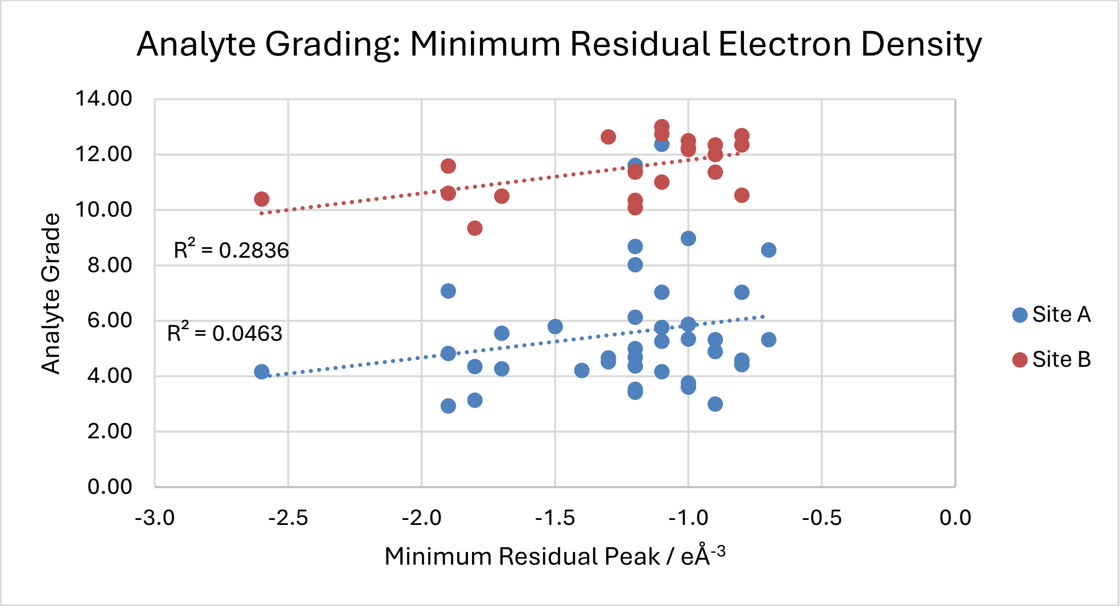

Figure S109 Scatterplot of analyte grade relationship to minimum residual electron density peak

Table S111 Linear regression statistics for analyte grade relationship to minimum residual electron density

	Standard Error	t-stat	P-value
Site A	2.144638	1.340849	0.188144
Site B	0.896334	2.813872	0.010723

S14. 4-Methylbenzophenone Crystallographic Tables, Diagrams, and Notes

Table S112 4-Methylbenzophenone 4_10_96a Crystallographic Table

CCDC Deposition Number	2343727
Empirical formula	$C_{36}H_{24}I_6N_{12}Zn_3$
Formula weight	1582.18
Temperature/K	100.0(3)
Crystal system	monoclinic
Space group	C2/c
a/Å	35.2833(5)
b/Å	14.9084(2)
c/Å	31.7352(4)
α = γ /°	90
β/°	102.7210(10)
Volume/Å^3	16283.5(4)
Z	8
ρ_{calc}g/cm^3	1.291
μ/mm^{-1}	19.082
F(000)	5856
Crystal size/mm^3	0.37 × 0.1 × 0.07
Radiation	Cu Kα (λ = 1.54184)
2Θ range for data collection/°	5.136 to 136.5
Index ranges	-42 ≤ h ≤ 41, -14 ≤ k ≤ 17, -38 ≤ l ≤ 38
Reflections collected	71698
Independent reflections	14786 [R_{int} = 0.0259, R_{sigma} = 0.0192]
Data/restraints/parameters	14786/27/606
Goodness-of-fit on F^2	1.098
Final R indexes [I>=2σ (I)]	R_1 = 0.0323, wR_2 = 0.0931
Largest diff. peak/hole / e Å^{-3}	0.75/-0.45
No guests or solvent were identified from the electron density map, SQUEEZE analysis indicated one void with a volume of 1018Å^3 and electron count of 299. This is equivalent to 6.23 cyclohexane molecules or 2.88 4-methylbenzophenone molecules.	

Table S113 4-Methylbenzophenone 4_10_96b Crystallographic Table

CCDC Deposition Number	2343728
Empirical formula	$C_{36}H_{24}I_6N_{12}Zn_3$
Formula weight	1582.18
Temperature/K	100.01(10)
Crystal system	monoclinic
Space group	C2/c
a/Å	35.3636(7)
b/Å	14.9223(2)
c/Å	31.7483(6)
α = γ /°	90
β/°	102.783(2)
Volume/Å^3	16338.5(5)
Z	8
ρ_{calc}g/cm^3	1.286
μ/mm^{-1}	19.018
F(000)	5856
Crystal size/mm^3	0.21 × 0.15 × 0.11
Radiation	Cu Kα (λ = 1.54184)
2Θ range for data collection/°	5.124 to 136.502
Index ranges	-42 ≤ h ≤ 36, -17 ≤ k ≤ 15, -38 ≤ l ≤ 36
Reflections collected	65436
Independent reflections	14822 [R_{int} = 0.0355, R_{sigma} = 0.0254]
Data/restraints/parameters	14822/21/606
Goodness-of-fit on F^2	1.083
Final R indexes [I>=2σ (I)]	R_1 = 0.0474, wR_2 = 0.1466
Largest diff. peak/hole / e Å^{-3}	1.27/-0.61
No guests or solvent were identified from the electron density map, SQUEEZE analysis indicated one void with a volume of 1027Å^3 and electron count of 261. This is equivalent to 5.44 cyclohexane molecules or 2.51 4-methylbenzophenone molecules.	

Table S114 4-Methylbenzophenone 4_10_96c Crystallographic Table

CCDC Deposition Number	2343729
Empirical formula	$C_{36}H_{24}I_6N_{12}Zn_3$
Formula weight	1582.18
Temperature/K	100.00(10)
Crystal system	monoclinic
Space group	C2/c
a/Å	35.3704(5)
b/Å	14.9099(2)
c/Å	31.7401(4)
α = γ /°	90
β/°	102.7800(10)
Volume/Å^3	16324.1(4)
Z	8
ρ_{calc}g/cm^3	1.288
μ/mm^{-1}	19.034
F(000)	5856
Crystal size/mm^3	0.29 × 0.17 × 0.08
Radiation	Cu Kα (λ = 1.54184)
2Θ range for data collection/°	5.124 to 136.5
Index ranges	-42 ≤ h ≤ 41, -17 ≤ k ≤ 14, -38 ≤ l ≤ 36
Reflections collected	64990
Independent reflections	14777 [R_{int} = 0.0325, R_{sigma} = 0.0242]
Data/restraints/parameters	14777/25/614
Goodness-of-fit on F^2	1.118
Final R indexes [I>=2σ (I)]	R_1 = 0.0443, wR_2 = 0.1357
Largest diff. peak/hole / e Å^{-3}	1.06/-0.67
No guests or solvent were identified from the electron density map, SQUEEZE analysis indicated one void with a volume of 1005Å^3 and electron count of 301. This is equivalent to 6.27 cyclohexane molecules or 2.89 4-methylbenzophenone molecules.	

Table S115 4-Methylbenzophenone 50_10_96a Crystallographic Table

CCDC Deposition Number	2343730
Empirical formula	$C_{252.44}H_{188.95}I_{24}N_{48}O_{7.75}Zn_{12}$
Formula weight	7848.75
Temperature/K	100.00(10)
Crystal system	triclinic
Space group	P-1
a/Å	18.8343(3)
b/Å	29.6438(5)
c/Å	32.8648(5)
α /°	67.9240(10)
β/°	74.7400(10)
γ /°	73.0100(10)
Volume/Å^3	16016.3(5)
Z	2
ρ_{calc}g/cm^3	1.627
μ/mm^{-1}	19.552
F(000)	7467
Crystal size/mm^3	0.28 × 0.21 × 0.1
Radiation	Cu Kα (λ = 1.54184)
2Θ range for data collection/°	4.982 to 136.502
Index ranges	-22 ≤ h ≤ 21, -35 ≤ k ≤ 35, -39 ≤ l ≤ 39
Reflections collected	254275
Independent reflections	58479 [R_{int} = 0.0699, R_{sigma} = 0.0431]
Data/restraints/parameters	58479/1626/3592
Goodness-of-fit on F^2	1.045
Final R indexes [I>=2σ (I)]	R_1 = 0.1089, wR_2 = 0.3369
Largest diff. peak/hole / e Å^{-3}	2.70/-2.47
SQUEEZE analysis indicated one void with a volume of 773Å^3 and electron count of 171. This is equivalent to 3.56 cyclohexane molecules or 1.64 4-methylbenzophenone molecules.	

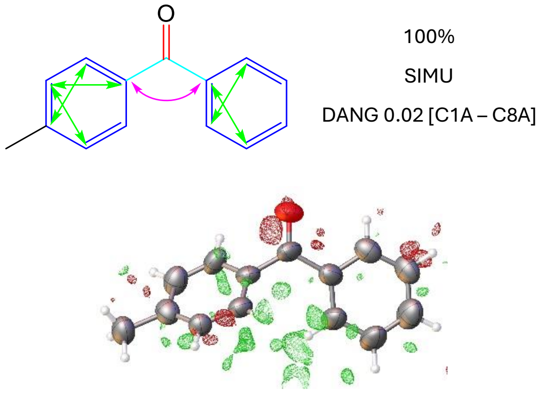

Figure S110 Geometric restraints and thermal ellipsoid (50% probability) for 50_10_96a Site A

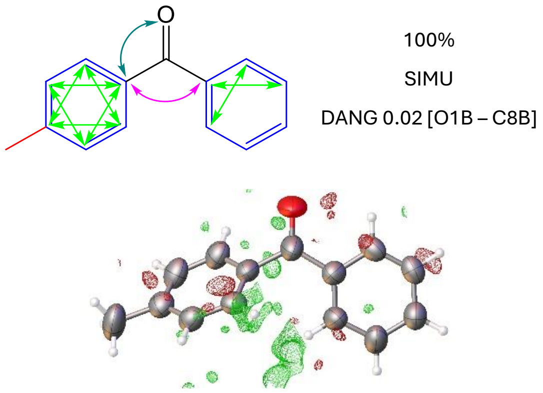

Figure S111 Geometric restraints and thermal ellipsoid (50% probability) for 50_10_96a Site B

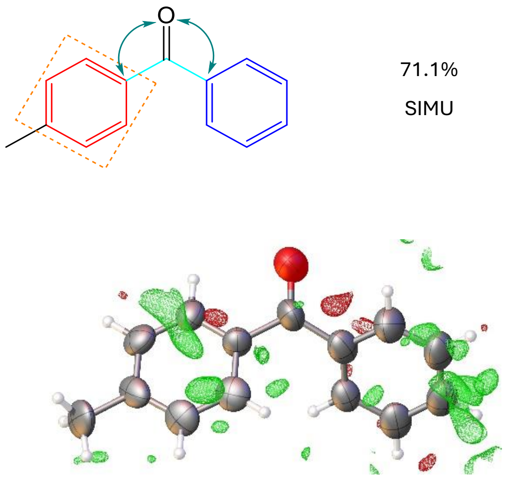

Figure S112 Geometric restraints and thermal ellipsoid (50% probability) for 50_10_96a Site C

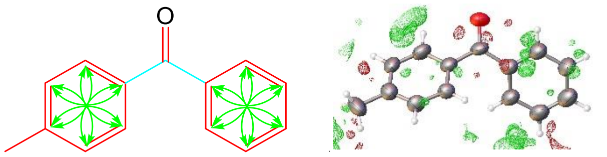

100% | SIMU

Figure S113 Geometric restraints and thermal ellipsoid (50% probability) for 50_10_96a Site D

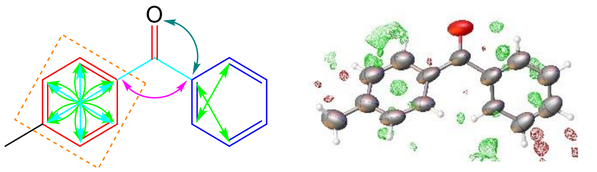

96.5% | SIMU | DANG 0.02 [O1E – C8E]

Figure S114 Geometric restraints and thermal ellipsoid (50% probability) for 50_10_96a Site E

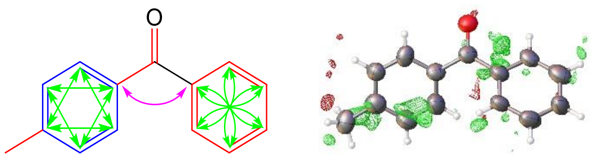

75.3% | SIMU | DANG 0.02 [C1F – C8F] | DFIX 0.01 [O1F – C7F]

Figure S115 Geometric restraints and thermal ellipsoid (50% probability) for 50_10_96a Site F

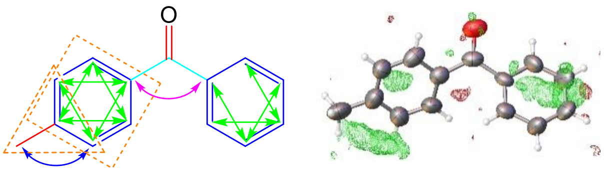

65.2% | SIMU

Figure S116 Geometric restraints and thermal ellipsoid (50% probability) for 50_10_96a Site G

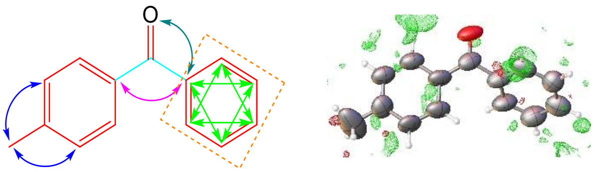

73.4% | SIMU | DFIX 0.01 [C4H – C14H] | DANG 0.02 [C1H – C8H] [C3H – C14H] [C5H – C14H]

Figure S117 Geometric restraints and thermal ellipsoid (50% probability) for 50_10_96a Site H

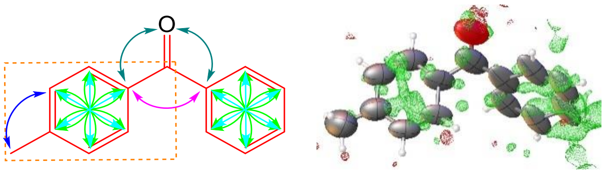

48.8% | SIMU + RIGU | DANG 0.02 [C1I – C8I] [O1I – C1I]

Figure S118 Geometric restraints and thermal ellipsoid (50% probability) for 50_10_96a Site I

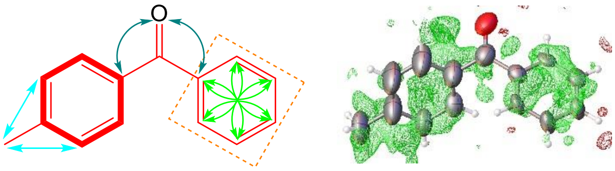

44.4% | SIMU + RIGU | DFIX [0.01 [O1J – C7J] | DANG 0.02 [O1J – C8J]

Figure S119 Geometric restraints and thermal ellipsoid (50% probability) for 50_10_96a Site J

Table S116 4-Methylbenzophenone 50_10_96b Crystallographic Table

CCDC Deposition Number	2343731
Empirical formula	$C_{36}H_{24}I_6N_{12}Zn_3$
Formula weight	1582.18
Temperature/K	100.00(10)
Crystal system	monoclinic
Space group	C2/c
a/Å	35.2498(4)
b/Å	14.90120(10)
c/Å	31.3590(4)
α = γ /°	90
β/°	102.4170(10)
Volume/Å^3	16086.5(3)
Z	8
ρ_{calc}g/cm^3	1.307
μ/mm^{-1}	19.316
F(000)	5856
Crystal size/mm^3	0.12 × 0.09 × 0.06
Radiation	Cu Kα (λ = 1.54184)
2Θ range for data collection/°	5.134 to 136.498
Index ranges	-41 ≤ h ≤ 42, -17 ≤ k ≤ 16, -37 ≤ l ≤ 35
Reflections collected	68924
Independent reflections	14633 [R_{int} = 0.0222, R_{sigma} = 0.0185]
Data/restraints/parameters	14633/21/598
Goodness-of-fit on F^2	1.088
Final R indexes [I>=2σ (I)]	R_1 = 0.0302, wR_2 = 0.0925
Largest diff. peak/hole / e Å^{-3}	0.50/-0.36
No guests or solvent were identified from the electron density map, SQUEEZE analysis indicated one void with a volume of 1021Å^3 and electron count of 293. This is equivalent to 6.10 cyclohexane molecules or 2.82 4-methylbenzophenone molecules.	

Table S117 4-Methylbenzophenone 50_10_96c Crystallographic Table

CCDC Deposition Number	2343732
Empirical formula	$C_{95.17}H_{67.86}I_{12}N_{24}O_{1.66}Zn_6$
Formula weight	3489.13
Temperature/K	100.00(10)
Crystal system	triclinic
Space group	P-1
a/Å	15.0552(6)
b/Å	18.9430(7)
c/Å	30.8258(9)
α /°	99.996(3)
β/°	93.338(3)
γ /°	110.091(3)
Volume/Å^3	8064.7(5)
Z	2
ρ_{calc}g/cm^3	1.437
μ/mm^{-1}	19.329
F(000)	3272
Crystal size/mm^3	0.31 × 0.12 × 0.08
Radiation	Cu Kα (λ = 1.54184)
2Θ range for data collection/°	5.076 to 136.502
Index ranges	-18 ≤ h ≤ 17, -22 ≤ k ≤ 22, -34 ≤ l ≤ 37
Reflections collected	113925
Independent reflections	29370 [R_{int} = 0.0857, R_{sigma} = 0.0581]
Data/restraints/parameters	29370/392/1440
Goodness-of-fit on F^2	1.031
Final R indexes [I>=2σ (I)]	R_1 = 0.1831, wR_2 = 0.5043
Largest diff. peak/hole / e Å^{-3}	3.49/-2.01
SQUEEZE analysis indicated one void with a volume of 1409Å^3 and electron count of 394. This is equivalent to 8.21 cyclohexane molecules or 3.79 4-methylbenzophenone molecules. Manual weighting scheme applied.	

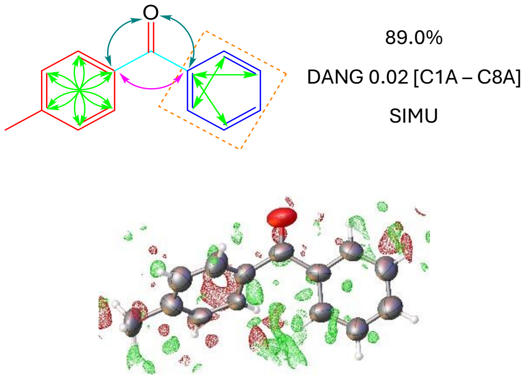

Figure S120 Geometric restraints and thermal ellipsoid (50% probability) for 50_10_96c Site A

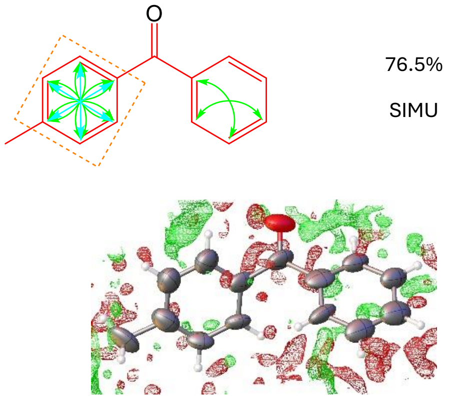

Figure S121 Geometric restraints and thermal ellipsoid (50% probability) for 50_10_96c Site B

S15. 4-Methylbenzophenone Analyte Grades and Discussion

Table S118 4-Methylbenzophenone exchange site and average grade full tabulation.

Dataset	Site A	Site B	Site C	Site D	Site E	Site F	Site G	Site H	Site I	Site J	Average
4_10_96a	-	-	-	-	-	-	-	-	-	-	-
4_10_96b	-	-	-	-	-	-	-	-	-	-	-
4_10_96c	-	-	-	-	-	-	-	-	-	-	-
50_10_96a	1.50	1.69	3.25	3.34	3.69	3.88	4.44	5.19	10.00	10.13	4.71
50_10_96b	-	-	-	-	-	-	-	-	-	-	-
50_10_96c	4.13	5.06	-	-	-	-	-	-	-	-	4.59

While the 50°C experiments enabled full characterisation of 4-methylbenzophenone, the structures could likely be further optimised. One feature which would greatly improve the quality of the overall data is the reduction of ZnI_2 unit disorder which resulted in particularly high residual electron density peaks. If this density could be localised more efficiently, then weaker structural features may be identified, and the overall quality of all guest sites would likely improve. One experimental approach which may enable this is the use of an intermediate lower temperature 'resting' period, employed for α-santonin, which could help the host-guest system to settle into more consistent positions. (Hoshino *et al.*, 2016)

Although experiments at 4°C were unable to model any guest molecules, it is thought that the guest exchange did occur but without suitable long-range ordering. This was evidenced by three observations: expansion of the unit cell volume to ~16.2k Å^3, expansion of the unit cell β angle to ~102°, and high residual electron density within the framework cavities. This suggests that the exchange sites adopted by 50°C structures possess a kinetic barrier which must be overcome.

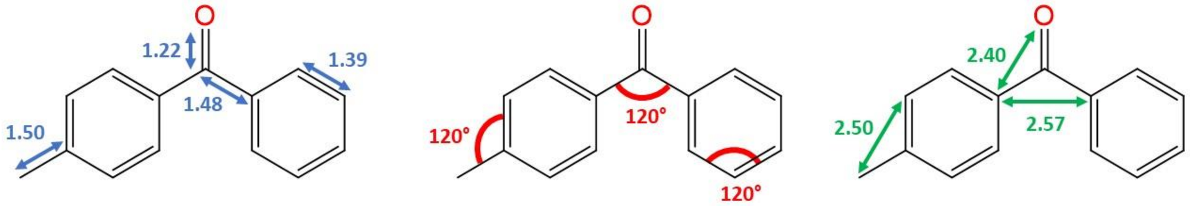

Figure S122 Geometric values for 4-methylbenzophenone determined by traditional SCXRD study. (Kutzke *et al.*, 1996)

S16. References

Dolomanov, O. V., Bourhis, L. J., Gildea, R. J., Howard, J. A. K. & Puschmann, H. (2009). *J. Appl. Cryst.* **42**, 339–341.

Groom, C. R., Bruno, I. J., Lightfoot, M. P. & Ward, S. C. (2016). *Acta Crystallogr. Sect. B Struct. Sci. Cryst. Eng. Mater.* **72**, 171–179.

Hoshino, M., Khutia, A., Xing, H., Inokuma, Y. & Fujita, M. (2016). *IUCrJ* **3**, 139–151.

Kutzke, H., Al-Mansour, M. & Klapper, H. (1996). *J. Mol. Struct.* **374**, 129–135.

Sheldrick, G. M. (2015*a*). *Acta Crystallogr.* **C71**, 3–8.

Sheldrick, G. M. (2015*b*). *Acta Crystallogr.* 3–8.

Spek, A. L. (2003). *J. Appl. Crystallogr.* **36**, 7–13.
